# Supplementary material for: Identifying stably expressed genes from multiple RNA-Seq data sets
Source: PeerJ. 2016 Dec 20;4:e2791. doi: 10.7717/peerj.2791 (PMC5178351; doi:10.7717/peerj.2791)
Supplement: Table S1 — Summary of data sets used in the three groups: the seedling, the leaf and the multi-tissue groups. [file peerj-04-2791-s001.pdf]

# Supplementary Table S1

Table S1: Summary of data sets used in the three groups: the seedling, the leaf and the multi-tissue groups.

| GEO Number | Reference | Tissue | N | Quality[1]($\geq$) |
|---|---|---|---|---|
| GSE32202 | NA | seedling | 6 | 84.30% |
| GSE37159 | NA | seedling | 8 | 82.50% |
| GSE41766 | [1] | seedling | 6 | 85.20% |
| [2]GSE43865 | [18] | seedling | 6 | 92.50% |
| GSE51119 | [21] | seedling | 10 | 70.50% |
| GSE51772 | [16] | seedling | 8 | 90.60% |
| GSE53078 | [6] | seedling | 4 | 86.10% |
| [3]GSE60835 | [5] | seedling | 6 | 65.60% |
| GSE66666 | [3] | seedling | 6 | 91.20% |
| GSE36626 | [20] | leaves | 4 | 85.50% |
| [4]GSE39463 | [12] | leaves | 12 | 74.50% |
| GSE48235 | [10] | leaves | 6 | 90.40% |
| [5]GSE51304 | [19] | leaves | 18 | 88.40% |
| [6]GSE54677 | [14] | leaves | 20 | 85.20% |
| GSE35288 | [15] | flower | 6 | 76.50% |
| GSE35408 | [2] | hypocotyl | 10 | 77.00% |
| GSE52966 | [4] | primary root | 18 | 87.20% |
| GSE56326 | NA | carpels | 8 | 92.40% |
| GSE59167 | [17] | root tip tissue | 11 | 87.10% |
| GSE59637 | [13] | inflorescences and siliques | 4 | 71.70% |
| GSE60183 | [8] | epidermis | 6 | 62.30% |
| GSE61061 | [11] | seed | 6 | 91.60% |
| GSE62799 | [7] | aerial tissue | 6 | 89.30% |
| GSE63355 | [9] | shoot apical meristem | 16 | 87.90% |

[1]The number of mapped reads divided by the total number of reads in the sample

[2]We chose all the 6 samples at the onset of treatment out of the 42 samples.

[3]We chose the samples of ecotype Columbia from the 12 samples.

[4]We chose all samples at 6 hours post inoculation out of the 48 samples.

[5]Out of the 48 samples, the total number of RNA-Seq samples is 18.

[6]Out of the 24 samples, the total number of RNA-Seq samples is 20.

# References


[1] M.-Y. Bai, M. Fan, E. Oh, and Z.-Y. Wang. A triple helix-loop-helix/basic helix-loop-helix cascade controls cell elongation downstream of multiple hormonal and environmental signaling pathways in arabidopsis. *The Plant Cell*, 24(12):4917–4929, 2012.

[2] M.-Y. Bai, J.-X. Shang, E. Oh, M. Fan, Y. Bai, R. Zentella, T.-p. Sun, and Z.-Y. Wang. Brassinosteroid, gibberellin and phytochrome impinge on a common transcription module in arabidopsis. *Nat. Cell Biol.*, 14(8):810–817, 2012.

[3] M. Capella, P. A. Ribone, A. L. Arce, and R. L. Chan. Arabidopsis thaliana HomeoBox 1 (AtHB1), a Homedomain-Leucine Zipper I (HD-Zip I) transcription factor, is regulated by PHYTOCHROME-INTERACTING FACTOR 1 to promote hypocotyl elongation. *New Phytol.*, 207(3):669–682, 2015.

[4] J. Chaiwanon and Z.-Y. Wang. Spatiotemporal brassinosteroid signaling and antagonism with auxin pattern stem cell dynamics in arabidopsis roots. *Curr. Biol.*, 25(8):1031–1042, 2015.

[5] J. Dong, D. Tang, Z. Gao, R. Yu, K. Li, H. He, W. Terzaghi, X. W. Deng, and H. Chen. Arabidopsis de-etiolated1 represses photomorphogenesis by positively regulating phytochrome-interacting factors in the dark. *The Plant Cell*, 26(9):3630–3645, 2014.

[6] M. Fan, M.-Y. Bai, J.-G. Kim, T. Wang, E. Oh, L. Chen, C. H. Park, S.-H. Son, S.-K. Kim, M. B. Mudgett, et al. The bhlh transcription factor hbi1 mediates the trade-off between growth and pathogen-associated molecular pattern–triggered immunity in arabidopsis. *The Plant Cell*, 26(2):828–841, 2014.

[7] M. Groth, H. Stroud, S. Feng, M. V. Greenberg, A. A. Vashisht, J. A. Wohlschlegel, S. E. Jacobsen, and I. Ausin. SNF2 chromatin remodeler-family proteins FRG1 and-2 are required for RNA-directed DNA methylation. *P. Natl. Acad. Sci. USA*, 111(49):17666–17671, 2014.

[8] Y. Kimura, S. Aoki, E. Ando, A. Kitatsuji, A. Watanabe, M. Ohnishi, K. Takahashi, S.-i. Inoue, N. Nakamichi, Y. Tamada, et al. A flowering

integrator, soc1, affects stomatal opening in arabidopsis thaliana. *Plant Cell Physiol.*, 56(4):640–649, 2015.

[9] C.-H. Liu, A. Finke, M. Díaz, W. Rozhon, B. Poppenberger, T. Baubec, and A. Pecinka. Repair of dna damage induced by the cytidine analog zebularine requires atr and atm in arabidopsis. *The Plant Cell*, 27(6):1788–1800, 2015.

[10] N. Liu, Y. Ding, M. Fromm, and Z. Avramova. Different gene-specific mechanisms determine the revised-responsememory transcription patterns of a subset of A. thaliana dehydration stress responding genes. *Nuc. Acids Res.*, 42(9):5556–5566, 2014.

[11] D. R. MacGregor, S. L. Kendall, H. Florance, F. Fedi, K. Moore, K. Paszkiewicz, N. Smirnoff, and S. Penfield. Seed production temperature regulation of primary dormancy occurs through control of seed coat phenylpropanoid metabolism. *New Phytol.*, 205(2):642–652, 2015.

[12] T. Maekawa, B. Kracher, S. Vernaldi, E. V. L. van Themaat, and P. Schulze-Lefert. Conservation of NLR-triggered immunity across plant lineages. *P. Natl. Acad. Sci. USA*, 109(49):20119–20123, 2012.

[13] C. Mizzotti, I. Ezquer, D. Paolo, P. Rueda-Romero, R. F. Guerra, R. Battaglia, I. Rogachev, A. Aharoni, M. M. Kater, E. Caporali, et al. SEEDSTICK is a master regulator of development and metabolism in the Arabidopsis seed coat. *PLoS Genet.*, 10(12):e1004856, 2014.

[14] G. Moissiard, S. Bischof, D. Husmann, W. A. Pastor, C. J. Hale, L. Yen, H. Stroud, A. Papikian, A. A. Vashisht, J. A. Wohlschlegel, et al. Transcriptional gene silencing by Arabidopsis microrchidia homologues involves the formation of heteromers. *P. Natl. Acad. Sci. USA*, 111(20):7474–7479, 2014.

[15] C. E. Niederhuth, O. R. Patharkar, and J. C. Walker. Transcriptional profiling of the Arabidopsis abscission mutant hae hsl2 by RNA-Seq. *BMC Genomics*, 14(1):37, 2013.

[16] E. Oh, J.-Y. Zhu, M.-Y. Bai, R. A. Arenhart, Y. Sun, and Z.-Y. Wang. Cell elongation is regulated through a central circuit of interacting transcription factors in the arabidopsis hypocotyl. *Elife*, 3:e03031, 2014.

[17] H. Pallakies and R. Simon. The CLE40 and CRN/CLV2 signaling pathways antagonistically control root meristem growth in arabidopsis. *Mol. Plant*, 7(11):1619–1636, 2014.

[18] M. L. Rugnone, A. F. Soverna, S. E. Sanchez, R. G. Schlaen, C. E. Hernando, D. K. Seymour, E. Mancini, A. Chernomoretz, D. Weigel, P. Más, et al. Lnk genes integrate light and clock signaling networks at the core of the arabidopsis oscillator. *P. Natl. Acad. Sci. USA*, 110(29):12120–12125, 2013.

[19] H. Stroud, T. Do, J. Du, X. Zhong, S. Feng, L. Johnson, D. J. Patel, and S. E. Jacobsen. Non-CG methylation patterns shape the epigenetic landscape in Arabidopsis. *Nat. Struct. Mol. Biol.*, 21(1):64–72, 2014.

[20] H. Wollmann, S. Holec, K. Alden, N. D. Clarke, P.-E. Jacques, and F. Berger. Dynamic deposition of histone variant H3. 3 accompanies developmental remodeling of the Arabidopsis transcriptome. *PLoS Genet.*, 8(5):e1002658, 2012.

[21] M. K. Zhiponova, K. Morohashi, I. Vanhoutte, K. Machemer-Noonan, M. Revalska, M. Van Montagu, E. Grotewold, and E. Russinova. Helix–loop–helix/basic helix–loop–helix transcription factor network represses cell elongation in Arabidopsis through an apparent incoherent feed-forward loop. *P. Natl. Acad. Sci. USA*, 111(7):2824–2829, 2014.
