# Supplementary material for: Identifying stably expressed genes from multiple RNA-Seq data sets
Source: PeerJ. 2016 Dec 20;4:e2791. doi: 10.7717/peerj.2791 (PMC5178351; doi:10.7717/peerj.2791)
Supplement: Table S2 [file peerj-04-2791-s002.pdf]

Supplemental Table S2: Top 1000 stably expressed genes for the seedling group.

Gene	between_sample	bewtween_treatment	between_experiment	Rank
AT3G03940	0.00116053	0.003173212	0	1
AT2G45810	0.00083305	0.002335395	0.001935448	2
AT1G30470	0.000735233	0.002322831	0.002229492	3
AT4G24490	0	0.000827779	0.004496324	4
AT1G35470	0.001245486	0.002212708	0.001901545	5
AT3G60800	0.002782435	0.001740079	0.001140532	6
AT1G17720	0.001236412	0.000763282	0.003688971	7
AT5G08200	0.00228709	0.001572528	0.00196603	8
AT3G51620	0.002513118	0.002154863	0.001401293	9
AT1G64385	0.000798032	0.002130628	0.003362661	10
AT3G63150	0.000928687	0.001651687	0.003724528	11
AT2G47210	0	0.002956423	0.003593594	12
AT3G01610	0.001969248	0.000227854	0.004530186	13
AT5G54750	0.004155016	0.002752933	0	14
AT3G21160	0	0.003449392	0.003501691	15
AT5G63870	0.000967228	0.004044457	0.002182291	16
AT2G35330	0.001983305	0.001427244	0.003803674	17
AT3G25800	0.002063067	0.00171959	0.003538277	18
AT2G06210	0.002805866	0	0.004824157	19
AT5G41770	0.001211875	0.003239827	0.003287039	20
AT2G39630	0.004487882	0.001845234	0.001413295	21
AT2G47980	0.001084715	0.00101191	0.006131066	22
AT1G80950	9.24E-06	0.005110881	0.003110542	23
AT3G09100	0.001778554	0.003036307	0.003458495	24
AT4G33650	0.002204082	0.001576012	0.004545821	25
AT3G02950	0.004012348	0.002253635	0.002065758	26
AT1G48040	0.00039779	0.005575506	0.002586029	27
AT1G71350	0.002491509	0.002114429	0.004170339	28
AT1G22200	0.001626372	0.001864889	0.005319075	29
AT5G18420	0.00059691	0.002991661	0.005336844	30
AT5G15080	0.002140895	0.004122283	0.002722939	31
AT5G20920	0.005698159	7.27E-05	0.003376983	32
AT1G63700	0.004044561	0.001654803	0.003450652	33
AT5G18190	0.001137347	0.003227287	0.004807082	34
AT2G32730	0.003171896	2.85E-05	0.006233419	35
AT4G26640	0.000621016	0.003970216	0.004893894	36
AT5G49580	0.00577706	0.002578846	0.001228563	37
AT3G20890	0.001290476	0	0.008468825	38
AT2G20930	0.009770724	0	0	39

AT1G54610	0.002679662	0.00245221	0.004684585	40
AT2G36480	0.001250528	0.001634904	0.006940299	41
AT4G34430	0.003201314	0.001362268	0.005268066	42
AT3G26400	0.001819019	0.003029618	0.004987337	43
AT3G06940	0.005605037	0.001718891	0.002524584	44
AT5G47010	0.003526772	0.00096314	0.005374586	45
AT4G17510	0.001903944	0.001745387	0.006242614	46
AT1G54460	0.002055782	2.65E-05	0.007828902	47
AT2G30880	8.59E-05	0.002023707	0.007811242	48
AT1G61150	0.005285475	0.002139758	0.002523491	49
AT4G25515	0.003945182	0.002192176	0.003824628	50
AT1G25380	0.001525049	0.003369999	0.005108425	51
AT1G45233	0.005304547	0.00229324	0.002412612	52
AT3G48430	0.008288109	0	0.001793721	53
AT1G75420	0	0.00555225	0.004587282	54
AT5G53000	0.000440553	0.005320187	0.004436682	55
AT3G60190	0.000674736	0.005188315	0.004339294	56
AT3G20650	0.003326419	0	0.006914188	57
AT3G22520	0.002701657	0.00069508	0.006862462	58
AT4G15840	0.001437243	0.002445857	0.006439781	59
AT3G14290	0.003687986	0.002852668	0.003815791	60
AT4G38170	0.006957551	0.000183551	0.003367022	61
AT5G06680	0.003218396	0.00456336	0.002752524	62
AT4G20400	0.002951533	0.000149529	0.00744503	63
AT1G31870	0.002150571	0.00273598	0.005700086	64
AT1G12270	0.001712733	0.002879562	0.005998655	65
AT5G52530	0.003049218	0.001563301	0.005983596	66
AT5G17770	0.00180003	0.002382426	0.006461963	67
AT1G65020	0.002541466	0.002875498	0.00525891	68
AT4G28600	0.000710053	0.003653187	0.006335567	69
AT1G78800	0.002065805	0.002824634	0.005874269	70
AT4G33945	0.00073749	0.004246439	0.005886684	71
AT1G65220	0.000554432	0.003552673	0.006805018	72
AT2G19160	0.003233059	0.001845019	0.005845282	73
AT1G03750	0.00548073	0.005456431	0	74
AT5G64470	0.002135011	0.00254521	0.006284483	75
AT2G23080	0.002854112	0.001544392	0.006570721	76
AT3G20720	0.002302112	0.00513326	0.003583355	77
AT4G32120	0.002705133	0.007957778	0.000378239	78
AT1G05520	0.002766567	0.002326369	0.005987908	79
AT4G29010	0.001232593	0.00425009	0.005629412	80
AT4G17640	0.000819714	0.007008119	0.003329942	81

AT4G04720	0.002647624	0	0.008580603	82
AT5G47760	0.001277824	0.005087809	0.004867945	83
AT1G76950	0.002662327	0.001789774	0.006810761	84
AT5G24360	0.000798116	0.002948611	0.007548276	85
AT5G67530	0.000550771	0.002586945	0.00821183	86
AT5G37380	0	0.005368658	0.006027721	87
AT4G13730	0.004623586	0.00045268	0.006346913	88
AT3G46220	0.001599742	0.000422196	0.009505463	89
AT4G12610	0.004394449	0.003558983	0.003716095	90
AT2G14850	0.001149703	0.001563852	0.008999916	91
AT5G09850	0.002627873	0.006594335	0.002514088	92
AT3G26370	0.001365032	0.00428616	0.006113679	93
AT5G16270	0.001824132	0.001427787	0.008516602	94
AT3G51100	0.002451112	0.000171662	0.0091602	95
AT1G67680	0.000406748	0.001847021	0.009581644	96
AT2G35110	0.005043934	0.001207291	0.005678184	97
AT5G17410	0.003755805	0.005617001	0.00257742	98
AT5G45620	0.003017256	0.004415366	0.004605876	99
AT5G21040	0.001263949	0.006464288	0.004376351	100
AT4G16360	0.001277612	0.00354399	0.007343803	101
AT1G69800	6.28E-05	0.007660616	0.004456346	102
AT1G03190	0.001070499	0.003268374	0.007842938	103
AT5G35430	0.002544092	0.002561092	0.007148307	104
AT3G18480	0.002374844	0.001225675	0.008662023	105
AT3G01090	0.000851943	0.00510614	0.006400858	106
AT1G69340	0.000957951	0.002123247	0.009278742	107
AT5G11730	0.002672289	0.006791889	0.002918165	108
AT5G50870	0.00270893	0.008316655	0.001363856	109
AT4G01810	0.001819483	0.004805317	0.005829884	110
AT1G60490	0.000810438	0.005490777	0.006192018	111
AT5G23550	0.004968458	0.00018257	0.007405713	112
AT1G16010	0.001283206	0.007567477	0.003708391	113
AT2G25300	0.005798977	0.004170108	0.00259304	114
AT1G28490	0.001577326	0.002370388	0.00867326	115
AT4G24470	0.000865423	0.002008521	0.009839124	116
AT3G17250	0.000283098	0.012440245	0	117
AT1G11660	0.003308092	0.005773099	0.00370235	118
AT1G21700	0.002407349	0.002160733	0.008221974	119
AT5G11710	0.000962071	0.004732794	0.007113299	120
AT4G39370	0.002786063	0.003889865	0.0061336	121
AT1G56590	0.002997679	3.99E-05	0.009791337	122
AT1G48050	0.000566683	0.003804056	0.008501392	123

AT5G38560	0.000903759	0.003937589	0.00805972	124
AT4G24820	0.002960581	0.000806381	0.00914055	125
AT5G35570	0.003705614	0.001563238	0.007642363	126
AT1G79570	0.002547103	0.003281602	0.007120283	127
AT1G01800	0.002061322	0.006918902	0.003986843	128
AT2G44970	0.002118662	0.002644492	0.008224227	129
AT3G50360	0.002247757	0.004133832	0.006614867	130
AT2G32910	0.002681612	0.002428592	0.007943909	131
AT5G25150	0.002110398	0.000362693	0.010619173	132
AT3G09670	0.005987088	0	0.007143661	133
AT3G61050	0.000703652	0.003658425	0.008779369	134
AT2G26990	0.001571244	0.002977736	0.008609902	135
AT2G01720	0.002196245	0.000811131	0.010164318	136
AT1G26120	0.001930166	0.001913141	0.009389779	137
AT2G41900	0.002027767	0.00233838	0.008872956	138
AT3G55620	0.002356501	0.005831748	0.005062804	139
AT4G17270	0.001118591	0.004928093	0.00722985	140
AT2G42700	0.001799642	0.002705372	0.008838647	141
AT2G46180	0.004866121	0.000274645	0.008213015	142
AT2G30710	0.001947717	0.001230868	0.01019028	143
AT1G16270	0.003103071	0.002385316	0.007897883	144
AT5G46750	0.002397114	0.005390242	0.005666908	145
AT5G19680	0.005628611	0.001022663	0.006890449	146
AT2G20790	0.00215191	0.004403367	0.006992	147
AT2G43260	0.000870597	0.003257734	0.009447286	148
AT1G68160	0.007399341	0.001683869	0.004514533	149
AT1G26370	0.000865625	0.002170753	0.010561714	150
AT3G57910	0.009603764	0	0.004045152	151
AT2G05840	0.002685476	0.004134502	0.006839868	152
AT2G44710	0.001738391	0.002963912	0.00896722	153
AT5G12290	0.002966574	0.003578392	0.007164281	154
AT3G18790	0.005098678	0.001210014	0.007481339	155
AT1G68720	0.003234546	0.002900689	0.007658611	156
AT5G51280	0.002887524	0.002220599	0.00871192	157
AT2G28520	0.000475439	0.001895087	0.011487516	158
AT1G13320	0.002219954	0.001387894	0.010272506	159
AT3G17900	0.001838528	0.002392309	0.009658498	160
AT5G01310	0.00172233	0.004121269	0.008074175	161
AT5G63940	0.001670315	0.008579164	0.003684412	162
AT3G15660	0.002653243	0.002270334	0.009055595	163
AT3G58130	0.00460349	0.00362838	0.005765691	164
AT4G10570	0.000967851	0.002276084	0.010798203	165

AT5G11430	0.005758837	0.002733322	0.005618147	166
AT4G08350	0.001687345	0.001664504	0.010769422	167
AT5G40300	0.006435869	0.001164704	0.006530375	168
AT1G21720	0.002472489	0.000911677	0.010768761	169
AT2G47330	0.001332912	0.003248015	0.009616146	170
AT1G76940	0.002344329	0.002140515	0.009750514	171
AT3G10330	0.001428806	0.004848682	0.007970604	172
AT2G46700	0.002383257	0.001389125	0.010508427	173
AT1G01910	0.002301917	0.004707579	0.007302247	174
AT4G15880	0.0001555	0.003693419	0.010475375	175
AT3G51980	0.003996052	0.004957564	0.005431572	176
AT4G17020	0.007209229	0.003502089	0.003677883	177
AT3G13290	0.00380518	0.003117057	0.007470993	178
AT2G48160	0.005805124	0	0.00861745	179
AT1G09840	0.001436837	0.002966771	0.010051978	180
AT4G13360	0.001153621	0.005809802	0.007502813	181
AT2G24640	0.003990951	0.005557595	0.00492638	182
AT1G21170	0.001137676	0.002522303	0.010820212	183
AT3G11910	0.002351052	0.00099849	0.011184811	184
AT5G13560	0.000458692	0.001184832	0.012918649	185
AT3G10650	0.003251079	0.005279074	0.006114863	186
AT1G61670	0.001383998	0.004852008	0.008417266	187
AT1G53050	0.000929102	0.002884616	0.010852417	188
AT5G59710	0.000260963	0.004890389	0.00952211	189
AT4G24560	0.002485058	0.001397706	0.01080039	190
AT3G25580	0.004364585	0	0.010327786	191
AT2G36330	0.004369094	0.010389359	0	192
AT5G57015	0.004522973	5.53E-05	0.010198352	193
AT4G29490	0.001232226	0.001202463	0.012346296	194
AT1G75990	0.002345596	0.003674186	0.008767536	195
AT3G57090	0.00113042	0.004944747	0.008775259	196
AT1G49710	0.002014147	0.005848267	0.007005096	197
AT2G02560	0.001658726	0.001811274	0.011486389	198
AT3G06240	0.000868227	0.006629467	0.007476564	199
AT1G50380	0.001897161	0.00757806	0.005510799	200
AT1G55170	0.004770726	0.005707976	0.004542652	201
AT2G27460	0.00456381	0.003339966	0.007171926	202
AT2G16860	0.005217084	0.005118625	0.004792245	203
AT2G32070	0.002982021	0.005520509	0.006656487	204
AT2G45500	0.00217973	0.007326668	0.005667323	205
AT2G11520	0.00129549	0.010825238	0.003057228	206
AT5G19130	0.002765439	0.010991621	0.001421968	207

AT5G35590	0.001780756	0.003376981	0.010090698	208
AT2G30980	0.001042633	0.005618464	0.008587733	209
AT5G58450	0.001972199	0.005151424	0.008144182	210
AT5G28740	0.002769744	0.004584205	0.007928656	211
AT2G44820	0.004179812	0.00852721	0.002592689	212
AT5G51430	0.002381938	0.007773656	0.005145357	213
AT5G58270	0.001403877	0.002888132	0.011020179	214
AT1G17130	0.002883807	0.004120809	0.008324323	215
AT2G32850	0.00186309	0.002196164	0.011280469	216
AT3G61800	0.01063447	0	0.004719172	217
AT5G65000	0.002125962	0.005033523	0.008198687	218
AT1G34270	0.001271872	0.007278605	0.006818256	219
AT4G01370	0.00182402	0.006550692	0.00700387	220
AT5G53530	0.00419423	0.00621251	0.004983376	221
AT3G50590	0.003010007	0.004864398	0.007543022	222
AT1G76140	0.002065339	0.003293295	0.010083949	223
AT3G16110	0.003031782	0.00623278	0.006180409	224
AT2G20300	0.001980778	0.002368638	0.011135804	225
AT5G06750	0.003896731	0.008213792	0.003380606	226
AT3G28670	0	0.006545979	0.008958062	227
AT1G04230	0.004742985	0.004855592	0.005953476	228
AT1G71270	0.000289416	0.002412415	0.012850265	229
AT5G49510	0.004403278	0.004365664	0.006814828	230
AT2G20310	0.00481441	0.001480104	0.0092926	231
AT3G20800	0.002784496	0.006022598	0.006800094	232
AT2G22010	0.006165315	0.001304447	0.008150281	233
AT4G30890	0.001537554	0.005598258	0.008513298	234
AT2G20000	0.00382587	0.000799962	0.011030438	235
AT4G09340	0.003438583	0.010168378	0.00205523	236
AT1G32330	0.002664062	0.004048936	0.008962236	237
AT5G19010	0.002015364	0.007573924	0.006093564	238
AT1G17270	0.009807832	0.002100241	0.003782355	239
AT3G11400	0.003500309	7.53E-05	0.012133708	240
AT1G12470	0.003357247	0.000230317	0.012181721	241
AT3G18860	0.001160735	0.004647246	0.009981206	242
AT5G11980	0.001312065	0.008620236	0.005861705	243
AT5G07980	0.004709794	0.001809243	0.009276767	244
AT2G39960	0.0038303	0.000619199	0.011366484	245
AT3G17430	0.001521402	0.005490992	0.008822209	246
AT2G17410	0.002920629	0.000169086	0.012750757	247
AT1G54170	0.000805268	0.001870394	0.013211807	248
AT2G30120	0.003914392	0.006373681	0.00561029	249

AT5G58003	0.003082302	0.002736445	0.010113705	250
AT5G20200	0.004412876	0.003409773	0.008113616	251
AT3G23900	0.00676601	0	0.009176386	252
AT1G75510	0.001149527	0.004935453	0.009861514	253
AT4G27690	0.001346394	0.003999722	0.01060463	254
AT3G53390	0.002463152	0.002001759	0.011489712	255
AT3G20290	0.00040618	0.00307492	0.012497904	256
AT2G47620	0.001794698	0.003491906	0.010722718	257
AT1G20200	0.000668418	0.002257098	0.013092304	258
AT1G48110	0.00079353	0.003217923	0.01203228	259
AT2G06000	0.001912626	0.009574974	0.004572428	260
AT4G38360	0.002493914	0.003103813	0.010465018	261
AT4G22140	1.29E-17	0.004707579	0.01145088	262
AT3G26730	0.004178148	0.002705974	0.009294288	263
AT4G00420	0.000780107	0.004806619	0.01061794	264
AT2G25560	0.003250997	0.003397633	0.009620578	265
AT3G57570	0.002737888	0.001980612	0.011555005	266
AT5G42140	0.002267612	0.005615738	0.008402255	267
AT2G48110	0.005613021	0.00184757	0.008907769	268
AT1G19120	0.002256027	0.000890027	0.013228463	269
AT1G30480	0.005864215	0	0.010533034	270
AT5G05520	0.003119874	0.005131555	0.008179129	271
AT1G57620	0.005076715	0.008406987	0.002949287	272
AT3G23660	0.002743874	0.001836491	0.011895809	273
AT4G24550	0.000854772	0.001677506	0.013943947	274
AT4G24200	0.00416646	0.002672126	0.009705326	275
AT1G56610	0.001114013	0.005363851	0.010066874	276
AT1G31020	0.011814012	0	0.004759446	277
AT1G17820	0.005175794	0.007704886	0.003716088	278
AT1G16470	0.003491197	0.002579435	0.010530799	279
AT4G11790	0.002074549	0.001225046	0.013347176	280
AT5G04420	0.00037329	0.003604886	0.012736442	281
AT5G56280	0.000940992	0.006114572	0.009679827	282
AT4G02020	0.001080506	0.003112107	0.012552145	283
AT3G03740	0.001735719	0.00598603	0.009023286	284
AT1G10430	0.00352997	0.004899613	0.008328192	285
AT1G52360	0.001719126	0.004007603	0.011064716	286
AT5G16280	0.001914673	0.003200604	0.011741595	287
AT3G06250	0.001044481	0.007289313	0.008553933	288
AT2G40090	0.001034391	0.004691529	0.011185615	289
AT5G57860	0.007361289	0.009590222	0	290
AT1G07960	0.003723288	0.003213894	0.010020752	291

AT2G05755	0.003447393	0.000220771	0.013292383	292
AT2G44530	0.002620427	0.003043263	0.011300722	293
AT3G48820	0.003008538	0.003959163	0.010004638	294
AT2G19390	0.009346108	0.002997405	0.00463791	295
AT3G06580	0.001226018	0.006527465	0.009235384	296
AT1G04850	0.001910706	0.004102796	0.010995311	297
AT5G21010	0.004538216	0.000680295	0.011793356	298
AT4G23640	0.00177317	0.002799972	0.012450784	299
AT2G35530	0.001907713	7.80E-05	0.015039881	300
AT3G13530	0.002486996	0.003384263	0.011191883	301
AT3G19770	0.001485084	0.004548203	0.011042702	302
AT4G26455	0.002986008	0.000466979	0.013687904	303
AT2G26590	0.002543072	0.003425775	0.011184188	304
AT3G51850	0.00155878	0.004367799	0.011245952	305
AT5G54440	0.002015517	0.003283244	0.01188226	306
AT5G65780	0.00270007	0.002552793	0.011928161	307
AT5G60160	0.001435941	0.001706364	0.014072143	308
AT2G18770	0.004869607	0.003090298	0.009269396	309
AT5G11810	0.005389372	0.002749197	0.009116008	310
AT4G16440	0.00650733	0	0.010750596	311
AT3G60740	0.002724914	0.00486347	0.00972405	312
AT3G18430	0.00583294	0.001992785	0.009518091	313
AT3G06350	0.002044546	0.010465594	0.004847419	314
AT5G07300	0.001882007	0.007373292	0.008111111	315
AT5G06260	0.00254022	0.001437911	0.013395388	316
AT2G27350	0.000942857	0.001157367	0.015291548	317
AT3G61240	0.000572183	0.000702914	0.01616473	318
AT2G17700	0.002490095	0.011865784	0.003144723	319
AT1G52380	0.004185999	0.003852287	0.00947841	320
AT5G38830	1.48E-05	0.00717419	0.010368306	321
AT1G77140	0.0013686	0.00387048	0.012325013	322
AT5G41870	0.005296244	0.008203913	0.004068953	323
AT1G55090	0.003734712	0.003354493	0.010484416	324
AT3G27320	0.002044534	0.004462438	0.011066975	325
AT1G03560	0.001892954	0.009239719	0.006456787	326
AT5G56890	0.002132961	0.009337002	0.006126826	327
AT1G55620	0.003405148	0.002032381	0.012182057	328
AT4G14710	0.004854671	0.002515792	0.010269521	329
AT3G58510	0.001427084	0.002452179	0.013764824	330
AT2G01690	0.001017802	0.004344135	0.012289277	331
AT1G52630	0.000914527	0.006967248	0.00980333	332
AT4G30480	0.005354611	0.001221171	0.011141013	333

AT3G05090	0.001247055	0.003700107	0.012784942	334
AT4G31880	0.006221217	0.000490657	0.011040785	335
AT5G45330	0.010407377	0.004204484	0.003141284	336
AT5G44100	0.000774357	0.003955144	0.013151278	337
AT1G71440	0.001714794	0.003838966	0.012327088	338
AT5G49880	0.001284931	0.005298385	0.011327265	339
AT2G27110	0.000918043	0.003351468	0.013648035	340
AT5G27540	0.001463479	0.00266811	0.013794653	341
AT4G33890	0.001771542	0.004221407	0.011940647	342
AT2G41960	0.005969757	0	0.011969565	343
AT1G29220	0.002474657	0.004327872	0.011147718	344
AT1G17790	0.004651304	0.005492763	0.007817313	345
AT2G27285	0.013692954	0.000734583	0.003560736	346
AT4G35040	0.002343714	0.001987477	0.013659069	347
AT5G16890	0.003500698	0.007614079	0.006891799	348
AT2G33490	0.0043064	0.00528985	0.008415799	349
AT3G58460	0.000200738	0.002965738	0.014878188	350
AT3G27000	0.003678768	0.007410276	0.006956898	351
AT3G12380	0.001125585	0.001294614	0.015630601	352
AT4G35890	0.001279106	0.004131592	0.012654284	353
AT4G10730	0.003009959	0.007035962	0.00802047	354
AT1G03030	0.002591561	0.010420592	0.005056576	355
AT5G18230	0.002968236	0.001267846	0.013880163	356
AT1G02290	0.005197716	0.005048052	0.007889361	357
AT5G53930	0.00427486	0	0.013885145	358
AT4G26410	0.003135304	0.00031078	0.014716764	359
AT4G12640	0.005192505	0	0.012971061	360
AT3G11730	0.003308792	0.002798707	0.012063418	361
AT1G54080	0.001630368	0.002658061	0.013946073	362
AT5G22760	0.004304001	1.98E-05	0.013929462	363
AT2G27900	0.003277576	0.001221971	0.013756049	364
AT4G11270	0.000982064	0.002705718	0.014572141	365
AT5G58100	0.003002836	0.000718113	0.014539806	366
AT3G19280	0.00307742	0.004202938	0.010982807	367
AT3G14910	0.002715372	0.003255284	0.012310777	368
AT3G03570	0.004381415	0	0.013929181	369
AT4G39240	0.005668923	0	0.012673342	370
AT4G25880	0.001994365	0.001053036	0.015312504	371
AT1G05500	0.001048917	0.00397188	0.013363447	372
AT5G09830	0.009839368	0.00134893	0.007206565	373
AT5G65687	0.003606901	0.005118236	0.009673531	374
AT1G15490	0.003408928	0.002199023	0.012821216	375

AT5G67580	0.002614658	0.012161509	0.003678755	376
AT1G07980	0.005650606	0.004346222	0.008474076	377
AT3G21175	0.001460395	0.0037978	0.013216092	378
AT5G04740	0.001656313	0.012202193	0.004645236	379
AT5G07710	0.007057037	0.002758363	0.008718795	380
AT3G44340	0.003493234	0.003011705	0.012029296	381
AT4G32640	0.003182026	0.005831574	0.009526094	382
AT5G59210	0.003207245	0.007479067	0.007917036	383
AT4G30870	0.013726988	0.000782156	0.004145091	384
AT1G49820	0.003880495	0.005745171	0.009033622	385
AT4G04950	0.00078636	0.003056288	0.01482605	386
AT4G00500	0.003086718	0.007600755	0.007989717	387
AT2G34520	0.003761286	0.00383346	0.011091627	388
AT3G46180	0.001178062	0.007106121	0.01043464	389
AT1G06210	0.004957068	0.001312903	0.012455326	390
AT3G05280	0.001688658	0.004527867	0.01251349	391
AT1G18190	0.004179257	0.000859855	0.013712069	392
AT1G77620	0.00427509	0.000171341	0.014346201	393
AT5G22350	0.002824161	0.005933262	0.010036922	394
AT1G05180	0.005527519	0.007055991	0.006214019	395
AT4G17410	0.001117808	0.006372368	0.011331938	396
AT5G09860	0.001596114	0.003352747	0.013882717	397
AT1G64520	0.002931183	0.000925059	0.01499494	398
AT3G55000	0.004272797	0.004051956	0.010530777	399
AT3G04460	0.001932724	0.01104383	0.00588924	400
AT1G48380	0.005155833	0	0.013719981	401
AT2G27090	0.007420757	0.002140789	0.00934573	402
AT1G03330	0.004171918	0.006043151	0.008706655	403
AT1G09150	0.004405272	0.000929494	0.013650435	404
AT2G15530	0.003134972	0.003691519	0.012164908	405
AT3G45190	0.000325993	0.002864926	0.01581006	406
AT2G32170	0.003513088	0.001528542	0.013981307	407
AT4G10710	0.00542103	0.001317633	0.012300679	408
AT5G25080	0.002631795	0.005995852	0.010431824	409
AT2G36360	0.00366373	0.001572777	0.013846031	410
AT3G15920	0.002137146	0.003098822	0.013868216	411
AT2G16950	0.001749866	0.002659042	0.014703037	412
AT1G55325	0.004420234	0.003658895	0.011049781	413
AT3G61960	0.00020453	0.003972039	0.014952596	414
AT2G14120	0.001422304	0.00073259	0.017016063	415
AT3G07080	0.002169942	0.008191274	0.008835393	416
AT4G19180	0.0039234	0	0.015308378	417

AT3G07190	0.004313035	0.001952012	0.012967261	418
AT2G21270	0.00073128	0.004654003	0.013851268	419
AT1G27340	0.002384711	0.004006025	0.012849817	420
AT1G50500	0.002853389	0.004473876	0.011916126	421
AT2G27100	0.002203866	0.001581037	0.015475182	422
AT3G05290	0.005765535	0.009713441	0.003812833	423
AT5G16210	0.002858493	0.005829987	0.010604391	424
AT1G29820	0.004051675	0.008069977	0.007187231	425
AT1G74520	0.006597696	0.004063614	0.008670562	426
AT4G24530	0.003870576	0.00890407	0.006581578	427
AT4G32551	0.001466659	0.003717642	0.014179828	428
AT5G46070	0.00499754	0	0.014367775	429
AT5G05230	0.006167009	0.00590924	0.007313221	430
AT3G03140	0.001515207	0.004656817	0.013224184	431
AT1G10870	0.002040515	0.000441105	0.016914986	432
AT2G30410	0.007081156	0.0087883	0.0035575	433
AT2G11000	0.004964536	0.002052465	0.012423035	434
AT1G48270	0.006680931	0.007032518	0.005752915	435
AT4G33780	0.00528763	0.003020028	0.011211502	436
AT2G03120	0.001280219	0.004456183	0.013825025	437
AT1G62600	0.000257351	0.004812537	0.014495963	438
AT2G19080	0.004466813	0.004511262	0.010614295	439
AT5G63840	0.002552445	0.004416137	0.012631273	440
AT4G26100	0.000404926	0.00458136	0.014623408	441
AT4G39870	0.004480409	0.001048587	0.014088484	442
AT4G04885	0.003621374	0.001452288	0.014545408	443
AT1G06740	0.005991282	0	0.013629382	444
AT1G24460	0.009794242	0	0.00984303	445
AT5G57490	0.002184253	0.005254776	0.012204356	446
AT1G13690	0.006100528	0	0.01354726	447
AT5G09900	0.001555965	0.003116244	0.014995011	448
AT2G38840	0.004310446	0.004686458	0.010687608	449
AT3G47700	0.005153502	0.005130985	0.009430204	450
AT4G24390	0.002993137	0.003779698	0.012942959	451
AT3G07180	0.001703408	0.005380774	0.012654364	452
AT3G04610	0.002840197	0.008143669	0.008769679	453
AT5G13070	0.003605069	0.002547037	0.013606155	454
AT2G04630	0.005599732	0.000753365	0.013420706	455
AT3G60410	0.003463509	0.00679853	0.009529076	456
AT3G62220	0.003047404	0.010737973	0.006021381	457
AT5G08590	0.00209022	0.002877966	0.01484278	458
AT5G19420	0.000761725	0.005397456	0.013694243	459

AT3G22990	0.005745306	0.004030166	0.01011947	460
AT1G10390	0.002513382	0.003231951	0.014156076	461
AT4G25230	0.001358554	0.003250599	0.015295714	462
AT3G27325	0.00272582	0.00794276	0.009236419	463
AT1G10180	0.001316113	0.002928215	0.015672214	464
AT3G21640	0.003700063	0.00712719	0.009112756	465
AT3G18380	0.006236035	0	0.013726193	466
AT4G39520	0.001764256	0.005177133	0.013021245	467
AT5G15920	0.002555379	0.005228717	0.012205096	468
AT2G14740	0.001505751	0.004113025	0.014373663	469
AT4G36195	0.004462258	0.002078113	0.013475944	470
AT5G61840	0.001560642	0.006042188	0.012423719	471
AT5G41600	0.006103779	0.013948912	0	472
AT5G53470	0.001715905	0.01029035	0.008048758	473
AT5G38460	0.004888423	0.001806264	0.013369935	474
AT4G17895	0.004213242	0.001851	0.014034508	475
AT3G23590	0.002813265	0.011121529	0.006177176	476
AT5G12100	0.001196292	0.008183258	0.010751076	477
AT2G27330	0.006327925	0.00959902	0.00421172	478
AT1G48170	0.005818979	0.000218903	0.01410343	479
AT2G17510	0.002844736	0.003036462	0.014285815	480
AT5G01290	0.00121498	0.004963845	0.013992543	481
AT5G13850	0.004836821	0.002335695	0.013011504	482
AT2G38950	0.001888893	0.00213492	0.016162298	483
AT1G73150	0.009981009	0.006069436	0.004140653	484
AT4G07390	0.001756195	0.002631078	0.015818026	485
AT5G16760	0.003317915	0.001725099	0.015163623	486
AT4G32930	0.006153643	0	0.014083791	487
AT3G15260	0.00397665	0.00447113	0.01179115	488
AT2G01970	0.001886112	0.001756229	0.016597825	489
AT5G06600	0.001733818	0.001594107	0.016944661	490
AT4G00650	0	0.00521489	0.015073471	491
AT5G53180	0.001629699	0.002991682	0.015678892	492
AT1G21380	0.00300516	0.00894785	0.008372525	493
AT1G50120	0.002066727	0.00446996	0.013823986	494
AT1G14830	0.00175102	0.005801828	0.012835713	495
AT4G02590	0.003607662	0.001923955	0.014908324	496
AT3G55380	0.007978651	0.003076686	0.009390386	497
AT3G05530	0.001097472	0.002643489	0.016706206	498
AT1G65430	0.000730869	0.00748485	0.012240327	499
AT5G49540	0.004748106	0.005650422	0.010065532	500
AT3G53440	0.010646079	0	0.009821344	501

AT5G51340	0.000237257	0.003908949	0.016325258	502
AT2G26660	0.005492264	0.009128715	0.005866955	503
AT4G17010	0.002984067	0.003723787	0.013788562	504
AT1G09020	0.001048203	0.002390005	0.01706033	505
AT4G35140	0.006606858	0.007873036	0.006019713	506
AT4G24880	0.009884253	0	0.010652952	507
AT4G16650	0.002091343	0.002529524	0.015925275	508
AT5G20580	1.60E-17	0.013618485	0.006959797	509
AT1G49950	0.002145452	0.00779022	0.01066333	510
AT4G22740	0.00362136	0.005086666	0.011901556	511
AT1G48900	0.000697392	0.001892536	0.018050504	512
AT4G27680	0.003407559	0.007547032	0.009696944	513
AT3G19980	0.00300204	0.000185346	0.017465922	514
AT4G31980	0.003679048	0.000913289	0.016064601	515
AT1G22450	0.001914307	0.004234024	0.014523864	516
AT5G20170	0.001818386	0	0.018897385	517
AT1G30450	0.001468282	0.001971511	0.017304458	518
AT2G44520	0.002339257	0.004647631	0.013764615	519
AT2G29190	0.003748399	0.005034535	0.011983037	520
AT5G24710	0.003098851	0.009505244	0.00817424	521
AT5G46620	0.008811793	0.006495061	0.005478237	522
AT5G03415	0.002487381	0.003181659	0.015126729	523
AT5G44200	0.001954328	0.003552926	0.015296563	524
AT5G61060	0.000916681	0.005799539	0.01409644	525
AT1G61730	0.006810546	0.000964196	0.013061446	526
AT1G79810	0.003849613	0.00275282	0.014240001	527
AT4G39920	0	0.005072093	0.015777461	528
AT5G50960	0.001722541	0.006093736	0.0130349	529
AT5G58440	0.003926891	0.008545261	0.008387719	530
AT5G66730	0.00192651	0.006921173	0.012021993	531
AT2G45640	0.006325147	0.000493376	0.01409727	532
AT5G48965	0.002717787	0.001705714	0.01650104	533
AT3G26990	0.004141924	0	0.016806361	534
AT5G04670	0.000735309	0.005659543	0.014566704	535
AT3G43240	0.001485009	0.002134064	0.017352015	536
AT5G41150	0.005306628	0.005304444	0.010379116	537
AT3G05040	0.003692851	0.004280126	0.01302418	538
AT4G32010	0.001528	0.00227785	0.017195106	539
AT4G25610	0.00403081	0.004794121	0.012186344	540
AT1G72550	0.002025125	0.001533254	0.017478757	541
AT3G63460	0.002723681	0.00269705	0.015616754	542
AT5G21990	0.001544113	0.008422147	0.011073331	543

AT2G43420	0.003895736	0.004202503	0.012951065	544
AT1G54490	0.002797585	0.003738725	0.014519929	545
AT1G73930	0.00185254	0.0017759	0.017488312	546
AT2G39170	0.002776371	0.006627041	0.011737507	547
AT3G17205	0.001856311	0.001321894	0.017977199	548
AT5G19400	0.002039831	0.006150966	0.012964908	549
AT1G80790	0.005864936	0.002576563	0.01273819	550
AT3G10730	0.003529712	0.001493576	0.016159256	551
AT2G33320	0.000927495	0.004943423	0.01532057	552
AT4G10760	0.000121749	0.005257437	0.01582558	553
AT1G11930	0.00646041	0.006957856	0.007822039	554
AT3G60820	0.002469677	0	0.018846399	555
AT4G25210	0.00150238	0.003850655	0.015982738	556
AT2G28480	0.001813133	0.004422874	0.015100343	557
AT1G14570	0.001745093	0.012437666	0.007161954	558
AT1G66750	0.00167342	0.010256105	0.009443804	559
AT1G27850	0.004079189	0.00061999	0.016693932	560
AT2G04620	0.001255614	0.005268088	0.014883967	561
AT3G10540	0.000155541	0.005163764	0.016114335	562
AT4G31440	0.005940395	0	0.015500976	563
AT4G01320	0.001424317	0.001901946	0.018116019	564
AT5G43320	0.000479496	0.004819751	0.016149688	565
AT5G05610	0.00098445	0.002029341	0.018441763	566
AT3G18990	0.001510286	0.002954102	0.017014728	567
AT1G09870	0.003603503	0.004432449	0.013461228	568
AT3G48150	0.005511674	0.001840027	0.014154551	569
AT3G61710	0.000860031	0.004738013	0.015913948	570
AT3G07330	0.000822871	0.002163687	0.018543706	571
AT2G41350	0.0020227	0.003998979	0.015514606	572
AT3G62140	0.013318019	0	0.008226704	573
AT2G25650	0.002410725	0.002607232	0.016529113	574
AT1G04750	0.003980599	0.003354195	0.014240239	575
AT5G10790	0.002906201	0.00056089	0.018116688	576
AT4G02010	0.003778872	0.005507	0.012322426	577
AT4G02640	0.005423029	0.004344647	0.011851694	578
AT5G59840	0.003790292	0.006207889	0.011627841	579
AT5G19350	0.003542166	0.003402851	0.014701972	580
AT5G40580	0.002617294	0.002524647	0.016569748	581
AT5G08750	0.005471633	0.015328889	0.000959821	582
AT4G14965	0.00365486	0.001415228	0.016717124	583
AT3G56150	0.00262716	0.002107668	0.017062111	584
AT3G14075	0.003355093	0.004789186	0.013666014	585

AT1G50370	0.004201844	9.12E-05	0.017526761	586
AT1G14610	0.00618878	0.001282903	0.014364659	587
AT3G03720	0.001361667	0.00758089	0.012918254	588
AT5G11910	0.007672569	0.004258682	0.009940529	589
AT1G25350	0.000822208	0.004611504	0.016453029	590
AT3G62120	0.00082858	0.00508703	0.015993248	591
AT2G19950	0.002173362	0.005519367	0.014237655	592
AT1G67960	0.004515754	0	0.017417701	593
AT4G34040	0.001802219	0.004310994	0.015834427	594
AT3G01435	0.005391907	0.003382765	0.013184747	595
AT3G58560	0.001633661	0.002922994	0.017408698	596
AT3G03790	0.003204533	0.005157742	0.013608344	597
AT4G00090	0.003516968	0.007348345	0.011120064	598
AT3G21290	0.006542703	0.000559224	0.014921595	599
AT3G05700	0.004926258	0.003762758	0.013381154	600
AT3G58580	0.00202669	0	0.020060847	601
AT3G10480	0.00456972	0.00863248	0.008894036	602
AT2G47350	0.002740519	0.004980408	0.014402624	603
AT2G01350	0.001191724	0.005646347	0.015302084	604
AT5G27740	0.00108614	0.002932218	0.018122274	605
AT1G53530	0.015317329	0.006837174	0	606
AT5G11640	0.000437409	0.011024216	0.010724362	607
AT5G12430	0.004255799	0.001447135	0.016483949	608
AT1G25490	0.003618065	0.003079267	0.0155136	609
AT3G50860	0.004897046	0.001641756	0.015684407	610
AT3G07950	0.001619945	0.003714319	0.01690805	611
AT3G05710	0.003088114	0.004310863	0.014846225	612
AT1G51450	0.001368655	0.013010046	0.007870909	613
AT3G16940	0.003308533	0.008827257	0.010117197	614
AT1G67890	0.003080709	0.002936015	0.016237755	615
AT3G02600	0.002442057	0.001195473	0.01863085	616
AT3G10210	0.002376435	0.008377005	0.011516024	617
AT4G32390	0.004556952	0.004157268	0.013555691	618
AT5G13050	0.003792203	0.000756198	0.017789312	619
AT1G33490	0.00108696	0.02125654	0	620
AT5G26760	0	0.003848213	0.018498427	621
AT1G68310	0.002925232	0.004734364	0.014740969	622
AT5G20165	0.004283529	0.00387188	0.014246776	623
AT1G43190	0.000887663	0.004957138	0.016581036	624
AT5G60620	0.002126749	0.005392265	0.014945876	625
AT5G38380	0.003344014	0.010193208	0.008965246	626
AT1G09330	0.004867113	0.001967853	0.01569217	627

AT1G68220	0.007205269	0.000746386	0.014576015	628
AT1G80070	0.006153655	0.001954317	0.014425831	629
AT1G71820	0.001074849	0.002040121	0.019439935	630
AT1G73730	0.006245724	0.003704687	0.012605797	631
AT3G56740	0	0.004471805	0.018093821	632
AT3G09090	0.000641851	0.001577461	0.020348783	633
AT2G27600	0.001528928	0.002616045	0.018442504	634
AT5G19485	0.00269443	0.011675094	0.008230384	635
AT1G67350	0.004587864	0.000634365	0.017403341	636
AT2G22740	0.002053342	0.004510515	0.016075774	637
AT3G25040	0.005162961	0.00312945	0.01435142	638
AT3G10915	0.003384733	0.002986446	0.016272964	639
AT1G63430	0.000804134	0.004509064	0.017333228	640
AT2G26780	0.003600496	0.000762089	0.018289476	641
AT5G61970	0.001654959	0.002039891	0.018965333	642
AT5G35730	0.0042412	0.00659831	0.011833944	643
AT5G22120	0.003370605	0.001097683	0.018223654	644
AT1G01710	0.00229564	0.006206235	0.014190823	645
AT5G05570	0.002857011	0.006791359	0.013048146	646
AT2G23940	0.007860788	0.003550564	0.011361509	647
AT5G10780	0.00513548	0.000716963	0.016931277	648
AT1G10290	0.002306861	0.008600083	0.011880902	649
AT4G29790	0.006182619	0.003019208	0.013623128	650
AT3G47060	0.003494628	0.003359085	0.015990956	651
AT5G49950	0.001964767	0.009222215	0.011678395	652
AT4G27120	0.004344431	0.003026904	0.015502131	653
AT1G49300	0.005104446	0.007635353	0.010147176	654
AT3G27520	0.004186901	0.014255963	0.004451572	655
AT5G22280	0.003435555	0.005319202	0.014140037	656
AT5G35840	0.003351313	0.008183352	0.011403737	657
AT2G26970	0.00687353	0.002106954	0.013988449	658
AT2G25670	0.001762397	0.005833429	0.015377474	659
AT5G05980	0.004125782	0.007647535	0.011213017	660
AT1G60995	0.00279244	0	0.020194186	661
AT4G11860	0.001011211	0.002731297	0.01924534	662
AT3G22220	0.002165755	0.002195375	0.018634929	663
AT1G21370	0.006086819	0.00060149	0.016311047	664
AT1G05120	0.003359198	0.003260799	0.016396948	665
AT2G02148	0.002075233	0.006134426	0.014817333	666
AT3G17880	0.000883733	0.00170271	0.020459509	667
AT5G24450	0.001167715	0.002553315	0.019357182	668
AT1G50430	0.002328362	0.004481151	0.016277075	669

AT1G27690	2.31E-17	0.005554784	0.017570516	670
AT5G25360	0.004584145	0.007301883	0.01126865	671
AT5G02310	0.005576756	0.003270214	0.014310774	672
AT4G35220	0.001898121	0.011041694	0.010232771	673
AT3G44530	0.003121162	0.000954486	0.0191134	674
AT5G25510	0.00093589	0.004869885	0.01739133	675
AT5G65290	0.001672785	0.00242588	0.01909848	676
AT1G74900	0	0.006832359	0.016378952	677
AT4G30600	0.001409299	0.007316521	0.014487275	678
AT5G46630	0.000562393	0.007497219	0.015155702	679
AT1G60670	0.004454471	0.000720845	0.018043612	680
AT1G75840	0.002238995	0.005003189	0.015988116	681
AT2G19430	0.005375885	0.001932107	0.01592899	682
AT4G25520	0.005030778	0.009461809	0.008775367	683
AT5G42620	0.002309943	0.008144335	0.012836256	684
AT2G26100	0.006986412	0	0.016304311	685
AT1G17110	0.003093445	0.002344157	0.017901887	686
AT4G00550	0.001640909	0.006503401	0.015195254	687
AT5G11040	0.003829592	0.008149704	0.011366754	688
AT1G80500	0.001989679	0.008902898	0.012457913	689
AT4G19003	0.004937648	3.32E-05	0.018380522	690
AT1G44960	0.020408963	0.002994587	0	691
AT1G08350	0.001780501	0.005045142	0.016577982	692
AT3G18370	0.002638108	0.009651432	0.011126443	693
AT5G12350	0.003887701	0.000601108	0.018942638	694
AT1G33270	0.003638555	0.000431731	0.019372216	695
AT1G19480	0	0.006972765	0.016470659	696
AT1G20696	0.002714011	0.004114578	0.016669486	697
AT2G46060	0.001576079	0.005330952	0.016599864	698
AT2G15860	0.003063809	0.001979941	0.018482939	699
AT1G34020	0.00206131	0	0.021469135	700
AT4G23040	0.001170866	0.003073872	0.019294585	701
AT3G07890	0.001573647	0.005729047	0.016237746	702
AT2G04880	0.001028785	0.005451767	0.017062956	703
AT1G69330	0.003409628	0.00525314	0.014906577	704
AT1G20960	0.004721843	0.002078206	0.01678282	705
AT3G04080	0.003687536	0.007380881	0.01252652	706
AT3G51610	0.005118209	0.0020366	0.016445579	707
AT3G26360	0.010937211	0	0.012664084	708
AT4G26310	0.005587049	0.003355429	0.014660603	709
AT4G38600	0.00619489	0.000948567	0.016460392	710
AT2G31820	0.003320751	0.006341686	0.013962964	711

AT3G01460	0.007509186	0.002158605	0.013997756	712
AT3G15410	0.003071442	0.007787504	0.012807624	713
AT2G26000	0.000937724	0.002320556	0.020413341	714
AT5G07370	0.002347907	0.005554962	0.015783537	715
AT5G03330	0.006120379	0.005405018	0.012187799	716
AT2G31140	0.006171472	0.004071651	0.013470924	717
AT5G20590	0.002507808	0.010864655	0.010348931	718
AT5G51510	0.003834352	0.001330714	0.018559343	719
AT2G39260	0.005028856	0	0.018750843	720
AT4G01650	0.002838951	0.005387094	0.015564462	721
AT3G20620	0.001028059	0.007471851	0.015322141	722
AT1G19430	0.004041903	0.004231013	0.015554971	723
AT4G38500	0.003220013	0	0.020647173	724
AT5G36230	0.003138766	0.007845742	0.012926621	725
AT1G71900	0.00233252	0.000926154	0.020685579	726
AT4G19006	0.002582564	0.003968014	0.017407509	727
AT2G40600	0.002598844	0.006630119	0.014735456	728
AT3G19640	0.004261157	0.000676025	0.019037087	729
AT2G31660	0.001013025	0.009236039	0.013731142	730
AT3G21430	0.005494865	0.000726979	0.017766473	731
AT2G44440	0.005131543	0.004767058	0.014100623	732
AT5G44150	0.005875394	0.002603706	0.015538929	733
AT2G36485	0.00163972	0.010105204	0.01228065	734
AT5G08510	0.011728754	0.012314508	0	735
AT5G24260	0.000907593	0.009415204	0.013722558	736
AT1G49040	0.003220908	0.002131481	0.018694518	737
AT5G26990	0.004620566	0	0.01943104	738
AT5G59160	0.002408255	0.00513625	0.016511542	739
AT5G57950	0.00239633	0.003678984	0.018020648	740
AT5G09260	0.005894459	0.014732431	0.003473784	741
AT4G28470	0.001594434	0.000751788	0.02175487	742
AT1G29990	0.003178414	0.003893675	0.01703572	743
AT4G06634	0.002171196	0.002928064	0.019018479	744
AT5G04920	0.000190787	0.00331267	0.020637021	745
AT4G06599	0.001277487	0.001402773	0.021460873	746
AT5G48520	0.003402578	0.00177911	0.018976613	747
AT5G46210	0.001834693	0.00074097	0.021606599	748
AT2G45690	0.00236226	0.005378683	0.016464671	749
AT3G05000	0.007056511	0.002208567	0.014957373	750
AT5G23630	0.002191684	0.002355152	0.019700689	751
AT3G16230	0.001623427	0.005896234	0.016735093	752
AT1G73100	0.004324272	0.006606828	0.013372962	753

AT2G43770	0.003476504	0.009187698	0.011641727	754
AT5G47400	0.002786212	0.005386983	0.016135015	755
AT3G14080	0.003659281	0.005733106	0.014919102	756
AT5G10110	0.00724805	0.002171827	0.014913551	757
AT4G39690	0.002981218	0.002205056	0.019147984	758
AT3G06910	0.000411554	0.009836405	0.014091671	759
AT5G65260	0.002861041	0.001407045	0.020114522	760
AT4G11410	0.002325936	0.006030607	0.016047728	761
AT4G36960	0.000575019	0.005370116	0.018468152	762
AT3G59600	0.003018324	0.003069651	0.018332253	763
AT2G45060	0.002509107	0.003522584	0.018469648	764
AT1G55830	0.003946118	0	0.020583669	765
AT4G34450	0.001723242	0.005395632	0.017420442	766
AT1G30090	0.010261668	0	0.014292267	767
AT3G07680	0.001649215	0.005895942	0.017015183	768
AT4G22220	0.002332636	0.001707075	0.020541207	769
AT5G38840	0.007448768	0.001258305	0.015887493	770
AT3G25540	0.001229393	0.006149907	0.017224341	771
AT5G23490	0.003596792	0.005862465	0.015176431	772
AT2G20190	0.003568408	0.002135542	0.018941866	773
AT3G11880	0.00187288	0.002379884	0.020426849	774
AT5G04430	0.002387249	0.005048772	0.017249139	775
AT1G48160	0.004405854	0.00186977	0.018410625	776
AT1G62020	0.003993974	0.007489262	0.0132046	777
AT1G51690	0.001245494	0.006986786	0.016501092	778
AT5G20930	0.00028453	0.003641361	0.020808529	779
AT1G60070	0.001411907	0.003631843	0.019705843	780
AT4G26630	0.009172018	0.003937334	0.011661383	781
AT5G12850	0.004095316	0.006501938	0.014175589	782
AT3G14800	0.004059569	0.009664479	0.01111552	783
AT5G05780	0.003345208	0.005464899	0.016030018	784
AT4G36140	0.005020556	0.006060774	0.013769031	785
AT4G00340	0.005494721	0.0109097	0.008462825	786
AT2G42670	0.000155084	0.00583205	0.018900235	787
AT4G11440	0.002012904	0.01493914	0.007935358	788
AT4G14340	0.001704745	0.009881502	0.013324602	789
AT1G30010	0.004308438	0.002227033	0.018388917	790
AT5G40520	0.001151405	0.012148525	0.011631378	791
AT1G45000	0.001408321	0.002683014	0.020841198	792
AT2G36740	0.004399262	0.001940012	0.018602479	793
AT3G17750	0.00095929	0.005033282	0.018969457	794
AT4G30160	0.004386327	0.006448187	0.014131533	795

AT1G78790	0.006526835	0	0.018454975	796
AT5G40200	0.00094698	0.008027075	0.016012915	797
AT1G57870	0.000700537	0.003175393	0.021121107	798
AT2G21470	0.001181035	0.001654197	0.02219285	799
AT1G27430	0.003768386	0.004223022	0.01705859	800
AT5G53620	0.004073394	0.002587439	0.01839192	801
AT4G26240	0.007165763	0.006270507	0.011625394	802
AT1G53000	0.004552709	0.006491715	0.014025624	803
AT1G04860	0.001565201	0.000870783	0.022637045	804
AT3G49310	0.001573496	0.011367633	0.012134038	805
AT4G23910	0.003089057	0.003944505	0.018073809	806
AT3G04780	0.007162305	0.002214963	0.01575589	807
AT2G40950	0.001676166	0.00780842	0.015667301	808
AT5G21274	0.010111184	0	0.015057816	809
AT2G41520	0.003711753	0.009336351	0.012140232	810
AT2G15560	0.001352657	0.000598638	0.023282501	811
AT1G80910	0.000301211	0.003020069	0.021925428	812
AT5G22030	0.002126143	0.007209405	0.015917029	813
AT5G51350	0.003316675	0.010716171	0.011226628	814
AT5G22330	0.00512683	0.005161059	0.014988824	815
AT1G76900	0.000593224	0.004035604	0.020660314	816
AT3G49850	0.002992277	0.005525517	0.016830232	817
AT5G17020	0.002977881	0.004420023	0.017960501	818
AT2G47760	0.002164962	0.005906325	0.017296287	819
AT1G17760	0.004374951	0.007003353	0.013993248	820
AT3G51050	0.002910832	0.001766861	0.020729243	821
AT3G07140	0.00137732	0.005896234	0.018142024	822
AT1G31730	0.001010186	0.003969055	0.020438074	823
AT4G13350	0.002089061	0.003642428	0.019697444	824
AT3G01040	0.000765633	0.007823397	0.016863194	825
AT3G04590	0.003226978	0.003774088	0.018456489	826
AT3G44600	3.45E-05	0.006348342	0.019091893	827
AT4G08690	0.002629834	0.007427734	0.01544551	828
AT4G37120	0.005009007	0.002725803	0.017776813	829
AT2G18960	0.002588505	0.01179354	0.011142032	830
AT5G39250	0.007063084	0.00663513	0.011864176	831
AT1G18560	0.022501959	0.001093953	0.002008932	832
AT4G02680	0.001512699	0.001893053	0.022201278	833
AT4G32240	0.007347532	0.007178023	0.011092345	834
AT5G65560	0.002979895	0.011888249	0.010764752	835
AT4G04970	0.002541243	0.006557965	0.016570218	836
AT5G47310	0.003630357	0.006740546	0.015306975	837

AT1G77440	0.002756502	0.006337174	0.016604089	838
AT2G07050	0.001160994	0.006367867	0.018173285	839
AT3G18350	0.000820047	0.01041833	0.014479747	840
AT3G09360	0.005678793	0.001775337	0.018271382	841
AT3G56760	0.00130043	0.011965496	0.012490033	842
AT3G05830	0.012800109	0.002092702	0.010864162	843
AT3G24820	0.011783017	0	0.013994183	844
AT3G07930	0.002434282	0.003990769	0.019371377	845
AT1G30540	0.002017751	0.003628143	0.020150803	846
AT5G49820	0.001898765	0.009285168	0.014615538	847
AT3G24040	0.000466726	0.005874314	0.01946476	848
AT3G20020	0.00090212	0.006159364	0.018781019	849
AT5G06910	0.005880019	0.006804031	0.013162792	850
AT3G14000	0.001924102	0.022395707	0.00155606	851
AT5G18410	0.003432682	0.004224727	0.018225428	852
AT4G38520	0.002406381	0.011840335	0.011647496	853
AT3G53930	0	0.007368494	0.018539338	854
AT1G24267	0.000616621	0.005660478	0.019634003	855
AT5G19280	0.001353601	0.005472431	0.019131341	856
AT5G64600	0.00324733	0.011535417	0.011188176	857
AT5G58230	0.001068415	0.006456588	0.018462966	858
AT2G15270	0.010392962	0	0.015596621	859
AT4G23460	0.002566246	0.007144165	0.016283531	860
AT1G73950	0.002564488	0.007504129	0.015944137	861
AT5G13020	0.000280047	0.00299616	0.022759395	862
AT2G31370	0.002160788	0.004100509	0.019775424	863
AT3G02780	0.004039933	0.009291971	0.012709478	864
AT5G10540	0.001134233	0.013797312	0.011112384	865
AT3G22630	0.004357983	0.00107176	0.020615703	866
AT4G09640	0.003201403	0.010946864	0.01190498	867
AT1G51350	0.003380138	0.006580628	0.016107894	868
AT1G17370	0.002999281	0.001609048	0.02150157	869
AT3G10070	0.002445221	0.01359152	0.010081154	870
AT1G08750	0.003843132	0.006728654	0.015549531	871
AT1G30910	0.003895571	0.008557852	0.013702532	872
AT1G09980	0.002964724	2.11E-05	0.023184471	873
AT3G15160	0.002558692	0.006607652	0.01700588	874
AT1G10670	0.00295468	0.002179932	0.021041511	875
AT5G50860	7.50E-05	0.011187687	0.014922273	876
AT1G31810	0.008120399	0.007749765	0.010345273	877
AT4G00730	0.002166587	0.014032826	0.010021456	878
AT2G27210	0.003931592	0.002351887	0.019959561	879

AT3G61540	0.002119494	0.004772035	0.01938048	880
AT2G39740	0.00334453	0.009641084	0.013295847	881
AT4G01290	0.002847007	0.004707086	0.018755195	882
AT5G54140	0.00373227	0.003240335	0.0194014	883
AT5G07970	0.00351182	0.002921484	0.019951303	884
AT5G56460	0.005525888	0.001676901	0.019183748	885
AT4G15415	0.001193502	0.002372688	0.022873888	886
AT5G64960	0.002265565	0.006988382	0.01719186	887
AT4G32330	0.003860311	0.007802437	0.014792121	888
AT5G08450	0.00523796	0	0.021224706	889
AT1G10350	0.012467309	0	0.014035763	890
AT1G10820	0.000693696	0.003164452	0.02265436	891
AT2G18860	0.005264329	0.015899347	0.005368016	892
AT1G78870	0.005291776	0	0.021240611	893
AT2G23520	0.00214612	0.004907204	0.019484522	894
AT4G29440	0.005803762	0.011615508	0.009135323	895
AT5G35980	0.002161604	0.002164527	0.022232092	896
AT4G26965	0.016100888	0	0.010493477	897
AT2G22370	0.011149237	0.000726702	0.014744872	898
AT3G04240	0.003081432	0.002425914	0.021123133	899
AT1G72390	0.005384142	0.001156382	0.020093789	900
AT1G72340	0.001400015	0.007391591	0.017858914	901
AT1G48635	0.002527551	0.009347197	0.014805733	902
AT2G27760	0.001957309	0.003542974	0.02119374	903
AT4G34110	0.002385347	0.007236107	0.017120964	904
AT2G26460	0.005576183	0.001177195	0.019994474	905
AT5G15400	0.001919506	0.004116266	0.020719006	906
AT2G01820	0.002562805	0.007203585	0.016994465	907
AT5G36290	0.002830369	0.00912224	0.014822701	908
AT2G34250	0.002914825	0.006002778	0.017860789	909
AT3G06550	0.00178846	0.013876779	0.011116675	910
AT2G38130	0.004657199	0.006017139	0.016134502	911
AT5G42220	0.004591818	0.000183548	0.022058816	912
AT3G27530	0.001387376	0.007591865	0.017867072	913
AT5G02530	0.001527639	0.005890005	0.019430385	914
AT5G62050	0.002531294	0.008073216	0.016245086	915
AT3G05010	0.002468562	0.001218905	0.023178128	916
AT5G42350	0.002867505	0.003845882	0.020156209	917
AT3G56460	0.001884469	0.0060693	0.018921375	918
AT1G24050	0.003671695	0.003452384	0.019783628	919
AT5G65670	0.0015798	0.010499171	0.014838038	920
AT3G46820	0.001136056	0.006638025	0.019153363	921

AT2G41790	0.002653193	0.004971503	0.019308911	922
AT2G39760	0.002270348	0.001579074	0.023101586	923
AT1G22920	0.002745467	0.000723418	0.023491015	924
AT3G15220	0.002224653	0.009790218	0.014972312	925
AT2G06990	0.0030102	0.003624213	0.020356522	926
AT3G58040	0.004252723	0.003540309	0.019199586	927
AT3G28430	0.002809469	0.005197721	0.019002185	928
AT3G04670	0.001818367	0.004164275	0.021031923	929
AT1G20575	0.008442976	0.001302126	0.017278535	930
AT2G36240	0.001146622	0.004344989	0.021553522	931
AT3G26560	0.002625624	0.00038731	0.02404999	932
AT4G03020	0.002137235	0.024944293	0	933
AT2G19470	0.00187633	0.006372994	0.018852875	934
AT1G51965	0.001634331	0.007347038	0.018125228	935
AT5G10870	0.003341421	0	0.023773624	936
AT5G43310	0.005268246	0.006907933	0.014945783	937
AT4G17890	0.001556399	0.009039111	0.016548033	938
AT1G04510	0.003007392	0.005092798	0.019091302	939
AT3G49100	0.010218728	0	0.016976206	940
AT3G18310	0.004560798	0	0.022641671	941
AT3G18140	0.002607343	0.000341125	0.024272545	942
AT1G11630	0.000333417	0.006939223	0.019953483	943
AT5G13530	0.004238051	0.00574419	0.017246392	944
AT5G18250	0.005086246	0.005605779	0.016545427	945
AT4G32272	0.018527075	5.66E-21	0.008739461	946
AT4G01280	0.00076476	0.007998618	0.018528304	947
AT3G27700	0.004263601	0.001382657	0.021646092	948
AT2G39840	0.001498064	0.007107904	0.018687841	949
AT3G54760	0.003025578	0.001278439	0.023011932	950
AT1G48230	0.001005058	0.004570658	0.021743867	951
AT5G01030	0.001908202	0.009700972	0.015714851	952
AT1G18950	0.005366585	0.001049321	0.020910903	953
AT5G40600	0.006567778	0.010306892	0.010467314	954
AT3G51310	0.001924918	0.002326991	0.023099137	955
AT3G47810	0.003512663	0.001313268	0.022540746	956
AT1G69670	0.001762274	0.001913265	0.023697019	957
AT3G62200	0.00596906	0.004156086	0.017276674	958
AT1G63290	0.001242723	0.003624136	0.022543461	959
AT1G76340	0.004523101	0.003982659	0.018921676	960
AT5G47480	0.003506818	0.002446061	0.021502965	961
AT5G02470	0.006970939	0.005877996	0.014608194	962
AT3G09230	0.006257938	0.001726679	0.019472753	963

AT4G26130	0.003431823	0.0107223	0.013348683	964
AT3G33530	0.00364288	0.003233861	0.020631879	965
AT1G18480	0.002418972	0.015350054	0.009745526	966
AT3G52120	0.001528853	0.003399197	0.022617301	967
AT5G51840	0.006199088	0.005941292	0.01543405	968
AT5G48385	0.001304785	0.002243821	0.024029996	969
AT3G27460	0.009627721	0	0.017961178	970
AT3G11590	0.002533747	0.013160789	0.011899485	971
AT3G59380	0.00158849	0.004931389	0.021082706	972
AT1G64990	0.00305566	0.002635071	0.021913799	973
AT1G17070	0.003365865	0	0.0242414	974
AT3G12210	0.007496792	0.004345128	0.015779804	975
AT4G23660	0	0.001345835	0.026277922	976
AT5G22220	0.002484952	0.003992575	0.021155927	977
AT1G59820	0.006022924	0.002330072	0.019283656	978
AT5G49930	0.001559393	0.002582483	0.023512142	979
AT1G47560	0.000855189	0.007036118	0.019771303	980
AT5G48020	0.002125634	0.004647209	0.0209139	981
AT4G29560	0.004413735	0.011183151	0.012090733	982
AT4G30000	0.0014247	0.017749341	0.008530031	983
AT4G38040	0.002854922	0.004913032	0.019940371	984
AT1G79090	0.001425325	0.002050905	0.024248459	985
AT4G19900	0.002889827	0.017110637	0.007745649	986
AT5G42470	0.003063012	0.006193992	0.018518508	987
AT3G59990	0.0007181	0.001153896	0.02591001	988
AT1G13020	0.001639516	0.002773075	0.02340408	989
AT5G52200	0.007664481	0.007555024	0.012611631	990
AT1G30300	0.008215575	0.004724943	0.014897719	991
AT3G15430	0.001295285	0.007390194	0.019184748	992
AT5G66810	0.00169455	0.002799948	0.023377592	993
AT5G10350	0.004246693	0.007155717	0.016473131	994
AT2G13650	0.005236399	0.009532557	0.01310968	995
AT5G51300	0.002728711	0.00589863	0.019302284	996
AT3G05850	0.005228888	9.65E-05	0.022604637	997
AT5G13030	0.0007672	0.005991053	0.02117963	998
AT2G40730	0.00118826	0.003650358	0.023103271	999
AT5G18620	0.003655498	0.005586005	0.01870706	1000
