# Supplementary material for: Identifying stably expressed genes from multiple RNA-Seq data sets
Source: PeerJ. 2016 Dec 20;4:e2791. doi: 10.7717/peerj.2791 (PMC5178351; doi:10.7717/peerj.2791)
Supplement: Table S3 [file peerj-04-2791-s003.pdf]

Supplemental Table S3: Top 1000 stably expressed genes identified from the leaves group.

Gene	between_sample	bewtween_treatment	between_experiment	Rank
AT3G28670	0.000557075	0.000972764	0.001105829	1
AT2G01220	0.003389582	0.001164988	0	2
AT3G21160	0.003708266	0	0.001469784	3
AT2G21150	0.002148003	0.00217146	0.001196078	4
AT1G47230	0.002312078	0.003758152	0.00024808	5
AT5G19680	0.004080695	0.001608482	0.000795573	6
AT1G25490	0.003362486	0.001024224	0.002113368	7
AT1G54650	0.001516592	0.00355497	0.002076455	8
AT1G66750	0.002750853	0.001026797	0.003416526	9
AT5G50020	0.001723926	0.004449122	0.001035392	10
AT5G12260	0.002563033	0.004194698	0.000457329	11
AT4G13730	0.004181913	0.002054174	0.000988544	12
AT3G02950	0.006481697	0	0.000761029	13
AT1G08750	0.003074846	0.004265742	0	14
AT5G19830	0.006732227	0	0.000641816	15
AT1G54610	0.004944058	0.002287438	0.000148912	16
AT2G44798	0	0.007621835	0	17
AT2G15270	0.002055228	0.00580119	0	18
AT1G50370	0.00271603	0.002226978	0.003000234	19
AT3G53110	0.002827094	0.000836678	0.004292892	20
AT3G03090	0.00056458	0.005783624	0.001672829	21
AT2G26590	0.002483485	0.002963246	0.002688007	22
AT5G08080	0.004746522	0.001330502	0.002213888	23
AT1G55620	0.002658803	0.005167913	0.000465627	24
AT4G17040	0.003217873	0.000319329	0.004766562	25
AT3G06910	0.001532657	0.005504422	0.001285083	26
AT2G19080	0.005039491	0.000513445	0.00299185	27
AT5G49060	0.002576841	0.005003161	0.00110307	28
AT4G38500	0.001813557	0.004029412	0.002923469	29
AT4G26965	0.0066228	0.001593361	0.000889218	30
AT1G78810	0.005154638	0.00263796	0.001329725	31
AT1G80000	0.005428474	0.001523866	0.002231965	32
AT1G75560	0.004554152	0.001615421	0.003197972	33
AT5G05010	0.002813125	0.002456822	0.004385155	34
AT5G41950	0.001717026	0.00357007	0.004412947	35
AT1G22940	0.002628671	0.005574441	0.001822963	36
AT5G18390	0.002679475	0.002287009	0.00512282	37
AT5G18420	0.004613332	0.00133537	0.004305144	38

AT5G11380	0.002136788	0.003666	0.004464175	39
AT1G71440	0.004453752	0.000844613	0.004983567	40
AT1G09020	0.003741044	0.00128041	0.005405354	41
AT5G08391	0	0	0.010487922	42
AT3G08550	0.005161444	0.004196308	0.001375189	43
AT1G54080	0.005140444	0.003055144	0.002595619	44
AT5G36210	0.005749171	0.005136333	0.000109822	45
AT1G51450	0.004937116	0	0.006279736	46
AT2G41350	0.004964869	0.003122812	0.003193929	47
AT5G14480	0.001975934	0.009481611	0	48
AT2G35530	0.002907104	0.006370542	0.002232271	49
AT5G41980	0.004583764	0.003142134	0.00382739	50
AT5G18900	0.004207981	0.007401546	0	51
AT4G26640	0.0037467	0.006740327	0.001254285	52
AT5G19130	0.005483718	0.002851621	0.003446489	53
AT3G03570	0.004375944	0.002320287	0.005091623	54
AT2G19160	0.004440978	0.002457749	0.005007914	55
AT3G07330	0.006181581	0.004942475	0.000816455	56
AT5G64470	0.005156898	0.006788538	0	57
AT5G06580	0.00076991	0.006981598	0.004572232	58
AT1G21280	0.008832601	0.003493879	0	59
AT1G16690	0.001706493	0.010708148	0	60
AT3G20920	0.002041831	0.004612767	0.005764218	61
AT1G29550	0.002801772	0.009637972	0	62
AT1G13060	0.008545139	0	0.003952293	63
AT5G60570	0.005484867	0.0017906	0.005240366	64
AT2G40980	0.006823154	0.002694706	0.003144354	65
AT5G44150	0.003803509	0.004718232	0.004156759	66
AT5G48520	0.002112806	0.001380338	0.009206411	67
AT1G52630	0.003130207	0.001138563	0.008579223	68
AT1G62930	0.002024714	0.010856377	0	69
AT3G59800	0.00530538	0.007598427	0	70
AT5G16890	0.002633455	0.001158522	0.009140185	71
AT1G34470	0.00257436	0.003491593	0.006961799	72
AT1G19170	0.001876628	0.009268641	0.001987786	73
AT1G11930	0.001400582	0.004955067	0.006784732	74
AT5G35730	0.004077941	0.003017462	0.006199187	75
AT2G30880	0.005208079	0.000901857	0.00720473	76
AT4G30900	0.00466422	0.00425723	0.004393731	77
AT1G55150	0.000418794	0.006080873	0.006864878	78
AT5G41870	0.00870047	0.004680456	0	79
AT5G67610	0.010725715	0.001407107	0.001324445	80

AT4G23330	0.003839106	0.007710121	0.001955472	81
AT3G15430	0.007724566	0.005796637	0	82
AT5G63440	0.007758986	0	0.005762643	83
AT2G17420	0.009026567	0.001878622	0.002656547	84
AT3G15660	0.004227007	0.003173667	0.006173875	85
AT1G65650	0.001932642	0.004254999	0.00745878	86
AT1G74800	0.006528025	0.006693945	0.000526249	87
AT2G20810	0.004337258	0.006803462	0.002770823	88
AT3G06820	0.000356863	0.00161886	0.011939602	89
AT2G30910	0.001765897	0.010533375	0.001745583	90
AT5G07120	0.00180534	0.011208395	0.001086104	91
AT5G18950	0.000506132	0.010989984	0.002744116	92
AT1G04850	0.005462327	0.008183341	0.000621696	93
AT5G30490	0.007357891	8.66E-05	0.006887085	94
AT4G33060	0.005618112	0.0087226	0	95
AT4G22150	0.006701946	0.000441183	0.007237187	96
AT1G28320	0.00496701	0.003563078	0.005898883	97
AT4G32120	0.001296823	0.005578485	0.007684569	98
AT3G07140	0.001960779	0.012664437	0	99
AT5G46020	0.005544751	0.00908803	3.52E-07	100
AT1G48175	0.005826958	0.003805162	0.005038936	101
AT5G40580	0.006631563	0.004492003	0.003557007	102
AT5G41860	0.005258338	0.008995301	0.000440492	103
AT4G16580	0.007000978	0.007738319	0	104
AT1G65020	0.002686772	0.00672294	0.005377725	105
AT3G63150	0.006594188	0.000482715	0.007751276	106
AT5G25510	0.005126532	0.00273407	0.007153337	107
AT4G09670	0.006491503	0.002881956	0.005649118	108
AT1G22200	0.000866039	0.005217711	0.009002665	109
AT4G37270	0.006177999	0.008923621	0	110
AT5G63110	0.005586665	0.002268144	0.007290251	111
AT1G13320	0.002597611	0.002287372	0.0103061	112
AT3G08990	0.007375516	0.003882772	0.00398538	113
AT5G17070	0.00463081	0.005267132	0.005365713	114
AT3G50860	0.008295475	0.006984777	0	115
AT5G57460	0.004302979	0.008158465	0.002873859	116
AT2G39750	0.003298963	0.005447751	0.006681619	117
AT3G22590	0.008384577	0.004560199	0.002510578	118
AT2G20830	0.00653549	0.008982594	0	119
AT5G55130	0.007126845	0.003034972	0.005370064	120
AT3G24200	0.001330284	0.009232425	0.004978399	121
AT1G76030	0.003366462	0.005376386	0.006811102	122

AT1G60780	0.004837008	0.005625334	0.005110137	123
AT3G52200	0.009991425	0.001851166	0.003783973	124
AT5G15570	0.000441083	0.007204949	0.008005875	125
AT3G42050	0.003482913	0.001519495	0.01066537	126
AT1G54390	0.004117245	0.00633677	0.005270646	127
AT2G37160	0.007583352	0.006788639	0.001407382	128
AT5G09900	0.005112273	0.000957822	0.00971691	129
AT1G77610	0.006586811	0.007900002	0.00130437	130
AT5G45620	0.003899194	0.004567445	0.007326071	131
AT5G58230	0.006228726	0.001670333	0.007925909	132
AT3G24490	0.005662408	0.006149603	0.00401398	133
AT5G19280	0.005747001	0.00452811	0.005576644	134
AT4G34270	0.004351413	0.000755261	0.010762153	135
AT1G80040	0.004669606	0.00955695	0.001726886	136
AT4G24820	0.004350129	0.0041406	0.007481319	137
AT3G18990	0.003696871	0.006043177	0.006272461	138
AT3G02420	0.004810761	0.011241334	0	139
AT4G23100	0.009300704	0.003539749	0.003223349	140
AT3G53970	0.005905873	0.01024719	0	141
AT3G01910	0.007679259	0.002312598	0.006198453	142
AT1G20200	0.00632416	0.002837755	0.007055185	143
AT5G18230	0.008126708	0.003837717	0.004266359	144
AT4G32560	0.012402442	5.73E-17	0.00383508	145
AT1G01910	0.001424685	0.006051699	0.00878818	146
AT1G08030	0.004731701	0.004688642	0.006912067	147
AT5G35590	0.005597141	0.000720707	0.010021012	148
AT5G48680	0.000868853	0.008331016	0.007154352	149
AT3G14920	0.009314363	0.006086285	0.000982568	150
AT5G06950	0.006097133	0.002187471	0.008113117	151
AT1G47330	0.004740382	0.011683777	0	152
AT5G02130	0.003148177	0.006052129	0.00734357	153
AT4G34840	0.005631058	0.007961366	0.002973393	154
AT2G43190	0.00557868	0.006042039	0.004999611	155
AT3G10730	0.004327356	0.001787647	0.010519622	156
AT3G52210	0.004270463	0.002684548	0.009684988	157
AT1G45000	0.003736636	0.001978372	0.010940564	158
AT5G14310	0.002146976	0.01454168	0	159
AT3G14290	0.007268538	0.005316928	0.004147376	160
AT5G13070	0.00726117	0.007190859	0.002286818	161
AT1G51590	0.004854804	0.002877412	0.00913082	162
AT3G20070	0.001217838	0.012774071	0.002882294	163
AT5G11760	0.007254763	0	0.009840909	164

AT1G51560	0.004349334	0.003958872	0.008898858	165
AT1G43190	0.003331392	0.010854892	0.003041501	166
AT1G25380	0.003566714	0.008315575	0.005358434	167
AT3G53520	0.001720727	0.00783573	0.007706841	168
AT5G47790	0.004854614	0.000642976	0.011805048	169
AT1G60670	0.004529141	0.010327066	0.00244789	170
AT5G57210	0.003732626	0.012629642	0.000961581	171
AT1G68370	0.003981834	0.004960029	0.008427445	172
AT1G79810	0.002572752	0.00609289	0.00877065	173
AT5G19485	0.004025327	0.002348513	0.011106962	174
AT1G19080	0.002157574	0.000536545	0.014812529	175
AT2G20360	0.004186182	0.006604193	0.00673319	176
AT5G63460	0.007386006	0.004520547	0.005638286	177
AT4G39370	0.00310818	0.004307745	0.010214868	178
AT5G45290	0.010291054	0.007354523	0	179
AT4G11790	0.00471071	0.007092934	0.005951145	180
AT1G19480	0.005224599	0.001636978	0.010929832	181
AT3G58560	0.002820618	0.006439351	0.008721881	182
AT5G11980	0.003465149	0.010153119	0.004393286	183
AT1G79030	0.005739495	0.002966591	0.009320207	184
AT5G17690	0.002860001	0.015176779	0	185
AT1G02090	0.011451202	0	0.006613405	186
AT4G24840	0.006804822	0.004898774	0.006384043	187
AT4G38510	0.011693288	0	0.006405648	188
AT4G15010	0.009102695	0	0.009002251	189
AT5G61580	0.005232108	0.003034715	0.009839438	190
AT1G55170	0.006482386	0.01167647	0	191
AT5G38470	0.003237817	0.007432629	0.007523851	192
AT3G51390	0.006902519	0.003407843	0.007966847	193
AT2G22370	0.009371477	0.008745766	0.000186883	194
AT5G58100	0.011291468	0.003247582	0.003811404	195
AT1G08780	0.006587391	0.003282248	0.008489864	196
AT4G11970	0.009948885	0.003432458	0.00506389	197
AT5G53850	0.004635005	0.005948974	0.007962092	198
AT3G12990	0.005308012	0.01059615	0.002649926	199
AT3G23660	0.00422341	0.010318611	0.004023314	200
AT4G26100	0.004079773	0.003571821	0.010944947	201
AT1G02130	0.006015892	0.003529146	0.009052208	202
AT2G39170	0.009366589	0.008170303	0.001066272	203
AT5G07300	0.008228541	0.006769212	0.003620966	204
AT1G64840	0.009276422	0.007823592	0.001561458	205
AT5G51230	0.005182683	0.002659307	0.010845908	206

AT3G55000	0.002595416	0.008008623	0.008100625	207
AT2G15530	0.00697923	0.007560912	0.004165462	208
AT1G26160	0.002949594	0.004791259	0.010979995	209
AT5G66100	0.012849467	0	0.005871516	210
AT4G24530	0.002954545	0.007085074	0.008754537	211
AT4G15415	0.009966839	0	0.008839442	212
AT1G60220	0.010968948	0.001009066	0.006846736	213
AT3G13360	0.004852429	0.013974307	0	214
AT4G28540	0.00275476	0.008393042	0.007726268	215
AT5G19180	0.007562516	0.003317676	0.007996327	216
AT2G42230	0.006330864	0.010606447	0.001961735	217
AT3G10330	0.002963173	0.014529249	0.00141797	218
AT1G23465	0.008635308	0	0.010285599	219
AT3G04500	0.000976967	0.010787722	0.007161873	220
AT1G53050	0.005011964	0.013959146	0	221
AT1G77130	0.013093436	0.005381552	0.000521906	222
AT2G04340	0.004100941	0.005389626	0.009532784	223
AT2G45690	0.006464782	0.006557882	0.006037717	224
AT1G11900	0.010296987	0.003597817	0.005186843	225
AT1G73930	0.005846586	0.010285122	0.002950045	226
AT5G19840	0.001625091	0.016189104	0.001272936	227
AT2G18410	0.001523021	0.0038964	0.013687607	228
AT4G17616	0.002923695	0.007146021	0.009047658	229
AT1G03040	0.004302712	0.011451651	0.003365698	230
AT3G12130	0.004494534	0.004965858	0.00975009	231
AT5G51700	0.003060706	0.008967167	0.007203017	232
AT1G50510	0.004996955	0.002359216	0.011922829	233
AT4G36890	0.003524352	0.015806829	0	234
AT2G23940	0.005885976	0.00422519	0.009232516	235
AT1G51710	0.006336088	0.006299604	0.006727781	236
AT3G60830	0.002939209	0.006416698	0.010054582	237
AT1G67930	0.005178235	0.013521609	0.00077094	238
AT1G33330	0.013121548	0.006382851	0	239
AT4G24490	0.003287703	0.0059754	0.01028796	240
AT5G42790	0.006669922	0.009605889	0.003283841	241
AT1G71350	0.006467864	0.013137626	0	242
AT3G13772	0.005789689	0.001477974	0.012449351	243
AT4G17420	0.002132267	0.00911147	0.008486988	244
AT1G17270	0.01264872	0.005427613	0.00169831	245
AT1G75990	0.014855004	0.000673379	0.004288572	246
AT1G52370	0.006046955	0.013779873	0	247
AT3G26370	0.00223109	0.01185871	0.005740683	248

AT5G59160	0.001675356	0.007222611	0.010941116	249
AT5G49830	0.003561087	0.003412757	0.012903061	250
AT2G05755	0.007123615	0	0.012754601	251
AT4G19006	0.004692657	0.007025108	0.00820973	252
AT3G27470	0.00557044	0.012219895	0.002169503	253
AT1G14020	0.004895624	0.00716763	0.007964182	254
AT1G08710	0.006199179	0.00772723	0.00612254	255
AT5G16510	0.006514679	0.005475784	0.008108217	256
AT1G49820	0.005142567	0.011293825	0.003667012	257
AT1G07960	0.006527812	0.002571389	0.011013	258
AT2G02730	0.005129703	0.004948816	0.010035114	259
AT4G23640	0.004343709	0.009599643	0.00621661	260
AT1G47420	0.005291223	0.003551231	0.011326231	261
AT3G02520	0.005399896	0.007493415	0.007282635	262
AT3G01435	0.004129194	0.00367715	0.01237259	263
AT4G32360	0.009002703	0.011182709	0	264
AT4G25550	0.020273874	0	0	265
AT2G41630	0.006655346	0.013619823	0	266
AT2G45260	0.005533371	0.004646246	0.01015187	267
AT1G29710	0	0.002486084	0.017858036	268
AT4G16100	0.006117757	0.01387756	0.000412122	269
AT5G14850	0.002394058	0.00661153	0.011409561	270
AT1G29810	0.005268189	0.015156315	0	271
AT1G78800	0.004485431	0.002180282	0.013764795	272
AT3G54190	0.006897475	0.003934367	0.009611933	273
AT2G03220	0.003353245	0.01056166	0.006530105	274
AT3G18380	0.009489156	0.00524848	0.005708222	275
AT2G32070	0.010709412	0.003331829	0.006423736	276
AT2G01620	0.004909023	0.009513379	0.00609254	277
AT5G40270	0.012583796	0.005552542	0.002381172	278
AT1G07980	0.006498747	0.00723835	0.006787214	279
AT2G17390	0.004288091	0.013133492	0.003158491	280
AT1G17470	0.003194452	0.000807358	0.016618966	281
AT5G43010	0.007935587	0.002047129	0.010658103	282
AT2G20495	0.007449963	0	0.013270378	283
AT3G22110	0.01181774	0.001767689	0.007146763	284
AT4G11640	0.00204645	0.014059573	0.004657723	285
AT3G03940	0.006436615	0.000929743	0.013449433	286
AT5G18580	0.001455804	0.006045503	0.0133342	287
AT1G09150	0.004974486	0.006935824	0.008937738	288
AT4G21420	0.016001027	0	0.004860114	289
AT4G36480	0.004710492	0.004513182	0.011733868	290

AT2G02148	0.0061752	0.008084103	0.006700484	291
AT1G52310	0.011182717	0.00250007	0.00728372	292
AT4G00650	0.005830773	0.009714915	0.005435321	293
AT5G26610	0.007878801	0.004247436	0.008878941	294
AT1G15490	0.007446658	0.010095161	0.003553257	295
AT4G11260	0.006459008	0.002617778	0.012039182	296
AT5G47620	0.006437708	0.014690169	0	297
AT3G17660	0.001063371	0.007824974	0.012269538	298
AT5G06130	0.014800691	0	0.006390826	299
AT5G50410	0.00583673	0.008369449	0.006992052	300
AT3G27310	0.004766125	0.005637608	0.01082141	301
AT5G42360	0.01013943	0.011099934	0	302
AT1G24180	0.006306687	0.00960184	0.005381994	303
AT2G15860	0.005822289	0.007669988	0.007819954	304
AT3G10480	0.006746463	0.009861124	0.004726181	305
AT1G05270	0.004124427	0.002844906	0.014426845	306
AT4G01320	0.00522393	0.016244566	0	307
AT3G56460	0.003616598	0.005047225	0.012822164	308
AT5G58270	0.007620023	0.006805975	0.007060135	309
AT3G19980	0.003431327	0.008523456	0.009546604	310
AT5G51180	0.007019167	0.0089886	0.005514196	311
AT4G29100	0.008394457	0.013171273	0	312
AT4G32390	0.009355102	0.005547085	0.006763393	313
AT3G27320	0.005505529	0.007109913	0.00907769	314
AT3G22845	0.007803291	0.013893614	0	315
AT3G44110	0.007644677	0.010994395	0.003074448	316
AT1G02145	0.009575785	0.012154706	0	317
AT4G24550	0.00177735	0.001409702	0.018556983	318
AT5G53800	0.003381118	0.009519077	0.008847906	319
AT1G30540	0.009711042	0.012054784	0	320
AT2G34410	0.00610138	0.00329578	0.012413918	321
AT1G70770	0.006329303	0.00733085	0.008167598	322
AT3G06540	0.005350078	0.001896348	0.014608555	323
AT2G17540	0.003261847	0.003144703	0.015457629	324
AT1G01350	0.007422833	0.006711128	0.007781039	325
AT1G68185	0.001026014	0.007918023	0.012976729	326
AT4G28470	0.005854148	0.007505408	0.008600529	327
AT1G48970	0.003850238	0.009365132	0.008749034	328
AT3G22470	0	0	0.02202592	329
AT2G42240	0.002192128	0.000405244	0.019447895	330
AT1G16210	0.013104614	0.002675158	0.00626836	331
AT3G16110	0.007956118	0.002923598	0.011232973	332

AT3G09180	0.00868545	0.008726883	0.004757439	333
AT1G72340	0.016203606	0.003896099	0.002095755	334
AT2G03050	0.018755019	0	0.003453926	335
AT2G28480	0	0.002132101	0.020079248	336
AT1G29150	0.005416354	0.005432154	0.011381037	337
AT1G51390	0.003092125	0.011592178	0.007553653	338
AT1G03190	0.001948682	0.012332521	0.007970634	339
AT5G38560	0.013341877	0.001540839	0.007397537	340
AT5G57950	0.005554732	0.016753951	0	341
AT4G30920	0.016639633	0.003252327	0.002442512	342
AT5G53180	0.000687738	0.00618172	0.015488566	343
AT3G59600	0.010323651	0.007462769	0.004600014	344
AT1G33290	0.009004801	0.003647846	0.009753177	345
AT5G11350	0.00666177	0.015214056	0.000578897	346
AT3G60410	0.004092168	0.012634097	0.005734422	347
AT4G06599	0.006866487	0.006440546	0.00917826	348
AT2G17530	0.006757712	0.014374279	0.001381097	349
AT3G02090	0.009587374	0.00070181	0.012284826	350
AT1G03330	0.008152798	0.010621038	0.003824585	351
AT3G62970	0.003546223	0.006525395	0.01253309	352
AT2G02380	0	0.001895927	0.020712863	353
AT5G51880	0.00996801	0.009224582	0.003418313	354
AT5G56450	0.014579816	0.004357809	0.003685092	355
AT3G58130	0.002951657	0.000196224	0.019501115	356
AT2G38660	0	0.022668191	0	357
AT2G32170	0.001227116	0.007428447	0.014053055	358
AT1G68100	0.007821384	0.010552838	0.004344829	359
AT4G25880	0.004838383	0.017932753	0	360
AT2G39940	0.010498517	0.012334786	0	361
AT1G50710	0.003442367	0.014193387	0.00523041	362
AT2G16950	0.007878429	0.004405991	0.0106091	363
AT4G19150	0.002385809	0.003007562	0.017501002	364
AT3G28690	0.018100024	0.002058283	0.002749411	365
AT1G63170	0.004521869	0.007800471	0.01058946	366
AT3G04080	0.003966766	0.01418739	0.004810433	367
AT5G08120	0.005332423	0.001302112	0.016339879	368
AT1G72310	0.005764587	0.006552133	0.010713282	369
AT1G10430	0.00456613	0.009957646	0.008527921	370
AT1G26110	0.009321725	0.002973902	0.010811768	371
AT2G31290	0.012982654	0.008433652	0.001698366	372
AT3G26935	0.003378911	0.003591195	0.016152923	373
AT2G23370	0.001585039	0.007427276	0.014142609	374

AT5G53470	0.005435658	0.004163765	0.013604874	375
AT3G56110	0.014104032	0	0.00910864	376
AT4G20310	0.00426653	0.012792658	0.006189974	377
AT1G74320	0.007006667	0.008816976	0.007433474	378
AT5G27710	0.004383744	0.002616626	0.016262446	379
AT5G59460	0.004211033	0.008520618	0.010589941	380
AT2G43070	0.009001369	0.000743205	0.013625897	381
AT3G45400	0.008517725	0.009003486	0.005899856	382
AT3G11290	0.00449989	0.007517977	0.011443424	383
AT5G20880	0.005352756	0.006851415	0.01126114	384
AT1G71800	0.004595214	0.005729468	0.013149915	385
AT4G35240	0.011839171	0.011682362	0	386
AT4G02730	0.00219179	0.008119399	0.013231121	387
AT2G20860	0.020292396	0.00328058	0	388
AT3G47940	0.01281613	0.010777756	9.18E-10	389
AT5G20570	0.01200651	0.010428319	0.001198489	390
AT3G58090	0.001337049	0.014966226	0.00733105	391
AT3G26920	0.018638178	0.001026663	0.003970782	392
AT1G74510	0.004080681	0.014393738	0.005175457	393
AT3G46180	0.013016658	0.008750618	0.001966269	394
AT1G65410	0.003941572	0.007805891	0.012000689	395
AT4G37020	0.003884017	0.006028788	0.013838709	396
AT5G13020	0.003553151	0.010594516	0.009612693	397
AT2G37680	0.000755965	0.013045175	0.01004603	398
AT5G42970	0.006604025	0.009377183	0.007876149	399
AT5G66090	0.017860505	0.001726398	0.004273278	400
AT5G40650	0.004463041	0.014048448	0.005362558	401
AT3G19870	0.003720409	0.011777271	0.008404956	402
AT4G04720	0.01192467	7.23E-05	0.011931047	403
AT3G59990	0.002473151	0.007038112	0.014427509	404
AT3G15160	0.007469756	0.013353293	0.003187214	405
AT1G80670	0.006284311	0	0.017731339	406
AT1G05940	0.005424734	0.004165119	0.014430314	407
AT2G32040	0.003062493	0.006240554	0.01475258	408
AT5G04420	0.005817014	0.006721368	0.011565877	409
AT4G26000	0.006154044	0.007859784	0.01009437	410
AT1G80230	0.008927152	0.01096666	0.0042316	411
AT1G74960	0.007119692	0.013208043	0.003825252	412
AT3G46200	0.006717856	0.017460855	0	413
AT1G15730	0.010649444	0.007396951	0.006141815	414
AT1G17760	0.003193109	0.013603672	0.007392108	415
AT3G02600	0.008875498	0.003145749	0.012174945	416

AT4G01100	0.003455726	0.008516764	0.012239134	417
AT1G04910	0.001737291	0.003099017	0.019396796	418
AT5G43600	0.006977135	0.007589066	0.009677048	419
AT2G35360	0.000165367	0.009665895	0.014455359	420
AT3G59920	0.002529466	0.003160339	0.018625566	421
AT2G26770	0.00695018	0.005012968	0.012410145	422
AT4G26570	0.003775739	0.00415496	0.01645261	423
AT1G70570	0.004259398	0.004658219	0.015512129	424
AT3G07170	0.000455264	0.008236487	0.015776918	425
AT3G52640	0.001953095	0.012727616	0.009867348	426
AT1G32610	0.011233729	0.004948949	0.008387411	427
AT5G08530	0.003744891	0.00365247	0.017205317	428
AT3G58530	0.004062922	0.011897863	0.00864255	429
AT2G27090	0.00844299	0.008806081	0.007359904	430
AT5G63910	0.005091425	0.007843442	0.011681318	431
AT3G03100	0.01436072	0.002824593	0.007458581	432
AT3G20740	0.00392531	0.013118245	0.007613011	433
AT4G34110	0.007524364	0.000385327	0.016823151	434
AT1G80700	0.004905439	0.004301962	0.015530413	435
AT2G45023	0.008594618	0.016153984	0	436
AT1G75340	0.008569828	0.009230255	0.006955115	437
AT5G14260	0.005262715	0.008734072	0.010773765	438
AT5G54430	0.005537528	0.00811015	0.011139071	439
AT1G79010	0.01182492	0.001387021	0.011577178	440
AT1G73430	0.011534259	0.009531504	0.003732233	441
AT5G58920	0.007659452	0.017142701	0	442
AT2G35100	0.002351181	0.007266491	0.015210059	443
AT2G38695	0.007302227	0.011530797	0.005999982	444
AT5G01430	0.008413511	0.010275206	0.006145385	445
AT4G21800	0.010668414	0.010555325	0.003633194	446
AT4G03000	0.005641626	0.010281213	0.008956429	447
AT2G27800	0.010949143	0.011462391	0.0025787	448
AT1G05180	0.001841078	0.001737748	0.021412081	449
AT1G73730	0.002788616	0.008673774	0.013534848	450
AT1G34320	0.009467641	0.00196578	0.013583279	451
AT5G58003	0.001760523	0.009896095	0.013420459	452
AT5G01310	0.001750287	0.005855163	0.017503093	453
AT1G29990	0.004336174	0.011119858	0.009658404	454
AT1G03000	0.010179706	0.011027223	0.003919969	455
AT3G05510	0.006966385	0.009152613	0.009026105	456
AT5G51340	0.009321775	0.002349194	0.013485664	457
AT4G35040	0.004190913	0.021010763	0	458

AT4G38220	0.010399069	0.009489552	0.005328631	459
AT3G52030	0.002821774	0.018434817	0.003964309	460
AT4G29660	0.005556608	0.016743208	0.002947889	461
AT1G02410	0.004325712	0.014123967	0.006810829	462
AT3G02555	0.008645362	0.001562607	0.015064175	463
AT5G43670	0.009627656	0.012220306	0.003442706	464
AT4G27650	0.0064449	0.012018765	0.006831037	465
AT1G54340	0.004653297	0.011067238	0.009599645	466
AT5G37850	0.004532355	0.018968336	0.001856491	467
AT4G39240	0.006988695	0.003872935	0.014582546	468
AT2G43980	0.004263068	0.010744157	0.010485748	469
AT5G05670	0.010384124	0.008520232	0.006624737	470
AT2G35610	0.007802657	0.017760458	0	471
AT1G28120	0.005869896	0.011608598	0.008253798	472
AT1G79690	0.012591024	0.007461775	0.005700015	473
AT5G28900	0.005860523	0.004126966	0.015767648	474
AT5G63520	0.005311171	0.005789832	0.014678871	475
AT5G65260	0.009184677	0	0.016597169	476
AT4G39820	0.007500481	0.014574894	0.003743182	477
AT1G11915	0.01321254	0.00356633	0.009049399	478
AT5G15460	0.013833449	0.001117547	0.010880801	479
AT5G19910	0.013427901	0.000231801	0.012176047	480
AT1G79020	0.001743738	0.015917141	0.008175669	481
AT4G22320	0.011459645	0.010775747	0.003607931	482
AT4G30480	0.003956895	0.000863128	0.021034677	483
AT3G07180	0.005743929	0.007865346	0.012245763	484
AT1G08000	0.011317589	0	0.014551379	485
AT1G19025	0.018637826	0	0.007232725	486
AT5G18190	0.009428921	0.002929163	0.013513353	487
AT3G04605	0.006119291	0.009574431	0.010208985	488
AT2G36740	0.01225666	0.010776416	0.002882833	489
AT5G54890	0.005178884	0.020772093	0	490
AT3G62830	0.004520728	0.017523379	0.003915626	491
AT4G38930	0.009140506	0.005881009	0.010942577	492
AT3G18210	0.003276358	0.004091277	0.018666882	493
AT5G67580	0.002868252	0.006364156	0.016805321	494
AT4G27120	0.0069541	0.004473903	0.014612291	495
AT1G56000	0.002045297	0.004412908	0.019593253	496
AT4G23910	0.001644134	0.016597097	0.007883038	497
AT1G61770	0.016135631	0.001202531	0.008795686	498
AT3G53570	0.002381471	0.006199539	0.017553256	499
AT4G14190	0.006945065	0.00593453	0.013255021	500

AT1G79990	0.007438531	0.011245132	0.00745697	501
AT5G53560	0.012638274	0.010092718	0.003412095	502
AT3G48820	0.005159214	0.015625777	0.005366305	503
AT4G24880	0.003770388	0.004232877	0.018157899	504
AT3G24010	0.008582668	0.005446938	0.012136128	505
AT4G30820	0	0.008433909	0.01773952	506
AT5G06660	0.00400982	0.005665904	0.016540544	507
AT5G58560	0.009795129	0.008805094	0.007639352	508
AT2G04620	0.008646889	0.005738521	0.011855901	509
AT3G18790	0.007547471	0.003771633	0.014936596	510
AT1G63270	0.013409997	0.0027262	0.010123303	511
AT1G52420	0.025433622	0.000609334	0.000243193	512
AT3G01360	0.006560796	0	0.019748059	513
AT5G41480	0.007126677	0.005299829	0.013911196	514
AT3G03305	0.011320622	0.005893681	0.009163258	515
AT5G32470	0.002988017	0.013718669	0.009714186	516
AT1G04985	0.006716439	0.010284399	0.009424088	517
AT1G66660	0.005243887	0.006928503	0.014258206	518
AT2G38630	0.007299032	0.019171575	0	519
AT2G48000	0.0032965	0.019314975	0.003902461	520
AT4G26410	0.005935347	0.008311393	0.01228044	521
AT1G06890	0.004290748	0.011175414	0.011074445	522
AT5G23040	0.016737292	0.005012997	0.004793247	523
AT4G01040	0.003252906	0.013469397	0.009821808	524
AT1G16040	0.00396628	0.021323506	0.001256908	525
AT2G40190	0.004747505	0.018153517	0.003648292	526
AT5G54750	0.00584055	0.01072254	0.009996737	527
AT1G71750	0.006035075	0.014276935	0.006267897	528
AT2G23080	0.002794859	0.004463638	0.019337324	529
AT2G18840	0.003447711	0.008500894	0.014648195	530
AT1G30090	0.005088695	0.007707253	0.013831002	531
AT5G21010	0.0022332	0.005658902	0.01876623	532
AT1G65540	0.015537293	0.005685811	0.005447382	533
AT4G24330	0.004537106	0.008124501	0.014019038	534
AT3G58020	0.00205165	0.009038018	0.015591269	535
AT3G52280	0.020978055	0.003790175	0.001947894	536
AT3G10540	0.013016502	0.000943702	0.01278461	537
AT2G44820	0.007562202	0.006062538	0.013120317	538
AT4G32010	0.007467602	0.011364857	0.007914426	539
AT3G09360	0.008528864	0.014305067	0.003950362	540
AT1G63430	0.025132172	0.001653746	0	541
AT5G52890	0.01075259	0.01503829	0.000995403	542

AT5G13890	0.021155407	0	0.005664908	543
AT3G16060	0.010617168	0.016215527	0	544
AT3G49100	0.00434467	0.019409634	0.003085506	545
AT1G10670	0.006800536	0.001233037	0.018813197	546
AT1G24050	0.005946776	0.008315722	0.01259466	547
AT5G19480	0.00604522	0.009960273	0.010894493	548
AT4G24500	0.010552473	0.003472991	0.012879221	549
AT1G25290	0.008815547	0.015703262	0.00238971	550
AT2G30700	0.003806995	0.007423414	0.015678785	551
AT5G12290	0.005585103	0.003257451	0.018077835	552
AT5G46750	0.003916914	0.005032227	0.017972769	553
AT4G24560	0.010204298	0.010376727	0.006363883	554
AT5G03100	0.00167288	0.016264451	0.009034645	555
AT1G20540	0.006353597	0.009867076	0.010755604	556
AT1G13770	0.012772881	0	0.014232668	557
AT5G33280	0.014343023	0.005083907	0.007594326	558
AT5G15880	0.00450709	0.000447619	0.022092259	559
AT1G74900	0.011166502	0.000735035	0.015145992	560
AT1G64520	0.00941155	0.006396086	0.011273515	561
AT5G67290	0.002038319	0.025069216	0	562
AT3G12210	0.019587626	0	0.007539546	563
AT3G57420	0.012849942	0.01261756	0.00167178	564
AT1G77080	0.013034061	0.007147828	0.006964827	565
AT1G04750	0.006118899	0.021028006	0	566
AT1G62680	0.000754405	0.019440641	0.006954244	567
AT3G05050	0.009192219	0.003163973	0.014814159	568
AT4G17020	0.001735476	0.003923979	0.021519162	569
AT4G00170	0.002239623	0.007172856	0.017769391	570
AT3G11200	0.006397628	0.020788086	0	571
AT3G01150	0.000718469	0.003029515	0.023469357	572
AT4G03635	0.01590295	0	0.011325479	573
AT2G01720	0.007428841	0.008498748	0.011302304	574
AT4G31300	0.018099744	0.005374064	0.003758199	575
AT5G41150	0.011620027	0.012673229	0.002982101	576
AT3G09890	0.005256678	0.006949092	0.015094678	577
AT3G62360	0.013754044	0.012188572	0.001387536	578
AT4G35890	0.003089581	0.010652322	0.013592569	579
AT3G05420	0.01232538	0.007707708	0.007337975	580
AT5G13300	0.013826749	0.013574122	0	581
AT4G32130	0.002922651	0.005861034	0.018633009	582
AT5G49580	0.014150942	0.002253073	0.011018705	583
AT1G30000	0.016681079	0.006288589	0.004502496	584

AT3G08840	0.013401778	0.003980456	0.010095056	585
AT1G11090	0.005720952	0.00662447	0.015146208	586
AT3G06440	0.010957882	0.004974547	0.01157576	587
AT1G06130	0.004479521	0.006985075	0.016067402	588
AT3G12180	0.010752805	0.016788842	0	589
AT5G43940	0.005617429	0.012826985	0.009113263	590
AT5G47090	0.009341096	0.00139572	0.016842047	591
AT5G61840	0.00210289	0.008763385	0.016718172	592
AT5G43710	0.011058458	0.01416843	0.002391527	593
AT5G36230	0.006115359	0.005124557	0.016420173	594
AT1G76260	0.008574612	0.017021204	0.002081248	595
AT3G56510	0.003716779	0.007502595	0.016487409	596
AT3G56760	0.006987546	0.00997435	0.010786673	597
AT3G02680	0	0.017486211	0.010272463	598
AT4G17910	0.018826464	0.008401492	0.000561568	599
AT1G22020	0.00526324	0.010867549	0.011688786	600
AT1G28490	0.00995835	0.006798945	0.011073209	601
AT3G51850	0.005638729	0.01044813	0.011751109	602
AT3G11890	0.002561446	0.008484344	0.016796733	603
AT2G21230	0.003744294	0.004653193	0.019496268	604
AT3G20650	0.007117558	0.011229821	0.009608345	605
AT3G54610	0.002772463	0.00440524	0.020827775	606
AT1G44810	0.005980422	0.017025149	0.005009998	607
AT2G14120	0.005473863	0.016476118	0.006103396	608
AT3G59360	0.005251139	0.004733475	0.018124262	609
AT5G65940	0.002911455	0.007294969	0.017927702	610
AT3G17240	0.008243334	0.017896293	0.002007652	611
AT1G34020	0.016232504	0.008040865	0.003944919	612
AT1G02330	0.010266792	0.011524185	0.006469576	613
AT4G00710	0.015052517	0.003812182	0.009401771	614
AT2G44770	0.010683362	0.007624682	0.009997541	615
AT1G27000	0.010160119	0.01276733	0.005395582	616
AT2G18960	0.01270153	0.01563226	0	617
AT1G10840	0.007096834	0.003164845	0.018077041	618
AT5G48340	0.007144853	0.010146364	0.011065334	619
AT1G34270	0.013197767	0.006404138	0.00877994	620
AT1G22920	0.002687276	0.002510793	0.023185229	621
AT1G54680	0.005116104	0.018724947	0.004557142	622
AT3G07680	0.006669883	0.008985221	0.012743975	623
AT2G38880	0.004737772	0.018642669	0.005042051	624
AT4G39520	0.002949389	0.004066979	0.021544118	625
AT5G08440	0.01844241	0.004335434	0.00578323	626

AT3G02720	0.005742034	0.008484518	0.014339753	627
AT3G54010	0.01600571	0.008181595	0.004397029	628
AT2G38680	0.006625929	0.003406082	0.018582944	629
AT1G67250	0.008286474	0.009662653	0.010704413	630
AT5G64440	0.005697347	0.013768463	0.009196451	631
AT3G17880	0.004420735	0.014560489	0.009704989	632
AT1G13560	0.006211168	0.005452319	0.017066833	633
AT1G13870	0.002687729	0.012409268	0.013663269	634
AT3G29160	0.007068935	0.002272567	0.0194212	635
AT5G11560	0.016430103	0.010738159	0.001608859	636
AT4G15840	0.006380914	0.000889168	0.021507907	637
AT3G53390	0.005731243	0.014238246	0.008836676	638
AT3G55490	0.001439455	0.01477182	0.012644649	639
AT3G49510	0	0.009262075	0.01961326	640
AT4G35570	0.01395125	0.001729198	0.013200111	641
AT3G27430	0.006494384	0.007735478	0.014674853	642
AT4G11820	0.016172983	0.012739368	0	643
AT5G08060	0.025786629	0	0.003173564	644
AT1G48050	0.010000313	0.000823122	0.018138812	645
AT5G35995	0.011458896	0.009114283	0.008400416	646
AT4G22140	0.004103484	0.008170565	0.016715763	647
AT5G58440	0.007497053	0.009411354	0.012082863	648
AT3G08910	0.004371437	0.002592457	0.022032748	649
AT3G43540	0.002385095	0.011238171	0.015398985	650
AT2G19600	0.001267187	0.016488713	0.011287658	651
AT5G59710	0.003004249	0.010874466	0.015188542	652
AT5G66140	0.021947118	0	0.00713323	653
AT5G19350	0.008461397	0.014592896	0.00605816	654
AT2G14835	0.000422585	0.00430469	0.024386973	655
AT1G54140	0.020936534	0.000302829	0.007884809	656
AT1G43900	0.003420138	0.00432142	0.021400434	657
AT5G38600	0.003234634	0.010817778	0.015108772	658
AT1G71900	0.002266088	0.008364047	0.018541471	659
AT1G49350	0.005281591	0.01777194	0.00620792	660
AT1G23950	0.004201685	0.016851735	0.008209926	661
AT3G59430	0.01602299	0.013308177	0	662
AT1G48230	0.010026982	0.004119731	0.015190612	663
AT4G27610	0.001685223	0.005131648	0.022574029	664
AT1G17040	0.010256784	0.019150197	0	665
AT2G18710	0.00609834	0.004425657	0.018905502	666
AT3G26950	0.00135156	0.013386214	0.014732475	667
AT3G18060	0.005828413	0.013943694	0.00971588	668

AT4G00231	0.008121595	0.004296105	0.017106207	669
AT5G53340	0.005777215	0.003029076	0.020720862	670
AT1G22970	0.004026984	0.00527568	0.02022605	671
AT5G61760	0.004883997	0.024737521	0	672
AT5G22400	0.011491543	0.00539895	0.012756985	673
AT4G14570	0.00465021	0.018500728	0.006500079	674
AT1G78560	0.006278161	0.017562321	0.00583066	675
AT2G28520	0.009680323	0.01026563	0.009784543	676
AT4G19560	0.015211109	0.01278575	0.001735472	677
AT1G76060	0.014727373	0.013970207	0.001047922	678
AT1G65270	0.006584939	0	0.023166051	679
AT2G31710	0.014997217	0.010923402	0.003843449	680
AT5G47760	0.006583967	0.018817593	0.004394995	681
AT1G10940	0.011534859	0.00603347	0.012229559	682
AT2G28380	0.009832652	0.008871776	0.01114696	683
AT4G34660	0.004968041	0.007070906	0.017869068	684
AT1G31020	0.005800654	0.008496057	0.015633574	685
AT1G53850	0.009879541	0.004839	0.015224281	686
AT3G12050	0.006415757	0.008857332	0.014685006	687
AT5G41130	0.005290153	0.017137062	0.007532648	688
AT3G57890	0.013339749	0.003517059	0.013115722	689
AT5G59290	0.020104639	8.51E-05	0.009814685	690
AT1G28340	0.003957604	0.006319374	0.0197657	691
AT2G26510	0.004499187	0.010007994	0.015543141	692
AT1G17720	0.00771511	0.010922825	0.011441104	693
AT5G06420	0.002824754	0	0.027259956	694
AT3G57750	0.004192274	0.018601662	0.007308219	695
AT1G14830	0.003799834	0.01568691	0.010616411	696
AT5G36170	0.003955913	0.01232593	0.013909332	697
AT3G59910	0.011691064	0.011405322	0.007105547	698
AT1G04070	0.007115616	0.013265248	0.009833685	699
AT1G61790	0.006659995	0.005544314	0.0180158	700
AT5G65250	0.010289245	0.007140268	0.012815741	701
AT4G27690	0.003690912	0.015661775	0.010894279	702
AT2G29560	0.009005634	0.001090449	0.020153533	703
AT4G31410	0.00662686	0.005365131	0.018282807	704
AT2G43465	0.00413374	0.025316633	0.000846926	705
AT2G45640	0.017348049	0.007931186	0.005025508	706
AT2G40540	0.013551408	0.016773846	0	707
AT3G33520	0.006235362	0.012550731	0.011550747	708
AT2G24520	0.019909543	0.001715985	0.008735993	709
AT3G45740	0.004697794	0.010983636	0.014703541	710

AT5G22370	0.008573942	0.012315171	0.009499748	711
AT3G56840	0.006125469	0.01739288	0.006890069	712
AT1G47720	0.011404651	0.007084697	0.011921827	713
AT1G51720	0.007282033	0.007583999	0.015550218	714
AT4G02680	0.008878592	0.007623509	0.01394351	715
AT5G38360	0.011569089	0.015008997	0.003877988	716
AT1G77850	0.005832189	0.020178598	0.004452232	717
AT1G05500	0.005371981	0.004044949	0.021057109	718
AT3G61140	0.007173669	0.003889829	0.019417412	719
AT4G17370	0.006664216	0.009327367	0.014495015	720
AT3G17205	0.0186077	0.000619416	0.011259711	721
AT4G14965	0.01490687	0.008168504	0.007435776	722
AT2G44090	0.011376547	0.004341399	0.014802834	723
AT5G06430	0.021575743	0.003554875	0.005396281	724
AT3G06270	0.007442306	0.015685978	0.007413296	725
AT4G37130	0.021621723	0	0.008942358	726
AT2G17190	0.003294233	0.009472609	0.01783263	727
AT5G61970	0.004832977	0.009159001	0.016609375	728
AT5G26760	0.010493416	0.00817223	0.012011	729
AT5G14250	0.002958572	0.004148351	0.023583613	730
AT2G40730	0.003117431	0.011986793	0.015588931	731
AT5G23395	0.006799868	0.00024675	0.023700617	732
AT3G16760	0.006735335	0.024020569	0	733
AT3G26990	0.002271192	0.013065939	0.015456837	734
AT1G06870	0.006095544	0.007700236	0.017004894	735
AT4G26650	0.011975186	0.018889392	0	736
AT1G57700	0.005811588	0.025073952	0	737
AT4G25610	0.003961954	0.010635587	0.016295081	738
AT2G02910	0.015047198	0.015846892	0	739
AT5G20930	0.010435826	0.006647685	0.013829819	740
AT3G47850	0.00802731	0.010068407	0.012819577	741
AT5G50340	0.005306184	0.008570865	0.017041292	742
AT5G51940	0.00810996	0.006786013	0.016033192	743
AT5G50960	0.007282102	0.011320704	0.01233331	744
AT1G14670	0.007331453	0.015487339	0.008147304	745
AT5G14140	0.008629462	0	0.022345445	746
AT5G47890	0.014736281	0.007310093	0.008944454	747
AT4G37280	0.005413319	0.005370653	0.020246338	748
AT4G12650	0.003432695	0.007305256	0.020295863	749
AT1G03260	0.003136538	0.013157846	0.014740702	750
AT1G74230	0.005586605	0.010291486	0.01518332	751
AT3G07950	0.005765477	0.003200281	0.022108745	752

AT3G26134	0	0.031083535	0	753
AT5G09390	0.005085723	0.010665457	0.015336009	754
AT3G44340	0.013558058	0.003783047	0.013750907	755
AT2G44440	0.00812491	0.018870069	0.004103862	756
AT3G55260	0.00503896	0.007497107	0.018574546	757
AT2G32850	0.005022559	0.012154413	0.013974664	758
AT5G65060	0.004074433	0.00595438	0.021123345	759
AT5G66010	0.015122773	0.003394462	0.012653592	760
AT5G26940	0.002307858	0.010966932	0.017992929	761
AT1G12410	0.007337375	0.009884421	0.014082361	762
AT1G26180	0.006492756	0.013781807	0.011050466	763
AT5G63000	0.006364654	0.017349299	0.007657739	764
AT1G06770	0.012158261	0.004466045	0.014758319	765
AT4G01210	0.011906065	0.007729152	0.011747849	766
AT5G20920	0.009535988	0.00879848	0.013062073	767
AT4G32660	0.006128247	0.008679619	0.016620036	768
AT3G04310	0.017520162	0.00259177	0.011337455	769
AT1G54370	0.005591085	0.007872398	0.018012821	770
AT4G12770	0.010476436	0.008346705	0.012718442	771
AT1G55915	0.007419477	0.024150974	0	772
AT1G09840	0.009497244	0.005249141	0.016827645	773
AT1G12120	0.006003872	0.015932457	0.009674623	774
AT1G71696	0.003531225	0.00971525	0.018390489	775
AT1G12470	0.011814892	0.013972689	0.005876934	776
AT1G45233	0	0.023507697	0.008166801	777
AT5G43822	0.004017174	0.020767351	0.006939399	778
AT1G24267	0.000684419	0.004450818	0.026594968	779
AT1G17130	0.00410021	0.005927536	0.021709104	780
AT3G51050	0.011768329	0.001970935	0.018058858	781
AT2G17790	0.01571391	0.016110052	0	782
AT4G15880	0.00424637	0.01858665	0.009001001	783
AT2G34520	0.012017764	0.007910425	0.011915146	784
AT5G08190	0.003142475	0.008684133	0.020029179	785
AT3G49390	0.004209556	0.021050595	0.006620829	786
AT1G35470	0.00615978	0.005579091	0.020163912	787
AT2G27350	0.008821829	0.008608012	0.014480135	788
AT2G20650	0.010316178	0.021595585	0	789
AT2G27020	0.010993298	0.004704995	0.016214799	790
AT2G01330	0.001611649	0.008297898	0.022013195	791
AT3G21280	0.00708482	0.005657322	0.019181893	792
AT5G64830	0.006956007	0	0.025018371	793
AT1G04230	0.000672846	0.022521782	0.008792925	794

AT1G35510	0.003401299	0.01456691	0.01405982	795
AT1G69340	0.005900286	0.005318707	0.020823253	796
AT5G27650	0.006925745	0.020058304	0.005074011	797
AT1G19430	0.009805056	0.001028651	0.021236	798
AT3G06650	0.010926811	0.003639975	0.017529937	799
AT4G08810	0.006859996	0.01331694	0.011929852	800
AT1G73875	0.003155891	0.012715869	0.016248601	801
AT4G25970	0.011775937	0.005002287	0.015356803	802
AT4G27050	0.017760369	0.01085312	0.003522533	803
AT2G03450	0.008051609	0.016795157	0.007296763	804
AT4G18372	0.010235181	0.02191183	0	805
AT5G42990	0.013890719	0.010031336	0.008233251	806
AT2G39760	0.007454983	0.005875179	0.018842521	807
AT3G03550	0.018223087	0.006144956	0.007829759	808
AT5G24340	0.002514172	0.010242088	0.019448556	809
AT1G18580	0.001528133	0.012644678	0.018035849	810
AT1G77480	0.011297192	0.007613427	0.013330975	811
AT2G40800	0.00610938	0.014862203	0.011270925	812
AT3G61240	0.002646747	0.005553445	0.024047656	813
AT4G08240	0.015178805	0	0.017069379	814
AT3G07480	0.01266785	0.015171349	0.004422602	815
AT1G05780	0.011954165	0.005726371	0.01460534	816
AT4G32180	0.006168616	0.010840658	0.015281802	817
AT1G10950	0.002825413	0.003620233	0.025891908	818
AT2G24640	0.009522618	0.022826285	0	819
AT1G18490	0.005948386	0.01048324	0.015923939	820
AT4G31920	0.011717177	0.002622865	0.018016154	821
AT2G23520	0.013247582	0.003653961	0.015463099	822
AT5G58800	0.00682804	0.025582457	0	823
AT1G65430	0.005268461	0.018843236	0.008315101	824
AT2G29900	0.009284704	0.005443098	0.017725589	825
AT5G03290	0.006011571	0.022117996	0.004329999	826
AT2G25300	0.001043391	0.011295668	0.020169698	827
AT5G09860	0.00177508	0.006696395	0.024045733	828
AT3G26618	0.006022216	0.004256779	0.022276093	829
AT2G41490	0.01192886	0.010166703	0.010474464	830
AT3G57710	0.00723981	0.003099651	0.022237307	831
AT1G52730	0.006240139	0.008437716	0.017916754	832
AT1G48160	0.008791137	0.005493625	0.018335051	833
AT3G03740	0.008025697	0.009732148	0.014895868	834
AT3G15970	0.003122956	0.023525097	0.00600846	835
AT4G12340	0.00332283	0.00169038	0.027728514	836

AT4G16143	0.004180443	0.000951465	0.027616181	837
AT1G10290	0.00599823	0.008215295	0.018535575	838
AT4G02840	0.009165417	0.007745403	0.01589295	839
AT2G30120	0.002205585	0.010122681	0.020496283	840
AT1G28680	0.025200042	0.007633142	0	841
AT3G54480	0.006237999	0.005540035	0.021068662	842
AT5G61830	0.005114888	0.004262453	0.023477832	843
AT1G55750	0.003119927	0.007546515	0.022208185	844
AT3G12290	0.010613933	0.017222173	0.005060788	845
AT5G52530	0.015274847	0.016967591	0.000674899	846
AT1G56590	0.000863774	0.002220335	0.029861236	847
AT2G04630	0.015610978	0	0.017352818	848
AT4G09520	0.001557633	0.013616587	0.017800317	849
AT1G28760	0.011573301	0.001858203	0.019558293	850
AT2G18465	0.008693386	0.000529697	0.023784904	851
AT3G45770	0.007817457	0.010020785	0.015197077	852
AT4G01370	0.007362127	0.0195982	0.006080426	853
AT5G38830	0.006324577	0.00965832	0.017065634	854
AT3G07270	0.011945796	0.006198321	0.014908821	855
AT3G60820	0.004588445	0.004622135	0.023848307	856
AT2G35740	0.002102006	0.005351294	0.025609486	857
AT3G48890	0.010987138	0.019585808	0.002494636	858
AT5G51020	0.01022237	0.007808273	0.015088972	859
AT1G49590	0.00868249	0.008716922	0.015738644	860
AT2G43770	0.006329877	0.000972027	0.025873962	861
AT3G13445	0.010512308	0.009438296	0.013230749	862
AT2G25570	7.39E-06	0.01840477	0.014769671	863
AT1G67325	0.018428292	0	0.014754718	864
AT3G13235	0.011393273	0.010500359	0.011295876	865
AT5G27740	0.007340937	0.010683554	0.015179503	866
AT2G01270	0.005853718	0.016696701	0.01065739	867
AT1G27070	0.021439873	0.009485947	0.002288289	868
AT1G06200	0.005193687	0.003035995	0.024986558	869
AT3G02050	0.010952845	0.020126381	0.002138876	870
AT3G12200	0.006489478	0.004437214	0.022320815	871
AT3G62620	0.004735931	0.011723372	0.01681015	872
AT1G31280	0.025575215	0.005536979	0.002164055	873
AT5G65685	0.009395663	0.003158156	0.02072941	874
AT4G35760	0.003925111	0.023840434	0.005519956	875
AT3G17920	0.022657475	0.010083675	0.000550491	876
AT2G46060	0.008447123	0.024852606	0	877
AT1G73440	0.008889123	0.009352535	0.015066819	878

AT1G67440	0.000666355	0.016906707	0.015753073	879
AT5G63680	0.019308521	0.006220119	0.00780567	880
AT1G16825	0.013116019	0.01067021	0.009569643	881
AT1G63855	0.017045391	0.004753079	0.011563959	882
AT3G03773	0.006900195	0.016443532	0.010019193	883
AT1G11630	0.005809156	0.006683359	0.020874934	884
AT1G18480	0.008551924	0.02132073	0.003516062	885
AT5G66950	0.00815058	0.009646478	0.015598759	886
AT5G21326	0.008467688	0.016919898	0.008026627	887
AT2G28230	0.008298832	0.002868935	0.022250162	888
AT2G47170	0.006730487	0.015652125	0.011038665	889
AT4G35220	0.005486142	0.006332766	0.021613261	890
AT1G17370	0.014757332	0.014564576	0.004116608	891
AT1G47200	0.010266208	0.00298709	0.020287895	892
AT1G50500	0.006568327	0.007882134	0.019091827	893
AT4G33410	0.004696706	0.004297126	0.024576695	894
AT5G04710	0.005440545	0.008616487	0.019516894	895
AT2G17980	0.006934416	0.007870359	0.018816918	896
AT2G35736	0.018639736	0	0.01501014	897
AT5G46630	0.005236072	0.003877622	0.024539146	898
AT3G02860	0.004742241	0.004821085	0.02409226	899
AT2G01350	0.008619774	0.007546673	0.017489888	900
AT2G47310	0.001924116	0.014412162	0.017325287	901
AT4G09580	0.008350527	0.009772939	0.015538473	902
AT5G38460	0	0.006264733	0.027426948	903
AT3G52610	0.009623056	0.003016793	0.021058499	904
AT4G30580	0.007431291	0.013525553	0.012747042	905
AT2G17970	0.006741465	0.01792967	0.009042205	906
AT2G40090	0.002507897	0.009213817	0.021999328	907
AT3G59520	0.006521562	0.000727803	0.026496758	908
AT5G46210	0.00429729	0.009583015	0.019878859	909
AT4G25280	0.014176245	0.002870565	0.016765642	910
AT2G34250	0.007017336	0.022929813	0.003872617	911
AT4G29860	0.013208607	0.00785015	0.012770665	912
AT3G11850	0.016896046	0.014665155	0.002277113	913
AT5G51280	0.001188439	0.01466827	0.01800086	914
AT1G21190	0.014073161	0.007374616	0.012436538	915
AT3G05070	0.005760151	0.00192837	0.026248398	916
AT3G16270	0.006945231	0.015171208	0.011835659	917
AT5G48970	0.004687159	0.012812727	0.016483172	918
AT1G77140	0.003314851	0.013573014	0.017095695	919
AT5G44450	0.007551618	0.010205968	0.016233605	920

AT1G68070	0.008353649	0.019414033	0.006232211	921
AT1G50140	0.008973206	0.006276289	0.01882587	922
AT2G16930	0.007147541	0.020954938	0.005990841	923
AT3G04780	0.026062482	0.003058727	0.004977768	924
AT4G18380	0.010644729	0.01131328	0.012141681	925
AT2G30710	0.002348759	0.003358278	0.028396936	926
AT5G41770	0.005632291	0.007306776	0.021204803	927
AT2G15730	0.00756162	0.026585413	0	928
AT4G16770	0.012051609	0.020997994	0.001127278	929
AT1G79650	0.009984287	0.008565881	0.015654171	930
AT5G35160	0.007294208	0.002857121	0.024070364	931
AT2G18110	0.012134001	0	0.022099555	932
AT3G46440	0.013799698	0.020453053	0	933
AT3G33530	0.00965009	0.004889606	0.019722674	934
AT5G17250	0.017561742	0.007217561	0.009495019	935
AT1G64430	0.009478694	0.00565184	0.019144418	936
AT4G09630	0.019370983	0.014904022	0	937
AT5G09810	0.0074846	0.008282898	0.018509746	938
AT3G13480	0.016884813	0.011668669	0.005787716	939
AT2G46090	0.005115983	0.00811988	0.021106745	940
AT4G24470	0.003760289	0.016219135	0.014476677	941
AT2G30050	0.012675977	0.004344662	0.017444983	942
AT2G32810	0.010833113	0.011551574	0.012102432	943
AT5G16050	0.004889993	0.014795495	0.0148192	944
AT4G31040	0.004997349	0.007067131	0.022496621	945
AT3G50880	0.00526908	0.013900863	0.015400106	946
AT1G80910	0.004395586	0.008088227	0.022092762	947
AT5G18475	0.009727893	0.024864894	0	948
AT4G38630	0.005335405	0.01367987	0.015594935	949
AT1G23900	0.014416328	0.009207914	0.011021335	950
AT2G19430	0.002265521	0.018888523	0.013495071	951
AT1G64440	0.004162392	0.015506873	0.014986638	952
AT2G29390	0.011941284	0.007549934	0.015179075	953
AT2G13290	0.010064153	0.023236383	0.001391776	954
AT3G06340	0.009648216	0.014482015	0.010592524	955
AT5G18410	0.017979464	0.015138925	0.00160657	956
AT5G43320	0.011327408	0	0.023409062	957
AT1G61150	0.003822287	0.00665842	0.02426273	958
AT5G44860	0.005049139	0.007425355	0.022281825	959
AT1G01820	0.012990488	0.01740783	0.00439148	960
AT3G58580	0.003819674	0.011866814	0.019106744	961
AT2G28590	0	0.015611556	0.019185923	962

AT2G03430	0.007595746	0.004267435	0.022939865	963
AT1G55520	0.004073706	0.00083223	0.029899358	964
AT1G56310	0.015657635	0.01917447	0	965
AT4G28030	0.012778064	0.009226232	0.012835504	966
AT2G20790	0.005463353	0.013820779	0.015639006	967
AT1G50120	0.004742481	0.00331043	0.026871574	968
AT1G08350	0.000944322	0.033990551	0	969
AT3G59390	0.000130511	0.010719414	0.024107445	970
AT5G11720	0.012610609	0.007263317	0.015087563	971
AT2G28800	0.005493383	0.01387146	0.015632766	972
AT1G52320	0.011658753	0.014578	0.008773298	973
AT1G79210	0.009111573	0.008449626	0.01745632	974
AT5G18280	0.004318088	0.011405292	0.019299315	975
AT3G55520	0.009809551	0.00920117	0.016098619	976
AT5G01780	0.01348275	0.014213362	0.007416095	977
AT3G15470	0.010098364	0.011729083	0.013285094	978
AT5G51510	0.007004678	0.017549344	0.010565428	979
AT5G53000	0.003295115	0.012136563	0.019714808	980
AT5G36880	0.010683023	0.020456717	0.004049245	981
AT1G73350	0.005393918	0.004590171	0.025205979	982
AT3G22480	0.014304692	0.011652073	0.009242063	983
AT5G63840	0.007704597	0.005207197	0.022310306	984
AT3G04580	0.032454356	0.000701771	0.002094201	985
AT3G15980	0.018564555	0.012860304	0.00383247	986
AT5G19570	0.03259158	0	0.002676061	987
AT4G35530	0.005488622	0.015723627	0.014070021	988
AT2G35320	0.006987423	0	0.028297365	989
AT3G56680	0.005356562	0.008580035	0.021351259	990
AT2G44270	0.00638515	0.003036014	0.025872855	991
AT3G58760	0.002648706	0.008336564	0.0243167	992
AT5G07360	0.006951826	0.010598771	0.017773606	993
AT3G10850	0.004821356	0.009295366	0.021219084	994
AT5G46170	0.005017795	0.021615829	0.008752092	995
AT1G71270	0.023981654	0.006302472	0.005113753	996
AT5G60160	0.007221239	0.006010736	0.022169441	997
AT5G51430	0.00427476	0.006922173	0.024219489	998
AT4G38120	0.015262541	0.007961574	0.012211914	999
AT3G12100	0.006559315	0.01141753	0.017482145	1000
