# Supplementary material for: Identifying stably expressed genes from multiple RNA-Seq data sets
Source: PeerJ. 2016 Dec 20;4:e2791. doi: 10.7717/peerj.2791 (PMC5178351; doi:10.7717/peerj.2791)
Supplement: Table S4 [file peerj-04-2791-s004.pdf]

Supplemental Table S4: Top 1000 stably expressed genes from the multiple-tissue group.

Gene	between_sample	bewtween_treatment	between_experiment	Rank
AT1G22860	0.003072178	0.000232994	0.002619774	1
AT5G42470	0.001904835	0.002907751	0.001119281	2
AT4G31440	0.000841943	0.001642905	0.003895858	3
AT2G32760	0.001869758	0.002571744	0.002880964	4
AT1G75420	0.001182509	0.001419035	0.004955079	5
AT2G25740	0.000355979	0.001616381	0.005644076	6
AT5G58100	0.002015618	0.000792049	0.005146978	7
AT3G45190	0.001509467	0.001593984	0.004982953	8
AT1G75340	0.001556751	0.000430861	0.006181722	9
AT1G02145	0.001369563	0.003019027	0.004160477	10
AT2G04940	0.005458214	0.001869777	0.001681321	11
AT3G27530	0.004477778	0	0.005266896	12
AT3G05090	0.002433291	0	0.007398248	13
AT3G15920	0.001776959	0.000860298	0.007358362	14
AT4G26100	0.001058985	0.003766883	0.005232819	15
AT1G18560	0.00329132	0.004293597	0.002492992	16
AT4G35140	0.003092366	0.0033662	0.003852428	17
AT1G55520	0.004711491	0.000850234	0.004774376	18
AT1G11060	0.002327596	0	0.008096704	19
AT2G35738	0.00147566	0.00925527	0	20
AT3G06340	0.002494966	0.0036549	0.004654423	21
AT1G35470	0.001266765	0.00027702	0.009320244	22
AT3G12640	0.00163348	0.00117468	0.008513696	23
AT2G42030	0.002070559	0.003525382	0.00574944	24
AT3G23900	0.002294702	0.00096741	0.00840481	25
AT5G20170	0	0.001103102	0.010679979	26
AT1G54390	0.001864957	0.000901361	0.009047494	27
AT1G73440	0.008730322	0	0.003167748	28
AT5G53180	0.007126134	0.000305209	0.004655612	29
AT2G47760	0.003866727	0.00307082	0.00519625	30
AT2G23140	0.006578466	0.003586861	0.002032292	31
AT5G37380	0.00284648	0.000951452	0.008571872	32
AT1G80040	0.003126111	0.001000258	0.008281264	33
AT3G58030	0.001538203	0.000948808	0.009963311	34
AT2G20000	0.002501488	0.00119512	0.009045708	35
AT5G18390	0.001088982	0	0.011671001	36
AT3G09180	0.001305675	0.000413316	0.011070301	37
AT5G25060	0.001771573	0.00331976	0.007710301	38

AT1G69670	0.003788862	2.38E-17	0.009014794	39
AT1G51690	0.002425944	0.004810954	0.005636194	40
AT5G04670	0.004012736	0.00332475	0.005917502	41
AT5G51340	0.005572088	0	0.007701841	42
AT1G11880	0.000393714	0.003495648	0.009420417	43
AT4G17020	0.00386285	0.003438854	0.006033841	44
AT2G46180	0.006774273	0	0.006786414	45
AT5G48340	0.004249958	0.001920473	0.007447348	46
AT3G61350	0.006417872	0.00120634	0.006116206	47
AT2G22720	0.002985314	0.002488104	0.008291332	48
AT4G31441	0.004848264	0.001962822	0.007006983	49
AT5G51880	0.003980191	0	0.009849261	50
AT1G54610	0.002786117	0.001828769	0.009233771	51
AT1G01930	0.002484999	0.000399294	0.011043884	52
AT2G32910	0.000743274	0.001880002	0.011336391	53
AT2G27350	0.002105947	0.003050295	0.008830133	54
AT2G04740	0.002921695	0.006900241	0.004193665	55
AT5G13970	0.001189287	0.002749024	0.010115346	56
AT5G50870	0.007121021	0	0.006955415	57
AT1G63110	0.002488959	0.001527716	0.01022581	58
AT2G03150	0.004729729	0	0.009629532	59
AT1G49590	0.004572501	0	0.00980091	60
AT2G28310	0.003530438	0.002854086	0.00803627	61
AT2G28390	0.003441214	0	0.01107629	62
AT4G26450	0.002105079	0.00026032	0.012254425	63
AT5G16610	0.001496604	0.001850278	0.011315862	64
AT1G52630	0.005803725	0.00078436	0.008298907	65
AT2G45690	0.002529222	0.000811504	0.011720707	66
AT5G41480	0	0.002263186	0.012807562	67
AT2G44150	0.002324439	0.000344454	0.012482932	68
AT5G20930	0.00146735	0.004826576	0.008939243	69
AT3G54540	0.001846878	0.00539809	0.008026195	70
AT5G16520	0.003335613	0.002096321	0.009864983	71
AT1G64840	0.005068751	0.000771504	0.009477237	72
AT3G51310	0.002819031	0.002507556	0.009998315	73
AT3G04490	0.008315588	0	0.00708779	74
AT1G67960	0.0028887	0.001947641	0.010635903	75
AT4G12640	0.001766338	0.000684886	0.013029445	76
AT2G21230	0.002613327	0.001965875	0.010962638	77
AT3G27700	0.003804707	0	0.011799836	78
AT3G06670	0.003687779	0.00161692	0.010318633	79
AT2G36480	0.0050819	0	0.010588916	80

AT3G45740	0.004584413	0.001867958	0.009228195	81
AT4G02560	0.003838669	0.001787346	0.010061841	82
AT2G44680	0.003177364	0.001113676	0.011405601	83
AT3G12680	0.001848056	0.000941662	0.012926352	84
AT3G28430	0.005195846	0.001905962	0.008645089	85
AT2G30980	0.002818924	0.001565344	0.011464548	86
AT1G08030	0.000801537	0.007460933	0.007609735	87
AT2G44530	0.005525099	0.001169726	0.00919319	88
AT4G33890	0.001859308	0.002333987	0.011714939	89
AT2G47210	0.003261632	0.002809637	0.00993551	90
AT4G08455	0.003475309	0.003805373	0.008805594	91
AT2G26460	0.001526658	0.002745795	0.011845486	92
AT5G53540	0.002518164	0.007076081	0.006581566	93
AT1G79280	0.012385317	0	0.003809595	94
AT2G44750	0.005988173	0.0016311	0.008605918	95
AT3G07890	0.004156828	0.001305987	0.010765984	96
AT2G29210	0.003025926	0.000514362	0.012691581	97
AT5G19280	0.001259504	0.004743402	0.010281053	98
AT1G55630	0.001804207	0.004327147	0.010193949	99
AT5G46210	0.003441161	0.000444919	0.012441569	100
AT4G36960	0.001552924	0.00066726	0.014146449	101
AT5G09860	0.004540964	0	0.011834069	102
AT5G49060	0.003359512	9.45E-05	0.012969917	103
AT3G05850	0.000891017	0.001231763	0.014313387	104
AT4G28910	0.003388216	7.66E-05	0.013021771	105
AT3G54190	0.001596894	0.002212251	0.012730983	106
AT1G17760	0.00403277	0.005785492	0.006745123	107
AT5G64470	0.002656757	0.002038487	0.011930974	108
AT2G39260	0.004354592	0.000422382	0.011858282	109
AT1G13870	0.00601238	0.00161946	0.009039854	110
AT3G20290	0.001299255	0.001823378	0.013554146	111
AT5G08440	0.002917825	0.000608417	0.013172713	112
AT2G43070	0.00091488	0.003558995	0.012249151	113
AT2G30880	0.002306087	0.000781892	0.013675201	114
AT5G41150	0.002485668	0.003330751	0.01096224	115
AT5G18230	0.00224481	0.001229531	0.013304719	116
AT5G44150	0.004889073	0.001739011	0.010324252	117
AT1G50140	0.004323233	0.003132826	0.009586329	118
AT4G17150	0.00495763	0.001201316	0.01091502	119
AT3G43240	0.002499304	0.000386761	0.01423106	120
AT3G14860	0.001339229	0.000874645	0.014917453	121
AT5G65540	0.0014268	0.002230366	0.013538685	122

AT4G31200	0.003095835	0.00126511	0.012837431	123
AT4G32360	0.006064467	0	0.011146405	124
AT2G35330	0.002159429	0.000239133	0.01485525	125
AT2G20330	0.00109929	0.00082484	0.015336922	126
AT5G21040	0.002878	0.000845154	0.013592641	127
AT2G40630	0.005047947	0.006079007	0.006214621	128
AT3G09740	0.00559871	0.004913171	0.006987488	129
AT5G01400	0.006621749	0.000251505	0.010641478	130
AT5G67530	0.001556027	0.000897928	0.015085436	131
AT3G15010	0.008606044	0	0.009008227	132
AT4G37120	0.005851053	0.001329784	0.010438977	133
AT2G40980	0.00480309	0.005343939	0.007522488	134
AT1G75950	0.00939999	0	0.008336758	135
AT1G76630	0.004175142	0	0.013562227	136
AT2G32700	0.004493339	0	0.013311751	137
AT2G38950	0.001665811	0.003009849	0.013157923	138
AT5G01270	0.001234095	0.005435194	0.011184136	139
AT2G40090	0.005362629	0.001334292	0.011193896	140
AT4G12780	0.004960397	0.003256799	0.009676594	141
AT1G64050	0.006222715	0.002190211	0.009644482	142
AT4G32272	0.00567579	0.00447366	0.007938306	143
AT1G79730	0.002545893	0.001933074	0.013629254	144
AT2G38020	0.00093973	0.002853962	0.014378513	145
AT1G30825	0.004623289	0.001107602	0.012457249	146
AT1G49350	0.005469432	0.007667981	0.005088667	147
AT1G09980	0.006180184	0.002376314	0.009889895	148
AT3G13438	0	0.011231401	0.007270679	149
AT1G50730	0.003661112	0.001244438	0.013599841	150
AT3G17850	0.006373515	0	0.012258841	151
AT2G17390	0.003966937	0.00132514	0.013400473	152
AT2G44710	0.002982373	0.001900781	0.013814215	153
AT1G34470	0.001272808	0.001560061	0.015945614	154
AT3G23980	0.005611764	0	0.013174408	155
AT1G72340	0.002725445	0.003106531	0.012984715	156
AT4G02740	0.003350098	0.002348364	0.013223417	157
AT5G38840	0.001193609	0.005855525	0.011915413	158
AT5G06600	0.006522836	0	0.01245357	159
AT1G80790	0.003666665	0.00236538	0.013002057	160
AT2G01060	0.006396525	0.000750022	0.011898511	161
AT3G06580	0.003268012	0.004198165	0.011653821	162
AT4G32660	0.00214501	0.003265084	0.013796504	163
AT4G15010	0.004391345	0.002410342	0.012431768	164

AT3G61690	0.008451593	0	0.010825712	165
AT5G14850	0.005184271	0.001526382	0.012611084	166
AT1G04080	0.005877053	0.001061964	0.012387966	167
AT5G35930	0.006196436	0.000662487	0.012526595	168
AT4G36290	0.003271434	0.001225612	0.014901095	169
AT1G32490	0.003981924	0.001349498	0.014097104	170
AT1G76850	0.009287908	0.000249413	0.009910751	171
AT5G02910	0.006798192	0.001683153	0.011062469	172
AT2G35740	0.004645798	0.011111577	0.0037874	173
AT2G39760	0.003544864	0.000731062	0.015323048	174
AT1G19480	0.00380545	0.000960636	0.014846361	175
AT4G00650	0.007600171	0	0.012104011	176
AT1G12470	0.006264613	0	0.013467589	177
AT1G53165	0.003762413	0.002636111	0.013465274	178
AT4G24470	0.005918854	0	0.013993214	179
AT3G07300	0.008522476	0.003335154	0.008146734	180
AT2G20790	0.00155755	0.001734148	0.016786899	181
AT2G41520	0.006238913	0.002286812	0.01157042	182
AT2G36370	0.001387589	0.002661609	0.016057216	183
AT4G38170	0.003387332	6.48E-05	0.016697821	184
AT4G15880	0.002090822	0.000414911	0.017692993	185
AT4G27910	0.008207771	0.000904103	0.01111167	186
AT4G27050	0.005915845	0.001586013	0.012728885	187
AT1G66740	0.006096754	0.002425261	0.011771571	188
AT3G19420	0.002817571	0.002046276	0.015429948	189
AT1G71820	0.003880534	0.002034033	0.014426423	190
AT5G47790	0.001906481	0.000368498	0.018130812	191
AT1G55750	0.001492342	0.002094317	0.016856224	192
AT1G24120	0.006640653	0.002387546	0.011421378	193
AT3G20170	0.006911735	0.000105514	0.013498108	194
AT5G64500	0.002092457	0.00491833	0.013579573	195
AT3G17880	0.004389102	0.000827796	0.015387225	196
AT5G43130	0.003391866	0.005336041	0.011905361	197
AT2G26100	0.003067325	0.002842308	0.01476015	198
AT3G44530	0.004288152	0.002706982	0.013735245	199
AT5G62760	0.004407739	0.000155476	0.016229783	200
AT5G46630	0.003110801	0.001157939	0.016592925	201
AT1G33980	0.006554143	0	0.014315579	202
AT3G26670	0.010123979	0	0.010877979	203
AT5G19000	0.003700313	0.002739119	0.014579967	204
AT5G59710	0.003137679	0.001942453	0.015942911	205
AT5G62610	0.005786138	0.000934485	0.014320498	206

AT3G63180	0.004822355	0.009586525	0.006731608	207
AT2G33290	0.002824403	0.003992314	0.014337742	208
AT1G61030	0.003409129	0.000576157	0.017181129	209
AT3G06240	0.002823498	0.000761382	0.017636863	210
AT1G51600	0.004249585	0.002346953	0.014632051	211
AT2G39970	0.004121221	0.00313823	0.014065036	212
AT2G02090	0.001033489	0.002157032	0.018172841	213
AT5G13260	0.002284915	0.001015503	0.01806676	214
AT1G50500	0.003363039	9.29E-05	0.01794641	215
AT5G11350	0.003927474	0.000398372	0.017138207	216
AT3G50860	0.006969488	0.002332729	0.012218966	217
AT3G62240	0.003080761	0.000651477	0.017789151	218
AT4G12770	0.004586548	0.005025062	0.011909842	219
AT1G28420	0.01352344	0	0.008041361	220
AT3G17740	0.004324759	0.002121325	0.015120028	221
AT3G21290	0.004021425	0	0.017579326	222
AT1G75660	0.00533299	0.002071131	0.014285909	223
AT5G56900	0.002790807	0.012907332	0.006034156	224
AT3G21350	0.00456724	0.005769912	0.011406635	225
AT4G39680	0.003847513	0	0.017905341	226
AT1G27430	0.00619737	0	0.015577714	227
AT1G20670	0.004672799	0.000380399	0.016789818	228
AT1G04790	0.002271801	0.000805011	0.018866711	229
AT3G07220	0.006405219	0.004134769	0.011459643	230
AT3G11240	0.002943223	0.006766531	0.012339963	231
AT2G17510	0.011731857	4.00E-05	0.010286045	232
AT3G45750	0.004568495	0.001973445	0.015532349	233
AT5G49930	0.003291398	0.001907151	0.016970907	234
AT5G46400	0.009243637	0	0.012935401	235
AT5G17360	0.010762446	0.00224364	0.0091776	236
AT1G20760	0.006290457	0.00135038	0.014589232	237
AT1G10390	0.013529549	0	0.008710441	238
AT3G17205	0.005995352	0.000110202	0.016146974	239
AT5G53440	0.004687948	0.00075322	0.016827636	240
AT3G46790	0.006340847	0.007394933	0.008537494	241
AT1G43190	0.001699921	0.002970097	0.017607713	242
AT2G47980	0.004393086	0.001949027	0.015948623	243
AT1G14570	0.001201017	0.005431389	0.015689269	244
AT1G55300	0.006601534	0	0.01572654	245
AT3G46200	0.005140167	0	0.017225395	246
AT5G65180	0.002855245	0.003155457	0.016368374	247
AT4G15420	0.005516634	0.004230354	0.012708618	248

AT3G09370	0.001562572	0.002643962	0.018322485	249
AT1G34190	0.002098708	0	0.020430938	250
AT1G49710	0.003698523	0.002040848	0.016798611	251
AT3G21215	0.005820779	0.008135628	0.008610339	252
AT5G49530	0.011925254	0.003321357	0.007375001	253
AT4G17640	0.002211088	0.001245902	0.019172673	254
AT4G10130	0.008330921	0	0.014381835	255
AT4G01370	0.010658818	0	0.012077271	256
AT2G19390	0.004670202	0	0.018085749	257
AT3G08780	0.006596606	0	0.016201511	258
AT3G04460	0.004431364	0.001202167	0.017168875	259
AT2G05170	0.006053233	1.43E-17	0.016756882	260
AT4G16650	0.003750557	0.001472132	0.017610256	261
AT4G24290	0.001375504	0.002037875	0.019434179	262
AT3G59500	0.004420495	0.003136746	0.015324886	263
AT3G61800	0.002360336	0.002840064	0.017736392	264
AT2G21470	0.002513791	0.001749202	0.018686693	265
AT1G22200	0.002228429	0.001857606	0.018882634	266
AT1G09730	0.003144459	0.002534965	0.017326279	267
AT2G43260	0.012715075	0	0.010301968	268
AT4G09340	0.004359137	0.002276154	0.016393381	269
AT2G13560	0.002959152	0.005077923	0.015025684	270
AT3G04970	0.003090381	0.002491224	0.017499951	271
AT2G06210	0.003582917	0.00028461	0.01923768	272
AT3G47910	0.008758711	0.003931435	0.010442255	273
AT1G01290	0.006464547	0.000290994	0.01638962	274
AT3G26935	0.002603982	0.007117519	0.013531617	275
AT2G32170	0.001614193	0.001775775	0.019871698	276
AT2G38630	0.00079412	0.001763519	0.020739953	277
AT1G11900	9.37E-05	0.001040449	0.022167473	278
AT2G32000	0.004370325	0.000387948	0.018563573	279
AT5G65290	0.003944755	0	0.01937926	280
AT1G14510	0.006338444	0.000585431	0.016405036	281
AT2G17030	0.006411497	0.002299834	0.014642637	282
AT3G59380	0.006321372	0.00131604	0.015743587	283
AT1G31360	0.003527463	0.008258842	0.01165839	284
AT3G54860	0.003492913	0.005198049	0.014789224	285
AT2G23780	0.006408011	0.005020058	0.012062303	286
AT4G00840	0.006068923	0.00236241	0.015078622	287
AT5G16320	0.003142851	0.00357122	0.01680944	288
AT4G24550	0.002544405	0.001526691	0.019469084	289
AT5G42220	0.011786647	0	0.011765921	290

AT1G72880	0.006053253	0.005570278	0.011942705	291
AT1G07910	0.006952561	0.007745208	0.008955749	292
AT1G07980	0.00953695	0	0.014154841	293
AT1G54490	0.004009226	0.000669275	0.019057375	294
AT5G43320	0.003490321	0.002562748	0.017688249	295
AT2G05755	0.003826568	0.003557777	0.016408148	296
AT3G01150	0.001916888	0.00319096	0.018687242	297
AT5G65780	0.005605216	0.001447956	0.01678103	298
AT2G42520	0.00564036	0.00396615	0.01425666	299
AT3G55020	0.0038491	7.25E-05	0.019968536	300
AT1G80930	0.004043263	0.001561737	0.018293357	301
AT3G54480	0.008251918	0	0.015665149	302
AT4G11670	0.01258192	3.59E-06	0.011380718	303
AT3G20650	0.00192702	0.00235436	0.019702782	304
AT1G79350	0.005378649	0	0.018629791	305
AT1G74120	0.000375385	0.004105143	0.019579932	306
AT5G67610	0.00382492	0	0.020238073	307
AT2G17970	0.001213539	0.002410682	0.020463677	308
AT5G25150	0.001826515	0.000491852	0.021778946	309
AT1G68370	0.002295958	0.00440186	0.017409379	310
AT1G24706	0.00505325	0.000516664	0.018571381	311
AT1G56590	0.001364541	0.001498736	0.0213084	312
AT2G45160	0.00563054	0.011030145	0.007584457	313
AT1G33990	0.003433379	0.006334089	0.014522084	314
AT1G30470	0.003293001	0.003908712	0.017096992	315
AT3G49850	0.006925577	0.001698806	0.015689598	316
AT5G47010	0.007820142	0.000102725	0.016433739	317
AT2G27210	0.004193827	0.004099006	0.01612446	318
AT1G26830	0.004529421	0.003149672	0.016742284	319
AT2G27110	0.003420342	0	0.021051695	320
AT2G45700	0.000959207	0.002231457	0.02138141	321
AT2G41020	0.006889471	0.002464358	0.015314867	322
AT2G27600	0.00382539	0.003257695	0.017630155	323
AT2G20830	0.004224377	0.00191822	0.018629146	324
AT3G53390	0.001130845	0.001737131	0.021908337	325
AT1G80350	0.004781457	3.10E-05	0.019973883	326
AT2G38560	0.003568451	0.002788992	0.018439452	327
AT1G58350	0.003688455	0.002434086	0.018678292	328
AT2G44950	0.002870691	0.001578607	0.02036069	329
AT1G23780	0.002835101	0.002892126	0.019169102	330
AT3G23540	0.00239065	0.003783572	0.018750423	331
AT4G32120	0.001485804	0.006997571	0.016444012	332

AT3G03360	0.003374024	0.001979949	0.019615891	333
AT4G16845	0.001189915	0.001933601	0.021855037	334
AT5G63280	0.002823866	0	0.022275056	335
AT4G16100	0.006471617	0	0.018649361	336
AT1G17680	0.004017294	0.003270049	0.017841786	337
AT5G26990	0.007351471	0	0.01779641	338
AT3G49600	0.003246118	0.007546676	0.01436802	339
AT5G48970	0.015056532	0	0.0101549	340
AT4G20400	0.003329248	0.001136896	0.020775754	341
AT2G40060	0.00912541	0.004511343	0.011670388	342
AT1G03750	0.004070263	0.000849974	0.020493009	343
AT4G08320	0.001269708	0.001005693	0.023174942	344
AT2G35360	0.006467862	0.002541753	0.016481518	345
AT1G31870	0.001145648	0.003166165	0.021179836	346
AT3G05240	0.002170161	0	0.023339594	347
AT5G09400	0.00386642	0.000591223	0.021058332	348
AT5G18580	0.002111902	0.00231867	0.021109645	349
AT3G28970	0.005377298	0.000143066	0.020022929	350
AT1G13120	0.003941112	0.003871176	0.017764501	351
AT1G14650	0.004092945	0	0.021534199	352
AT1G17790	0.002125386	0.000853027	0.022669445	353
AT5G07740	0.012470133	0	0.013178958	354
AT2G23080	0.004779721	0.002184139	0.018738947	355
AT4G01210	0.011569621	0.000194575	0.013944439	356
AT1G03365	0.001440516	0.000369458	0.02395248	357
AT1G26110	0.00313366	0.000701679	0.02195603	358
AT1G61960	0.000624456	0.003401405	0.021804593	359
AT2G42640	0.014096756	0.003394169	0.008341984	360
AT1G79570	0.008010983	0.002248442	0.015573646	361
AT1G03960	0.003648986	0.001959709	0.020251342	362
AT1G63430	0.001059449	0.003155507	0.02176324	363
AT5G10630	0.003539698	0.00010932	0.022348777	364
AT1G73320	0.005467518	0.007505645	0.013053666	365
AT4G26610	0.000804711	0.003251432	0.021982373	366
AT3G53090	0.006632992	0.000219044	0.019275008	367
AT5G19450	0.005948556	0.004760819	0.015460981	368
AT3G52660	0.005147737	0.000792201	0.020233675	369
AT1G19170	0.003620719	0.001641578	0.020917021	370
AT4G23895	0.007230851	0.003039036	0.015928238	371
AT1G09020	0.003359849	0.001117385	0.021735079	372
AT1G08750	0.001344584	0.001279195	0.023598222	373
AT3G21280	0.002517676	0.002932092	0.020784156	374

AT1G51740	0.004923499	0.00613524	0.015185245	375
AT1G32050	0.004075239	0.001032439	0.021144498	376
AT3G21865	0.004879568	0.001516378	0.01986276	377
AT1G16570	0.001750927	0.002739546	0.021775057	378
AT5G40520	0.005385572	0.001154625	0.019754063	379
AT3G11890	0.004095707	0.004403526	0.017879833	380
AT5G47690	0.008677621	0	0.017708649	381
AT5G64090	0.001712052	0.004671871	0.020014962	382
AT5G51280	0.003736924	0.002605757	0.020119506	383
AT1G80860	0.009562264	0	0.016909019	384
AT1G17210	0.004897754	0.007225317	0.014359341	385
AT5G52630	0.000123854	0.005641888	0.02072608	386
AT3G50690	0.00513283	0	0.021385441	387
AT4G13870	0.004331673	0.003791131	0.018423047	388
AT5G40190	0.015018603	0	0.011539093	389
AT4G08350	0.004352601	0	0.022224058	390
AT3G10330	0.004509285	0	0.022100908	391
AT5G09680	0.005713601	2.36E-05	0.020876633	392
AT1G21170	0.003304389	0.001633585	0.021692226	393
AT5G58510	0.003839942	0.003732869	0.019088649	394
AT5G15440	0.001320752	0.009501774	0.015839769	395
AT1G29710	0.004769857	0.002212009	0.019767325	396
AT2G35320	0.006766705	0	0.019984859	397
AT5G56930	0.004383268	0.001436314	0.020963022	398
AT4G20280	0.005014494	0.002273671	0.019503992	399
AT2G22120	0.003250504	0.002546409	0.021034651	400
AT1G78910	0.005726036	0.004764179	0.016346389	401
AT5G16640	0.002784274	0.001529493	0.022541789	402
AT5G16210	0.006571245	0	0.020337277	403
AT3G19780	0.004541	0	0.022462768	404
AT5G15710	0.002336075	0.002794786	0.021882152	405
AT3G55070	0.005058038	0.003193662	0.018880493	406
AT3G11960	0.008284592	0	0.018864858	407
AT2G42240	0.000798141	0.001472589	0.024891063	408
AT1G60995	0.00319704	0.003042463	0.020950552	409
AT1G69340	0.00299152	0.000799519	0.023404462	410
AT5G19400	0.004239306	0.000977738	0.022048744	411
AT3G24040	0.00822297	0.005600096	0.013463796	412
AT1G27595	0.006628897	0.003153557	0.017528599	413
AT5G13050	0.002308394	0.000497926	0.024528564	414
AT4G05000	0.005533552	0.004266884	0.01755877	415
AT3G42790	0.006437229	0.001855508	0.019088577	416

AT5G22760	0.008550121	0	0.018846538	417
AT3G21175	0.005870252	0.000906255	0.020651981	418
AT3G26730	0.003189794	0.002670357	0.021587208	419
AT2G47330	0.000866093	0.001661311	0.024934411	420
AT1G79810	0.00170794	0.004482223	0.021273585	421
AT1G26170	0.002484774	0.002770249	0.022212324	422
AT4G10730	0.006230926	0	0.021254543	423
AT1G26120	0.00149611	0.001991783	0.0240261	424
AT5G26760	0.003643731	0.001708252	0.022163767	425
AT3G17715	0.007160861	0	0.020400271	426
AT3G07530	0	0.001701086	0.025905184	427
AT4G01880	0.009677162	0.001364371	0.016602775	428
AT4G00980	0.00299661	0.004744304	0.019915667	429
AT1G27840	0.015426214	0.001667333	0.010575545	430
AT3G15220	0.006507117	0.000965452	0.020201152	431
AT2G41350	0.00399814	0	0.023734176	432
AT5G46560	0.002063435	0.001455112	0.024264227	433
AT1G34320	0.003136556	0.00022554	0.024436008	434
AT1G61040	0.001386919	0.003539163	0.022875668	435
AT4G10790	0.001042098	0.000970641	0.025803202	436
AT4G33060	0.002710762	0.006013737	0.01909174	437
AT5G13530	0.008377957	0	0.019439333	438
AT1G13770	0.002675772	0.001403763	0.023755517	439
AT2G45320	0.004111347	0.010409911	0.013400502	440
AT4G35540	0.006715108	0.00085669	0.020354996	441
AT4G16180	0.003310345	0.002194522	0.022423684	442
AT5G01010	0.002819331	0	0.025122273	443
AT3G33530	0.008765621	0.000511598	0.018670486	444
AT1G02130	0.005112474	0	0.022857827	445
AT3G61710	0.003143976	0	0.024830515	446
AT4G14340	0.007768507	0	0.020211377	447
AT5G41940	0.001423284	0.002147347	0.024411178	448
AT3G28670	0.000761602	0.000724458	0.026501151	449
AT1G55090	0.006221531	0.001002024	0.020770081	450
AT3G48430	0.007123606	0.002061018	0.018837754	451
AT1G05430	0.00535363	0	0.022682169	452
AT5G21010	0.00315722	0	0.024882194	453
AT2G03690	0.012855926	0.001234622	0.013962222	454
AT1G61150	0.004581551	0.000405127	0.023070226	455
AT5G52040	0.00764262	0.002128059	0.018322535	456
AT2G27285	0.003893031	0	0.024250957	457
AT3G62970	0.003932202	0.005647763	0.018592411	458

AT2G27490	0.007325507	0	0.020852812	459
AT1G08420	0.005804838	0.001915125	0.02051742	460
AT1G31175	0.004651817	0.007700322	0.015894957	461
AT1G11630	0.005929648	0.002013866	0.02036576	462
AT3G19840	0.004704382	0.000339901	0.023265497	463
AT2G19600	0.002209826	0.001404829	0.024702893	464
AT3G18310	0.002274815	0.000657484	0.025423466	465
AT1G55150	0.003359864	0.002396774	0.022628428	466
AT3G55270	0.006068602	0	0.022325479	467
AT4G01290	0.008118111	3.86E-06	0.020326808	468
AT5G04920	0.000701007	0.001110173	0.026701138	469
AT3G11910	0.006788817	0	0.021739826	470
AT4G25520	0.003205128	0.004242723	0.02108603	471
AT4G29790	0.007495999	0	0.02104721	472
AT1G77300	0.011434047	0	0.017137955	473
AT1G64385	0.004306744	0	0.024279523	474
AT1G48635	0.003394954	0.000758123	0.024481018	475
AT1G08660	0.004561008	0.002404683	0.021680501	476
AT1G48760	0.004420532	0.000340464	0.023902449	477
AT4G24500	0.005053654	0.003404301	0.020237304	478
AT1G60170	0.005663384	0.002855235	0.020181031	479
AT2G21600	0.004217059	0.00400967	0.020530358	480
AT3G09320	0.005861935	0.002358632	0.020540029	481
AT1G31220	0.004087103	0.004795897	0.019904025	482
AT1G50940	0.004863137	0.005023912	0.018976207	483
AT5G44100	0.001826527	0.002045195	0.025002413	484
AT1G32130	0.002429349	0.005015156	0.021463562	485
AT2G11000	0.004347857	0.000511348	0.02410104	486
AT1G54150	0.003685768	0	0.02527511	487
AT5G01290	0.003688882	0.001904422	0.023394537	488
AT1G73875	0.007522022	0.000395578	0.021103707	489
AT3G57580	0.003409505	0.007531097	0.01812892	490
AT2G42160	0.001520155	0.004166239	0.023416911	491
AT5G66060	0.005921427	0.002462052	0.020795281	492
AT3G12350	0.011261538	0.003130332	0.014834606	493
AT5G19670	0.006065122	0.003676012	0.019492024	494
AT5G48965	0.004068854	0.002744569	0.022456188	495
AT4G38040	0.004961322	0.004653563	0.019659872	496
AT3G12940	0.002879533	0.004084713	0.022313439	497
AT1G60930	0.004531992	0.004049143	0.020745753	498
AT2G31370	0.004686441	0.004533057	0.02012477	499
AT2G42500	0.003580429	0.002007546	0.023760054	500

AT4G02010	0.003743013	0.003061061	0.022564036	501
AT5G04510	0.00387024	0.002795539	0.022704503	502
AT1G03140	0.004259405	0.006129823	0.019010897	503
AT5G04240	0.005457842	0.000339825	0.023603194	504
AT4G32330	0.00876592	0.000284916	0.020355799	505
AT5G37190	0.002386654	0.009900542	0.017140276	506
AT3G22170	0.001957252	0.001776318	0.025808409	507
AT4G11440	0.001290959	0.009001596	0.019285855	508
AT1G55170	0.004577014	0.000728581	0.024339283	509
AT3G20890	0.004312554	0.000102291	0.025246593	510
AT5G64830	0.00301385	0.004945745	0.021704346	511
AT5G60620	0.003909292	0.003551012	0.022213861	512
AT1G13320	0.003557893	0.000323845	0.025800033	513
AT4G13730	0.005907054	6.60E-05	0.023712921	514
AT1G73730	0.001171964	0.002799528	0.025714962	515
AT4G24530	0.001117664	0.005799587	0.022788511	516
AT5G60410	0.004653033	0.000624858	0.024430476	517
AT3G06620	0.002728912	0.000623603	0.02639554	518
AT1G54080	0.002282895	0.004100435	0.023410504	519
AT3G05670	0.008577496	0.001100802	0.020136975	520
AT3G01090	0.006370966	0.000347478	0.023099968	521
AT1G27900	0.001379428	0.007518071	0.020931051	522
AT4G13970	0.003721252	0.003655424	0.022451962	523
AT5G06120	0.006206819	0	0.023628698	524
AT3G63270	0.008671174	0	0.021166463	525
AT5G19350	0.003493669	0.002652588	0.0237112	526
AT3G03940	0.002572727	0.00158207	0.025715534	527
AT2G47620	0.002042182	0.002146104	0.025704775	528
AT2G35510	0.003206096	0.001927089	0.024765232	529
AT2G31580	0.001799931	0	0.028114758	530
AT1G67140	0.006741385	0	0.023244104	531
AT1G48310	0.000323781	0.007196276	0.022535462	532
AT1G54170	0.003785401	0.001433379	0.024840333	533
AT1G36990	0.001296335	0.003068271	0.025699958	534
AT2G19340	0.008318346	0	0.021799404	535
AT1G60900	0.012607055	0	0.017538343	536
AT1G13780	0.004789215	0.001067931	0.024310756	537
AT1G78690	0.006472123	0.004468845	0.019248817	538
AT1G03560	0.005827085	0.001910874	0.022505064	539
AT2G27900	0.006078784	0	0.024197908	540
AT2G42670	0.005604591	0.001266081	0.023424856	541
AT1G29840	0.003160763	0.008424682	0.018714027	542

AT1G69220	0.00189928	0.003115335	0.025304678	543
AT1G22970	0.008607566	0.001820852	0.019907407	544
AT4G39910	0.003613931	0.003320981	0.023488125	545
AT5G41910	0.00766696	0.003072076	0.019690989	546
AT2G18245	0.003379358	0.0031623	0.0238936	547
AT5G67580	0.001055666	0.000560343	0.028967673	548
AT1G34120	0.001123317	0.003572619	0.026014554	549
AT3G07080	0.005566633	0.001854696	0.023296862	550
AT1G51965	0.004691559	0.00127213	0.024768232	551
AT1G23465	0.012119419	0.000293687	0.01833681	552
AT5G02850	0.00484667	0.003789612	0.02212959	553
AT1G67170	0.00485869	0.005087181	0.020826106	554
AT5G19840	0.004044636	0.00434634	0.022385463	555
AT2G42247	0.011122301	0	0.019714571	556
AT1G48970	0.002539108	0.001146093	0.027170339	557
AT2G36850	0.009485761	0.00036132	0.021012067	558
AT3G03210	0.006586054	0	0.024280177	559
AT3G12380	0.004787251	0	0.026092533	560
AT1G05860	0.005322618	0.004050449	0.021554912	561
AT3G49050	0.00494055	0.004122216	0.021897835	562
AT3G10380	0.006371164	0.000129329	0.024466719	563
AT1G70180	0.006304001	0.00287017	0.02180004	564
AT2G03070	0.010975198	0.002981994	0.017047646	565
AT1G76185	0.012452474	0	0.018559318	566
AT3G51620	0.002975966	0	0.028037221	567
AT4G26980	0.004985233	0.001424969	0.024620172	568
AT5G10060	0.0053558	0.0039345	0.021745691	569
AT1G69020	0.002948894	0.017537348	0.01058329	570
AT2G25170	0.006102685	0.000499696	0.024487013	571
AT2G25760	0.001281547	0.000354554	0.029485929	572
AT2G14850	0.004052167	0.000624005	0.026449374	573
AT1G78540	0.003867935	0.00303715	0.024249695	574
AT2G36360	0.003438342	0.001755453	0.025965425	575
AT2G26780	0.007334112	6.74E-05	0.023802849	576
AT3G26400	0.004198547	0.000375282	0.026682821	577
AT4G00500	0.002951016	0.003162042	0.025163449	578
AT5G37930	0.004739883	0.004634945	0.021902234	579
AT1G54060	0.004837702	0.00148756	0.025023377	580
AT1G73840	0.007117044	0	0.024237873	581
AT3G14910	0.001740516	0.004474041	0.02514809	582
AT3G12590	0.008039803	0	0.023336545	583
AT2G18770	0.00920573	0	0.022277823	584

AT1G58060	0.007594259	0.001988236	0.021907026	585
AT1G59610	0.004496333	0.001315919	0.025685151	586
AT3G02860	0.004790842	0.002522705	0.024185651	587
AT3G19280	0.013479144	0	0.018047968	588
AT3G45890	0.006326591	0.005187114	0.020032418	589
AT2G38440	0.009274412	0.00033693	0.021938715	590
AT3G08850	0.009071997	0	0.022524744	591
AT1G10580	0.004174797	0.004776503	0.022715313	592
AT2G19470	0.004310387	0.005593921	0.0217628	593
AT3G22990	0.002094079	3.67E-05	0.029570972	594
AT4G03430	0.003704853	0.000625465	0.027450302	595
AT2G04560	0.005870427	0.005279652	0.020670872	596
AT5G65570	0.005787468	0.00206284	0.023980778	597
AT5G16280	0.00726841	0	0.024580026	598
AT1G66660	0.009328892	7.35E-16	0.022548334	599
AT1G18950	0.002241509	0.001282077	0.028353671	600
AT4G34430	0.006029002	0.004349079	0.021499354	601
AT5G64600	0.012237124	0.006981232	0.0126636	602
AT1G49040	0.005091458	0.000799462	0.026016668	603
AT5G13310	0.005622354	0.000193024	0.026101602	604
AT3G22470	0.004338035	0.001100569	0.026520026	605
AT3G50080	0.003380169	0.001719824	0.026865001	606
AT5G43940	0.010151431	0.001408893	0.020452498	607
AT1G77140	0.003350583	0.003782616	0.024904153	608
AT5G55260	0.004141884	0.004470185	0.023487371	609
AT5G24340	0.000561118	0.003006773	0.028561052	610
AT4G24200	0.00415764	0.002281547	0.025694624	611
AT3G51640	0.007543532	0.00367558	0.020963685	612
AT4G20410	0.005606925	0.004452362	0.022165764	613
AT1G21700	0.003622691	0	0.028608705	614
AT2G32250	0.005398885	0.000386627	0.026497557	615
AT1G19580	0.005441975	0.000150663	0.026712008	616
AT5G15270	0.005928666	0.00337695	0.023011568	617
AT5G64960	0.006562043	0.004941034	0.020821263	618
AT3G01340	0.007191112	0	0.025162121	619
AT5G47480	0.008290987	0	0.024141713	620
AT4G38600	0.010137562	0	0.02230686	621
AT4G16430	0.002754115	0.000921537	0.028808587	622
AT1G51450	0.000788214	0.001594507	0.030109428	623
AT4G24440	0.016278325	1.32E-16	0.016218416	624
AT5G40710	0.004380281	0.003488449	0.024644688	625
AT5G42920	0.00228983	0.003950357	0.02629554	626

AT4G25680	0.003629554	0.000737382	0.028177626	627
AT1G67890	0.004220803	0	0.028354524	628
AT3G10730	0.004104902	0.000113046	0.028360057	629
AT3G16090	0.002418178	0.005871213	0.024308019	630
AT3G54380	0.003817859	0.003640739	0.025167764	631
AT2G26920	0.001701357	0.001827793	0.029103051	632
AT5G66820	0.004480548	0.009381446	0.018790232	633
AT4G23460	0.003305979	0.001909895	0.027463684	634
AT5G58270	0.002233058	0.001883239	0.028568561	635
AT3G18380	0.004255478	0.001623397	0.026814517	636
AT5G24360	0.004927183	0.003575168	0.024198645	637
AT2G30530	0.004307629	0.000700846	0.027757216	638
AT1G15280	0.008645653	0.001925309	0.022197556	639
AT3G63150	0.003768147	0	0.029004899	640
AT5G49880	0.001387074	0.006734423	0.024705545	641
AT5G65940	0.006864743	0.002392336	0.023590884	642
AT3G24200	0.000875436	0.002912298	0.029075775	643
AT5G58240	0.006038093	0.002103817	0.024731505	644
AT1G62860	0.008780879	0	0.024171113	645
AT2G32260	0.003531431	0.005925973	0.02350264	646
AT1G08620	0.009064477	0	0.023905869	647
AT4G14850	0.008391464	0.003085221	0.021529004	648
AT4G15415	0.00333715	0.000935313	0.028823116	649
AT1G48380	0.002263781	0.004313849	0.02654465	650
AT1G10820	0.004008234	0.001832538	0.027360811	651
AT2G27860	0.010698847	0.004170252	0.018425126	652
AT5G61150	0.003759323	0.001044024	0.028593809	653
AT5G07120	0.002502162	0.003598194	0.027316401	654
AT2G47350	0.001719962	0.0027252	0.029052721	655
AT2G41530	0.008717218	0.010568684	0.014219971	656
AT1G12930	0.007207854	0.000106538	0.026216546	657
AT3G55140	0.005745223	0.003217916	0.024586692	658
AT5G49830	0.003365914	0.003513352	0.026710837	659
AT2G15560	0.002212276	0.000206112	0.031200826	660
AT5G27970	0.005578156	0.000857775	0.027204609	661
AT2G04550	0.007551661	0.006081282	0.020017713	662
AT4G29960	0.00355418	0.004704684	0.025431109	663
AT1G15220	0.005137676	0	0.028589335	664
AT3G43590	0.005226003	0.012205641	0.016297147	665
AT1G03910	0.002556058	0.003314654	0.027912302	666
AT3G10440	0.002780569	0.00397894	0.027023773	667
AT5G40280	0.00248037	0	0.031303824	668

AT5G23390	0.002421282	0.001339135	0.030056245	669
AT2G16405	0.002012891	0.004654337	0.027175146	670
AT4G32050	0.013802283	8.54E-05	0.01998892	671
AT5G51400	0.004976356	0.003684427	0.025226762	672
AT1G28060	0.004023852	0.008873458	0.021008322	673
AT3G22430	0.002386153	0.004243527	0.027296801	674
AT3G09880	0.004591843	0.00259147	0.026775349	675
AT5G19660	0.002581144	0.000499214	0.030895757	676
AT3G26920	0.007089943	0.002139009	0.024752546	677
AT3G19520	0.008244207	0.009456653	0.016348171	678
AT4G08940	0.017442481	0.008287329	0.0083278	679
AT2G47900	0.008260053	0.002775556	0.023031358	680
AT4G26000	0.003084223	0.002713284	0.028312695	681
AT1G26640	0.005387728	0.002634625	0.026102905	682
AT1G72390	0.007578919	0.000244109	0.026324788	683
AT3G03140	0.005035505	0.003284219	0.025853413	684
AT4G21660	0.002623081	0.004797207	0.026778039	685
AT4G00170	0.010242198	0	0.023986136	686
AT3G26640	0.006302426	0	0.02794937	687
AT5G37340	0.0028929	0.008893306	0.022498798	688
AT5G16310	0.00730358	0.003689253	0.023293895	689
AT4G22150	0.008444813	0.001119186	0.024793731	690
AT4G05420	0.006289217	0	0.028089665	691
AT4G11860	0.004064162	0.004071806	0.026311065	692
AT4G24680	0.009723395	0	0.024737231	693
AT1G71350	0.001306319	0.001187453	0.031988718	694
AT5G18410	0.006713697	0	0.027776829	695
AT5G50970	0.004007612	0.006626859	0.023891313	696
AT1G75080	0.001593375	0.011061401	0.021898409	697
AT4G37880	0.010911603	0.001753973	0.021933496	698
AT5G51300	0.007628833	0	0.026980533	699
AT3G29130	0.008682574	0.000975202	0.025010248	700
AT5G20660	0.006932298	0.000910095	0.026859873	701
AT3G29160	0.012802495	0.003684459	0.018235521	702
AT3G52260	0.005839858	0.002368741	0.026532141	703
AT3G06720	0.007351689	0.00768745	0.019734824	704
AT3G52030	0.004008492	0.0004952	0.030322724	705
AT3G05940	0.002261764	0.004373774	0.028192349	706
AT1G79070	0.013277345	0	0.021583127	707
AT5G22120	0.002805397	0	0.032076151	708
AT2G19950	0.003100811	0.001501844	0.030287511	709
AT3G14400	0.002806047	0.002212914	0.029932997	710

AT1G67500	0.008536125	0	0.026438527	711
AT3G62330	0.001817169	0.006117338	0.027048665	712
AT4G05460	0.01034251	0.003006469	0.021659153	713
AT2G17410	0.006588505	0.001842431	0.026608172	714
AT1G53770	0.001368253	0.004030021	0.029647997	715
AT3G05530	0.002493743	0.000327449	0.032250637	716
AT1G04950	0.00496947	0.002746078	0.027387506	717
AT3G50880	0.005334694	0.002050005	0.027727466	718
AT5G58470	0.008758836	0	0.026376136	719
AT2G22370	0.011949484	0.001287346	0.021904872	720
AT5G50230	0.002032834	0.007516794	0.02561381	721
AT3G61415	0.006576217	0.00258027	0.026019269	722
AT4G19600	0.003797374	0.00107903	0.03030402	723
AT5G42080	0.002408641	0.002469008	0.030332117	724
AT2G33835	0.000134072	0.001480172	0.033596296	725
AT5G01470	0.018492804	0	0.016736277	726
AT5G11490	0.004703465	8.71E-05	0.030445964	727
AT3G05510	0.000962255	0.002750361	0.031600175	728
AT3G58630	0.005246954	0.0059684	0.024134775	729
AT5G66290	0.004558171	0	0.030796024	730
AT1G60670	0.003750868	0	0.031608918	731
AT1G79030	0.007132391	0.001201414	0.027045235	732
AT5G65830	0.005361711	0.002792855	0.027291578	733
AT5G41330	0.005943135	0	0.029577858	734
AT5G01460	0.007579406	0.008035996	0.019928962	735
AT3G06190	0.005529296	0.006106018	0.023960053	736
AT5G09410	0.006231798	0	0.029368609	737
AT3G03300	0.006548627	0.001729649	0.027337829	738
AT5G43560	0.005326579	0	0.030301859	739
AT4G38180	0.004285176	0.004875495	0.026474079	740
AT4G22350	0.014458179	0	0.021196434	741
AT1G73100	0.003187486	0.00127561	0.031214256	742
AT4G04885	0.003136672	0.002070499	0.030519865	743
AT1G29220	0.003235581	0.000775733	0.031727453	744
AT3G20800	0.001809825	0.00216092	0.031797294	745
AT5G05610	0.005609527	0	0.030158841	746
AT3G57340	0.003930385	0.003754758	0.028145586	747
AT2G25560	0.003309601	0	0.032522667	748
AT1G06200	0.008213861	0.001879484	0.025755737	749
AT4G17620	0.001806828	0.001080567	0.032988501	750
AT1G50620	0.003709302	0.003468391	0.028702774	751
AT1G13020	0.005706606	0.001560766	0.028625733	752

AT1G15490	0.007315383	0.003986762	0.024592851	753
AT5G08390	0.010404987	0	0.025491473	754
AT1G07470	0.006478674	0.003810302	0.025621325	755
AT1G64990	0.002772199	0.000448507	0.032714726	756
AT1G55830	0.002500355	0	0.03347352	757
AT4G36630	0.006121102	0	0.029891918	758
AT5G12430	0.008137677	0.000900007	0.027024047	759
AT3G12140	0.00298899	0.002256669	0.030828266	760
AT3G21060	0.00593452	0.001662657	0.028513567	761
AT3G09800	0.006242252	0.010571383	0.019373358	762
AT4G32960	0.008379428	0.001473592	0.02634426	763
AT1G19120	0.009362738	0	0.026843839	764
AT5G04070	0.006238443	0.001953674	0.028033506	765
AT1G08125	0.004114845	0.000314145	0.031799	766
AT4G00420	0.004949577	0.000351314	0.030942095	767
AT5G63870	0.004660471	0.001933405	0.02970168	768
AT5G47430	0.004340214	0.001226907	0.030736146	769
AT1G07705	0.002360406	0.007491842	0.026472893	770
AT4G25440	0.003493336	0.000371814	0.032510957	771
AT3G51650	0.010423194	0	0.025971322	772
AT1G02990	0.009094192	0	0.027310918	773
AT5G63610	0.003428997	0.003185978	0.02980434	774
AT1G73970	0.011413309	0.001737168	0.023311918	775
AT4G17410	0.006672488	0.002608636	0.027216002	776
AT1G43900	0.003870286	0.00347443	0.029168532	777
AT3G47390	0.001786884	0.002143126	0.032593453	778
AT3G63070	0.01032998	0	0.026196828	779
AT4G32680	0.002902279	0.000375496	0.033321069	780
AT4G21670	0.009451002	0.011032315	0.016122189	781
AT5G10350	0.007069605	0.003590476	0.02596404	782
AT5G44090	0.00475672	0.005833213	0.026037994	783
AT5G56190	0.002403124	0.001576354	0.032652467	784
AT3G07190	0.005387046	0.002051773	0.029198615	785
AT1G16825	0.009575499	0.001298567	0.025775861	786
AT4G40050	0.002817887	0.005867015	0.027999946	787
AT2G18760	0.008309629	0.000991398	0.027403877	788
AT5G07970	0.005729024	0.001707407	0.02927227	789
AT5G06950	0.001802259	3.89E-05	0.034877038	790
AT5G45190	0.002066366	0.003318833	0.031359825	791
AT5G51170	0.013074331	0.012072034	0.011615797	792
AT5G27720	0.00627295	0	0.030517903	793
AT1G65430	0.002396851	0.00068573	0.033775822	794

AT1G01950	0.002749255	0.001721989	0.032421882	795
AT1G06070	0.001604901	0.005869773	0.02941967	796
AT4G27040	0.006196969	0.007087088	0.023691335	797
AT2G34980	0.004074856	0.002613372	0.030309314	798
AT2G27100	0.00586587	0	0.031141558	799
AT3G53710	0.003755978	0.002528829	0.030743248	800
AT4G29910	0.000882376	0.011346811	0.024837952	801
AT1G06410	0.020801574	0	0.016269403	802
AT5G04060	0.002721555	0.003955208	0.030401143	803
AT4G32560	0.004331105	0.000927283	0.031841568	804
AT1G30290	0.005605393	0.002108097	0.02939753	805
AT1G12620	0.003343987	0.001686965	0.032086121	806
AT3G19950	0.005541498	0.001488676	0.030117379	807
AT5G10710	0.007128137	0.003240099	0.026780008	808
AT1G78800	0.001137899	0.002348928	0.033698342	809
AT5G55060	0.009294922	0.000338262	0.027557285	810
AT1G32750	0.008141534	0	0.029098518	811
AT1G66340	0.001517569	0.000765849	0.035025488	812
AT5G04940	0.004359917	0.00052985	0.032434021	813
AT3G19770	0.005502464	0	0.031823793	814
AT1G66345	0.008724636	0.010372414	0.018265532	815
AT4G17330	0.012163653	0	0.025205184	816
AT5G48990	0.010009449	0.003986242	0.023426658	817
AT1G03350	0.00901978	0.001062404	0.027342349	818
AT3G47940	0.006446296	0.000488368	0.030515729	819
AT2G41790	0.002900963	0.000514322	0.03406544	820
AT4G17895	0.001030548	0.001849411	0.034605859	821
AT3G06440	0.003628089	0.007856199	0.02600369	822
AT3G27320	0.004046309	0.002554635	0.030924418	823
AT1G03280	0.003596486	7.76E-05	0.033863534	824
AT1G19430	0.007346301	0.000467795	0.029770251	825
AT5G66010	0.014577705	0	0.023008989	826
AT3G33520	0.003517981	0.002617749	0.031456506	827
AT1G27600	0.004713396	0.004789394	0.028094693	828
AT2G43210	0.002813298	0.004870586	0.029935888	829
AT4G06634	0.004357069	0.002798002	0.030491252	830
AT1G47750	0.013270409	0.002670383	0.021748378	831
AT4G30996	0.010402564	0.006420889	0.020893072	832
AT2G17200	0.004918773	0.001199263	0.031610225	833
AT3G04605	0.004433493	0.002500981	0.030809426	834
AT3G19630	0.003517173	0.00786517	0.026395879	835
AT5G39450	0.002815163	0.002977259	0.032039754	836

AT3G12100	0.004613389	0	0.033247316	837
AT1G52520	0.007462552	0.00327896	0.027133496	838
AT1G27750	0.009842455	5.12E-05	0.027997964	839
AT4G10070	0.015362807	0	0.02253351	840
AT4G35530	0.009619414	0	0.028279619	841
AT1G54440	0.003409852	0.001546274	0.032963226	842
AT4G16250	0.007196049	0.011505334	0.019258072	843
AT1G30970	0.008006305	3.12E-05	0.029922023	844
AT2G46200	0.005830715	0.002583217	0.029624841	845
AT3G49510	0.01292133	0.004362611	0.020783744	846
AT3G06350	0.004826282	0.004198118	0.029059623	847
AT5G65260	0.011034577	0	0.027051758	848
AT1G20560	0.008027617	0.00299116	0.027069653	849
AT4G21710	0.006172045	0.002264715	0.029678966	850
AT1G73200	0.006388217	0.000405714	0.031332683	851
AT1G55460	0.005116329	0.003585994	0.029434687	852
AT5G04910	0.012915022	0	0.025267846	853
AT5G57950	0.008942564	0	0.029277491	854
AT3G48810	0.002340936	0.002236154	0.033679707	855
AT5G38380	0.001743088	0.004934885	0.031605025	856
AT1G50700	0.012250477	6.61E-18	0.026034105	857
AT1G24300	0.008762348	0	0.029590829	858
AT2G26590	0.003478517	0.000716942	0.034159518	859
AT5G39250	0.011324998	0.000305525	0.026733283	860
AT1G60560	0.002554924	0.004782836	0.031090221	861
AT5G18190	0.00170488	0.003173237	0.033577406	862
AT5G62640	0.003195655	0.003011556	0.032258806	863
AT3G56210	0.008862193	0.00243235	0.02722171	864
AT1G16010	0.005286657	0.00202432	0.031214529	865
AT3G60410	0.003672302	0.001773241	0.033090559	866
AT2G28060	0.007705728	0	0.030839583	867
AT5G65960	0.00327426	0.003485462	0.031787845	868
AT1G58220	0.005910005	0	0.032672625	869
AT4G09680	0.005102035	0	0.033499372	870
AT5G17510	0.007512429	0.004386299	0.026779687	871
AT3G16230	0.005504524	0.017806373	0.015407233	872
AT1G55880	0.004875179	0.004999945	0.028865915	873
AT2G26470	0.009357826	0.010363338	0.019046501	874
AT3G10650	0.010427221	0.001358828	0.026986158	875
AT1G51540	0.012250538	0.001945687	0.024580964	876
AT2G44525	0.006619086	0.001888641	0.030290827	877
AT3G03740	0.001071141	0.00010206	0.037637488	878

AT5G48520	0.001112366	0.001159357	0.036559988	879
AT4G39240	0.00531546	0.002617247	0.03098527	880
AT3G10540	0.003898099	0.004206751	0.030827736	881
AT4G27060	0.003739592	0.000745543	0.0344599	882
AT2G07360	0.005304784	0.000110165	0.033565739	883
AT5G08510	0.007087834	0.005701476	0.026194337	884
AT1G25420	0.008324561	0.007146126	0.023587315	885
AT1G15920	0.006756408	0.003459559	0.028856423	886
AT1G69460	0.009108311	0.00162189	0.0283447	887
AT5G42620	0.004274096	0.003538852	0.031281584	888
AT1G67325	0.011647846	0	0.027459796	889
AT5G06830	0.003241419	0.003208115	0.032671263	890
AT1G75510	0.011001689	2.67E-05	0.028128342	891
AT2G35230	0.008468364	0.006467886	0.024222094	892
AT3G51610	0.006888954	6.08E-05	0.032214272	893
AT5G49000	0.002068041	0	0.037108779	894
AT5G09740	0.004130788	0.00127299	0.033777436	895
AT5G51230	0.000828657	0.00172411	0.036634429	896
AT5G23490	0.005902543	0.001998513	0.031317892	897
AT2G37585	0.011465907	0.008457273	0.019297647	898
AT3G10390	0.005934092	0.000723303	0.032573861	899
AT2G47970	0.005832329	0	0.033425487	900
AT3G06940	0.00250671	0.001352248	0.035430286	901
AT2G26060	0.008452515	0.001476938	0.029390639	902
AT4G32010	0.005535624	0	0.033813205	903
AT1G78810	0.001127967	0.008560932	0.029667276	904
AT5G12310	0.011806661	0.002022264	0.025537753	905
AT3G12130	0.00523567	0.000324561	0.033830592	906
AT2G32840	0.00563876	0.003975347	0.029782869	907
AT5G15400	0.0077799	0.004411574	0.027253627	908
AT5G27460	0.009992153	0.003913946	0.025541302	909
AT1G48900	0.002512589	0.003107891	0.033844074	910
AT3G60800	0.004868989	0.000914706	0.033761904	911
AT2G14530	0.006687457	0	0.032873151	912
AT5G06910	0.016389595	0.002479902	0.020726152	913
AT3G13290	0.00791826	0.001698673	0.02999514	914
AT2G10950	0.002237768	0.002036178	0.03534209	915
AT4G36480	0.004059245	0.001291651	0.034282493	916
AT2G26660	0.008370064	0.007766177	0.023507563	917
AT4G20330	0.004780142	0.001929287	0.032943524	918
AT5G37370	0.006900434	0.001111697	0.031673839	919
AT3G13350	0.004312979	0.003823688	0.031597688	920

AT5G54520	0.007586634	0.010241617	0.021909046	921
AT3G07020	0.003617593	0.005216934	0.030946362	922
AT1G49480	0.007966587	4.13E-05	0.031828328	923
AT5G15570	0.002420302	0.002867785	0.034555262	924
AT4G04860	0.004235605	0.000562559	0.035046793	925
AT1G66750	0.006707714	0.005237151	0.027929898	926
AT3G21295	0.003732641	0.003198763	0.032944011	927
AT1G63490	0.006781772	0	0.033112704	928
AT3G57570	0.004506944	0.001486978	0.033907989	929
AT4G31410	0.002268389	0.005050607	0.032583154	930
AT5G06260	0.005317165	0	0.034590448	931
AT1G08710	0.002706672	0.006179798	0.031026013	932
AT1G56420	0.006972276	0.000583681	0.032361027	933
AT1G54650	0.00439823	0.000462422	0.035096723	934
AT2G45200	0.006386251	0.002768613	0.03081204	935
AT3G47850	0.004507791	0.000895668	0.034584806	936
AT5G19330	0.003751022	0.003541183	0.032715123	937
AT3G04610	0.007141051	0.005905541	0.026979511	938
AT3G19980	0.002904848	0.001277083	0.035847846	939
AT5G05950	0.01112472	0.004352517	0.024555405	940
AT4G15475	0.004205863	0.003274415	0.032556191	941
AT3G14010	0.004904597	0.003623836	0.031533324	942
AT1G10350	0.009260591	0.000381941	0.030486678	943
AT4G38130	0.004774726	0.00733211	0.028040912	944
AT5G49610	0.004013654	0.003177499	0.032978141	945
AT1G35660	0.010737586	0.003006202	0.02644648	946
AT5G42360	0.007259604	0.005458734	0.027491911	947
AT5G58040	0.005727944	0.003261943	0.031236236	948
AT2G25320	0.012105747	0	0.028152647	949
AT2G22090	0.003335652	0.001712756	0.035231986	950
AT5G27650	0.008169508	0.005543114	0.02658911	951
AT1G22660	0.002874884	0.002340382	0.035136317	952
AT5G62880	0.005688449	0.003233665	0.031474713	953
AT5G56240	0.011123464	0.000273841	0.029010262	954
AT4G38980	0.00948334	0.005385884	0.025604365	955
AT5G08630	0.003255716	0.000909284	0.03633525	956
AT4G38270	0.002887133	0.001269633	0.036370146	957
AT1G09040	0.003261899	0.00756832	0.029722151	958
AT3G54280	0.012368439	0.003334891	0.02486491	959
AT1G66260	0.007720874	0.004443165	0.02843139	960
AT5G15540	0.006953378	0	0.033649709	961
AT5G19830	0.005001858	0.002835286	0.032792419	962

AT3G20870	0.004896051	0.005034632	0.030705523	963
AT5G53060	0.008767864	0.003104327	0.028770761	964
AT4G29490	0.003567828	0.001312809	0.035772317	965
AT4G17910	0.004718084	0.003978309	0.031986293	966
AT5G35995	0.000331299	0.00173868	0.038615933	967
AT4G19990	0.005686874	0.003880147	0.03112234	968
AT2G22310	0.004813995	0.007846874	0.028031004	969
AT2G13840	0.008351002	0.008614779	0.02376805	970
AT1G59510	0.001667302	0.011568845	0.02750218	971
AT5G11730	0.001330637	0.004343703	0.03506948	972
AT1G65580	0.004519416	0.001313727	0.034911884	973
AT2G19270	0.00273893	0.003203479	0.034814068	974
AT2G13370	0.010098817	0	0.030693685	975
AT4G32640	0.004723579	0	0.036159826	976
AT1G48110	0.00514251	0.006081537	0.029763664	977
AT2G36720	0.004523022	0	0.036477825	978
AT1G80260	0.004350455	0	0.036675952	979
AT5G05700	0.003212611	0.001596608	0.036274577	980
AT2G30105	0.003917022	0.003325814	0.033852767	981
AT2G46920	0.00489597	0.005496942	0.030724317	982
AT5G35980	0.006919551	0	0.034228103	983
AT3G32940	0.004275867	0.004659222	0.03222841	984
AT1G17720	0.004503158	0.003190621	0.033513265	985
AT1G45233	0.002682524	0.000904243	0.037622206	986
AT3G52640	0.003963212	0.002503451	0.034753113	987
AT5G19485	0.001762271	0.003789084	0.03569906	988
AT2G30910	0.006401117	0.004380977	0.030493433	989
AT3G24350	0.003789655	0.002970254	0.034523404	990
AT5G35753	0.006592428	0.001366298	0.033329821	991
AT1G77180	0.005348404	0.005054044	0.030910785	992
AT5G49580	0.000921772	0.001809362	0.038582941	993
AT1G18160	0.004994655	0.000508342	0.035814896	994
AT1G72560	0.007996596	0.003676719	0.029650383	995
AT1G29260	0.011630952	0.003944768	0.02576518	996
AT1G07480	0.013904413	0.010340018	0.017099823	997
AT3G45100	0.006202436	0.007706845	0.02744602	998
AT3G18790	0.009825071	0	0.031557677	999
AT4G01040	0.003907537	0.009893988	0.027608771	1000
