# Supplementary material for: Identifying stably expressed genes from multiple RNA-Seq data sets
Source: PeerJ. 2016 Dec 20;4:e2791. doi: 10.7717/peerj.2791 (PMC5178351; doi:10.7717/peerj.2791)
Supplement: Table S5 [file peerj-04-2791-s005.pdf]

Supplemental Table S5: The overlapped 104 genes among top 1000 genes from the seedling, the leaves and the multiple tissue group.

Gene	Rank_in_seedling	Rank_in_leaf	Rank_in_tissue
AT1G07980	377	279	293
AT1G08750	871	14	373
AT1G09020	505	41	372
AT1G11630	943	884	462
AT1G12470	241	776	177
AT1G13320	159	112	513
AT1G15490	375	295	753
AT1G17720	7	693	985
AT1G17760	820	415	107
AT1G19430	723	798	825
AT1G19480	696	181	175
AT1G22200	29	109	266
AT1G35470	5	787	22
AT1G43190	624	166	242
AT1G45233	52	777	986
AT1G50500	421	893	215
AT1G51450	613	46	623
AT1G52630	332	68	65
AT1G54080	362	44	519
AT1G54610	40	16	51
AT1G55170	201	191	509
AT1G56590	122	847	312
AT1G60670	680	170	731
AT1G61150	49	958	455
AT1G63430	640	541	363
AT1G65430	499	824	794
AT1G66750	559	9	926
AT1G69340	107	796	410
AT1G71350	28	242	694
AT1G72340	901	334	156
AT1G73730	631	450	515
AT1G77140	322	919	608
AT1G78800	70	272	809
AT1G79810	527	173	421
AT2G05755	292	251	296
AT2G20790	147	967	181
AT2G22370	898	194	720
AT2G23080	76	529	355

AT2G26590	304	22	859
AT2G27350	317	788	54
AT2G30880	48	76	114
AT2G32170	407	358	276
AT2G39760	923	807	174
AT2G40090	289	907	140
AT2G41350	572	47	432
AT2G45690	749	224	66
AT3G03740	284	834	878
AT3G03940	1	286	527
AT3G10330	172	218	391
AT3G10540	562	537	881
AT3G10730	551	156	629
AT3G17205	548	721	239
AT3G17880	667	632	196
AT3G18380	466	275	636
AT3G18790	155	510	999
AT3G19980	514	310	939
AT3G20650	57	605	304
AT3G27320	325	314	823
AT3G28670	227	1	449
AT3G33530	965	934	444
AT3G50860	610	115	217
AT3G53390	255	638	325
AT3G60410	456	347	866
AT3G63150	11	106	640
AT4G00650	491	293	176
AT4G01370	220	853	256
AT4G13730	88	12	514
AT4G15415	886	212	649
AT4G15880	175	783	185
AT4G17020	177	569	44
AT4G24470	116	941	179
AT4G24530	427	211	516
AT4G24550	274	318	289
AT4G26100	441	201	15
AT4G32010	539	539	903
AT4G32120	78	98	332
AT4G39240	370	468	880
AT5G09860	397	828	102
AT5G18190	34	487	862
AT5G18230	356	144	116

AT5G18410	852	956	695
AT5G19280	856	134	98
AT5G19350	580	654	526
AT5G19485	635	174	988
AT5G20930	779	740	69
AT5G21010	298	532	453
AT5G26760	621	729	425
AT5G41150	537	576	115
AT5G43320	565	957	295
AT5G44150	733	66	117
AT5G46210	748	909	100
AT5G46630	679	898	201
AT5G48520	747	67	879
AT5G49580	37	583	993
AT5G51280	157	914	383
AT5G51340	502	457	42
AT5G53180	492	343	29
AT5G57950	740	341	854
AT5G58100	366	195	7
AT5G58270	214	309	635
AT5G59710	189	652	205
AT5G64470	75	57	108
AT5G65260	760	476	848
AT5G67580	376	494	548
