# Supplementary material for: Identifying stably expressed genes from multiple RNA-Seq data sets
Source: PeerJ. 2016 Dec 20;4:e2791. doi: 10.7717/peerj.2791 (PMC5178351; doi:10.7717/peerj.2791)
Supplement: Table S6 — Description of the 9 genes that are overlapped between the 104 genes (see Supplemental Table S5) and the top 100 stably expressed genes identified by Czechowski et al. from a developmental series of microarray study. Source: TAIR (https://www.arabidopsis.org/servlets/Search?type=gene&action=new_search) [file peerj-04-2791-s006.pdf]

Supplemental Table S6: Description of the 9 genes that are overlapped between the 104 genes (see Supplemental Table S5) and the top 100 stably expressed genes identified by Czechowski et al. from a developmental series of microarray study. Source: TAIR (https://www.arabidopsis.org/servlets/Search?type=gene&action=new_search)

Gene	Gene Model Type	Description
AT1G13320	protein_coding	one of three genes encoding the 65 kDa regulatory subunit of protein phosphatase 2A (PP2A)
AT1G54080	protein_coding	oligouridylate-binding protein 1A (UBP1A); FUNCTIONS IN: mRNA 3'-UTR binding; LOCATED IN: plasma membrane; EXPRESSED IN: 24 plant structures; EXPRESSED DURING: 14 growth stages; CONTAINS InterPro DOMAIN/s: RNA recognition motif, RNP-1 (InterPro:IPR000504), Nucleotide-binding, alpha-beta plait (InterPro:IPR012677); BEST Arabidopsis thaliana protein match is: RNA-binding (RRM/RBD/RNP motifs) family protein (TAIR:AT3G14100.1)
AT2G20790	protein_coding	clathrin adaptor complexes medium subunit family protein; INVOLVED IN: intracellular protein transport, vesicle-mediated transport; LOCATED IN: clathrin adaptor complex; EXPRESSED IN: 23 plant structures; EXPRESSED DURING: 13 growth stages; CONTAINS InterPro DOMAIN/s: Clathrin adaptor, mu subunit, conserved site (InterPro:IPR018240), Clathrin adaptor, mu subunit, C-terminal (InterPro:IPR008968)
AT2G32170	protein_coding	S-adenosyl-L-methionine-dependent methyltransferases superfamily protein; LOCATED IN: cellular_component unknown; EXPRESSED IN: 24 plant structures; EXPRESSED DURING: 15 growth stages; CONTAINS InterPro DOMAIN/s: N2227-like (InterPro:IPR012901); BEST Arabidopsis thaliana protein match is: S-adenosyl-L-methionine-dependent methyltransferases superfamily protein (TAIR:AT2G32160.3)

AT3G10330	protein_coding	Cyclin-like family protein; FUNCTIONS IN: RNA polymerase II transcription factor activity, transcription regulator activity, zinc ion binding, translation initiation factor activity; INVOLVED IN: translational initiation, regulation of transcription, DNA-dependent, transcription initiation, regulation of transcription; LOCATED IN: nucleus, cytoplasm; EXPRESSED IN: 23 plant structures; EXPRESSED DURING: 14 growth stages; CONTAINS InterPro DOMAIN/s: Transcription factor TFIIB related (InterPro:IPR000812), Cyclin-like (InterPro:IPR011028), Transcription factor TFIIB, cyclin-related (InterPro:IPR013150), Cyclin-related (InterPro:IPR013763), Zinc finger, TFIIB-type (InterPro:IPR013137), Cyclin (InterPro:IPR006670); BEST Arabidopsis thaliana protein match is: transcription factor IIB (TAIR:AT2G41630.1)
AT4G24550	protein_coding	Clathrin adaptor complexes medium subunit family protein; INVOLVED IN: intracellular protein transport, transport, vesicle-mediated transport; LOCATED IN: clathrin vesicle coat, clathrin adaptor complex; EXPRESSED IN: 22 plant structures; EXPRESSED DURING: 13 growth stages; CONTAINS InterPro DOMAIN/s: Clathrin adaptor, mu subunit, C-terminal (InterPro:IPR008968), Clathrin adaptor, mu subunit (InterPro:IPR001392), Longin-like (InterPro:IPR011012); BEST Arabidopsis thaliana protein match is: Clathrin adaptor complexes medium subunit family protein
AT5G26760	protein_coding	unknown protein; CONTAINS InterPro DOMAIN/s: Protein of unknown function DUF408 (InterPro:IPR007308)
AT5G46210	protein_coding	Arabidopsis CULLIN4 (CUL4) forms an E3 ubiquitin ligase with the CDD complex and a common catalytic subunit RBX1 in mediating light control of development. This CUL4-based E3 ligase is essential for the repression of photomorphogenesis. The partial loss of CUL4 function resulted in a constitutive photomorphogenic phenotype with respect to morphogenesis and light-regulated gene expression. CUL4 exhibits a synergistic genetic interaction with COP10 and DET1.
AT5G46630	protein_coding	clathrin adaptor complexes medium subunit family protein, contains Pfam profile: PF00928 adaptor complexes medium subunit family; similar to micro-adaptins of clathrin coated vesicle adaptor complexes
